# Coupled influence of precipitation and vegetation on millennial-scale erosion rates derived from ^10^Be

**DOI:** 10.1371/journal.pone.0211325

**Published:** 2019-01-25

**Authors:** Ashish Kumar Mishra, Christa Placzek, Rhondda Jones

**Affiliations:** 1 Geosciences, College of Science and Engineering and Centre for Tropical Environmental and Sustainability Science (TESS), James Cook University, Townsville, Queensland, Australia; 2 StatsHelp Service, Graduate Research School, James Cook University, Townsville, Queensland, Australia; Universita degli Studi di Milano, ITALY

## Abstract

Water is one of the main agent of erosion in many environmental settings, but erosion rates derived from beryllium-10 (^10^Be) suggests that a relationship between precipitation and erosion rate is statistically non-significant on a global scale. This might be because of the strong influence of other variables on erosion rate. In this global ^10^Be compilation, we examine if mean annual precipitation has a statistically significant secondary control on erosion rate. Our secondary variable assessment suggests a significant secondary influence of precipitation on erosion rate. This is the first time that the influence of precipitation on ^10^Be-derived erosion rate is recognized on global scale. In fact, in areas where slope is <200m/km (~11°), precipitation influences erosion rate as much as mean basin slope, which has been recognized as the most important variable in previous ^10^Be compilations. In areas where elevation is <1000m and slope is <11°, the correlation between precipitation and erosion rate improves considerably. These results also suggest that erosion rate responds to change in mean annual precipitation nonlinearly and in three regimes: 1) it increases with an increase in precipitation until ~1000 mm/yr; 2) erosion rate stabilizes at ~1000 mm/yr and decreases slightly with increased precipitation until ~2200 mm/yr; and 3) it increases again with further increases in precipitation. This complex relationship between erosion rate and mean annual precipitation is best explained by the interrelationship between mean annual precipitation and vegetation. Increased vegetation, particularly the presence of trees, is widely recognized to lower erosion rate. Our results suggest that tree cover of 40% or more reduces erosion rate enough to outweigh the direct erosive effects of increased rainfall. Thus, precipitation emerges as a stronger secondary control on erosion rate in hyper-arid areas, as well as in hyper-wet areas. In contrast, the regime between ~1000 and ~2200 mm/yr is dominated by opposing relationships where higher rainfall acts to increase erosion rate, but more water also increases vegetation/tree cover, which slows erosion. These results suggest that when interpreting the sedimentological record, high sediment fluxes are expected to occur when forests transition to grasslands/savannahs; however, aridification of grasslands or savannahs into deserts will result in lower sediment fluxes. This study also implies that anthropogenic deforestation, particularly in regions with high rainfall, can greatly increase erosion.

## Introduction

The interrelated processes of erosion and weathering are critical components of Earth’s biogeochemical cycles because they regulate the supply of sediments and nutrients to soils, streams, and ultimately the ocean [1, 2]. Over geologic timescales, the chemical components of erosion and weathering are crucial in understanding climatic evolution [3, 4–8]. Erosion also leads to long-term transformation and development of landscapes [9, 10–17] and is closely associated with sediment yield, which controls the volume and characteristics of material preserved in the rock record. Sediment yield can also have important environmental management implications because the quantity of sediment moving out of a catchment is important for water quality [2, 18–20]. Therefore, it is important to understand the factors that impact erosion, in order to understand biogeochemical cycles [1, 2], interpret the sediment record [21, 22], implement effective land-use strategies, [2, 23, 24] and quantify human influences [18, 19, 25, 26].

Early geomorphic studies suggest that rapid uplift [27], higher relief [28], and/or shifts in climate [21] can result in high sediment yield. However, quantifying erosion rate using sediment yield is difficult because it either requires constant monitoring of sediment fluxes or sediment deposits that have captured all sediment generated in a specific area [29]. Over the last several decades, the use of terrestrial cosmogenic nuclides, particularly ^10^Be, has provided unprecedented insight into quantification of erosion rate over millennial timescales (10^2^ to 10^5^ years), giving critical insight into factors influencing erosion rate [1, 30–37].

Efforts to quantify erosion rate using ^10^Be broadly fall into two categories: local to regional scale studies that generally aim to determine the effects of key variables in a specific region, and global compilations of data from various parts of the globe that seek to understand which variables are most important across different regions. These ^10^Be-based studies suggest that a multitude of variables influence erosion rate, including: channel steepness [38], mean basin slope [32, 33, 39–41], vegetation [9, 39, 42], elevation [32], relief [32], temperature and mean annual precipitation [10, 12, 43, 44–47], variability in precipitation [39], and tectonic uplift [1, 48, 49–51].

Many ^10^Be studies, both local and global, have recognized mean basin slope to be significantly correlated with erosion rate [1, 32, 33, 39, 40, 41]. This relationship between slope and erosion rate is unsurprising as steeper slopes have more gravitational energy, which facilitates the movement of sediments. Some studies suggest that erosion rate increases with steepness in slope only up to a threshold value [52, 53], whereas others suggest in areas with high uplift rate, relief correlates best with erosion rate [51, 54]. However, overall slope is observed as the dominant variable influencing erosion rate in previous global ^10^Be compilations [32, 33].

### Precipitation’s influence on erosion rate

Intuitively, precipitation should have a strong influence on erosion rate, as water facilitates the weathering process that precedes erosion in most environmental settings and is also one of the main agents for transport of sediments [21, 24, 25]. Indeed, many studies assert that rainfall is the primary cause of erosion in many environmental settings [2, 24, 25, 55, 56]. In contrast, many ^10^Be-based studies on the correlation between various environmental variables and erosion rate suggest a small to nonexistent relationship between precipitation and erosion rate [1, 32, 33, 40, 49, 50, 57–60].

Local to regional-scale ^10^Be studies suggest that higher precipitation results in faster erosion in some regions, but not in others. For example, [61] suggest that the oldest surfaces on Earth are found in hyper-arid areas, such as the Negev and Atacama Deserts; and [44] also observed an increase in erosion rate with an increase in precipitation across all of Australia. In contrast, this relationship between increased aridity and slow erosion is not observed in the semi-arid Namib Desert [62] or across semi-arid Australia [63]. In the case of the Namibian margin, it is suggested that the entire region had attained steady-state and has been eroding at similar rates over the past 36 Million years, making climatic influences less significant [62]. In similarly contrasting findings, [43] found that the combined influence of temperature and precipitation do influence erosion rate in the western Sierra Nevada Mountains, California, USA; however, [50] notes only a weak climatic control on the erosion rates across the Sierra Nevada. In another example, [64] notes strong coupling between precipitation and long-term erosion rate in a mountainous setting (Washington Cascades); but, [54] finds no influence of precipitation on erosion rate in the Himalayas.

This contrast between the role of climatic (e.g. precipitation and temperature) verses tectonically linked variables (e.g. slope, channel steepness, and uplift rate) observed in various ^10^Be studies could be partly related to the landscape obtaining steady-state [11, 65]. For instance, a landscape that has reached a steady-state with respect to external factors will have its erosion rate controlled by the uplift rate that provides material for erosion [1, 65]. Conversely, if the landscape has not yet reached steady-state and is in transition phase, then factors such as precipitation, vegetation, and temperature will control erosion rate [65].

The complex interrelationship between these different factors is also evident in sediment yield studies. Some sediment yield studies suggest precipitation is extremely important to erosion and that there is a strong correlation between erosion and precipitation and/or vegetation [21, 22, 66, 67–71]. For instance, [21] found that sediment yield increases with an increase in precipitation, reaching a maximum at approximately 254–355 mm/yr; beyond this sediment yield decreases when precipitation increases further. This decrease in sediment yield beyond 355 mm/yr of precipitation is attributed to an increase in vegetation density [21]. [69] observed that sediment yield reaches a maximum when climate transitions from an arid environment into a semi-arid or humid environment. However, some studies emphasize the role of relief in controlling variability in sediment yield on a global scale [28, 72, 73]. For instance, [28] analyzed sediment yield data from 33 major rivers and found that basin relief provides the best statistical explanation for variation in sediment yield. Similarly, [74] found that sediment yields from “mountain” rivers were three times higher than “plains” rivers, but within the two groups, sediment yield varied with climate.

Despite the seemingly obvious link between erosion and precipitation, ^10^Be-based global compilations [32, 33, 38] do not show any statistically significant numerical relationship between long-term erosion and precipitation. Instead, [32], in their global compilation concluded that mean annual precipitation might be important on the local scale, but is non-significant at global scale. Similarly, the percentage of vegetation cover is non-significant at influencing ^10^Be-derived erosion rates [32].

One reason for the lack of a statistically meaningful correlation between precipitation and erosion rate in global compilations could be that other variables, such as slope, lithology, relief, or rock uplift rate, have greater significance [32, 33, 51] and obscure the correlation with precipitation. For example, [51] found that tectonic setting and the rate of rock uplift determines if precipitation or relief will have the strongest influence on erosion rate. Another possibility is that intrinsic characteristics of the precipitation regime, such as precipitation variability [39, 65, 75], magnitude of maximum precipitation events [76] and the relationship between precipitation and vegetation [9, 71, 77] might be more important than mean annual precipitation itself. In particular, the evidence from sediment yield studies clearly suggest that vegetation may obscure any potential impact that precipitation has on erosion rate because high rainfall increases erosive power but results in dense vegetation that holds back sediments [9, 21, 22, 25, 66, 67, 69, 70, 77].

### Aim of this compilation

There exists a contrast between the intuitive link between precipitation and erosion and comparisons of ^10^Be results and precipitation. In this compilation, we examine precipitation and ^10^Be-derived erosion rate in a global context that acknowledges the important influence of other variables, particularly slope, which emerges as the dominant variable in previous ^10^Be compilations, and vegetation, which may complicate the correlation between precipitation and erosion by interacting with both erosion and precipitation.

## Methods

### Erosion rates compilation and standardization

Terrestrial or in-situ ^10^Be is a radioactive isotope of beryllium with a half-life of 1.39 My (1.39 x 10^6^ years) [78, 79] and is formed by cosmic-ray spallation of an oxygen atom [80]. The concentration of ^10^Be in quartz-bearing rocks is inversely proportional to the erosion rate of these rocks [30, 31, 35, 37, 81] because more ^10^Be is produced during longer exposure to secondary cosmic rays as a result of slower erosion. Similarly, where erosion is fast, exposure to secondary cosmic rays is shorter, resulting in a lower concentration of ^10^Be nuclides [30, 31, 35, 37, 81]. Erosion rates derived from ^10^Be are generally averaged over the past several thousand years and are calculated based on the assumption that the rock is eroding at a constant rate, and that the concentration of ^10^Be is at steady state [30, 31, 35, 37, 81]. When sediment is collected for ^10^Be-derived erosion rates, the result is generally considered to be the basin-averaged erosion rate. The primary assumptions for calculating basin-averaged erosion rates are: (1) all lithologies in the catchment are eroding at the same rate; (2) all rock types contributing to erosion have similar grain sizes; (3) there is minimal time spent in sediment storage; and (4) the timescale of erosion is smaller than the timescale of radioactive decay of ^10^Be [30, 31, 35, 37, 81].

The timescale of ^10^Be-derived erosion rates is calculated by dividing the erosion rate by the absorption depth of secondary cosmic rays [1]. An erosion rate of 1000 m/My is averaged over a timescale of 600 years; in contrast, an erosion rate of 10 m/My is averaged over 60 ky. For this reason, ^10^Be-derived erosion rates are often considered millennial-scale erosion rates [30, 31, 35, 37, 81]. Some studies quantifies erosion rate over short-term, which is usually calculated from contemporary sediment yields [28], and is averaged over few years or decades. Short-term erosion rates represent a combination of both natural and anthropogenic-induced erosion [28, 42] and are potentially subject to a degree of uncertainty, primarily because of the episodic nature of sediment delivery [28, 82]. For example, [83] found in central Idaho that long-term erosion rates were on average seven times higher than the short-term erosion rate and suggested that this is because the sediment delivery is episodic in the mountainous terrain [83]. Similarly, in central Europe, [84] found that long-term erosion rates are 1.5–10 times higher than short-term erosion rates because short-term erosion rate underestimate the amount of sediment generated. Long-term erosion rate on contrary integrates erosion rate over millennial timescale and therefore includes the entire range of episodic discharges and loads [84].

We recognize that the timescale over which ^10^Be-derived erosion rates are determined is dependent on the rate of erosion itself, and is generally longer than the timescale over which variables, such as precipitation and vegetation cover are determined. However, although not constant, paleo-precipitation and paleo-temperature over the past several thousand years exhibit broadly similar gradients [85]. Furthermore, global climate zones over the timescale of ^10^Be-derived erosion rates are not dramatically different from today [85]. Thus, we compared ^10^Be-derived erosion rates with modern precipitation and vegetation cover following a similar approach to previous global compilations, such as [32] and [38]. Comparing modern vegetation cover with long-term erosion rate is imperfect, but it does broadly allow exploration of the influence of vegetation cover, if any, on long-term erosion rate on global scale.

**Fig 1:**
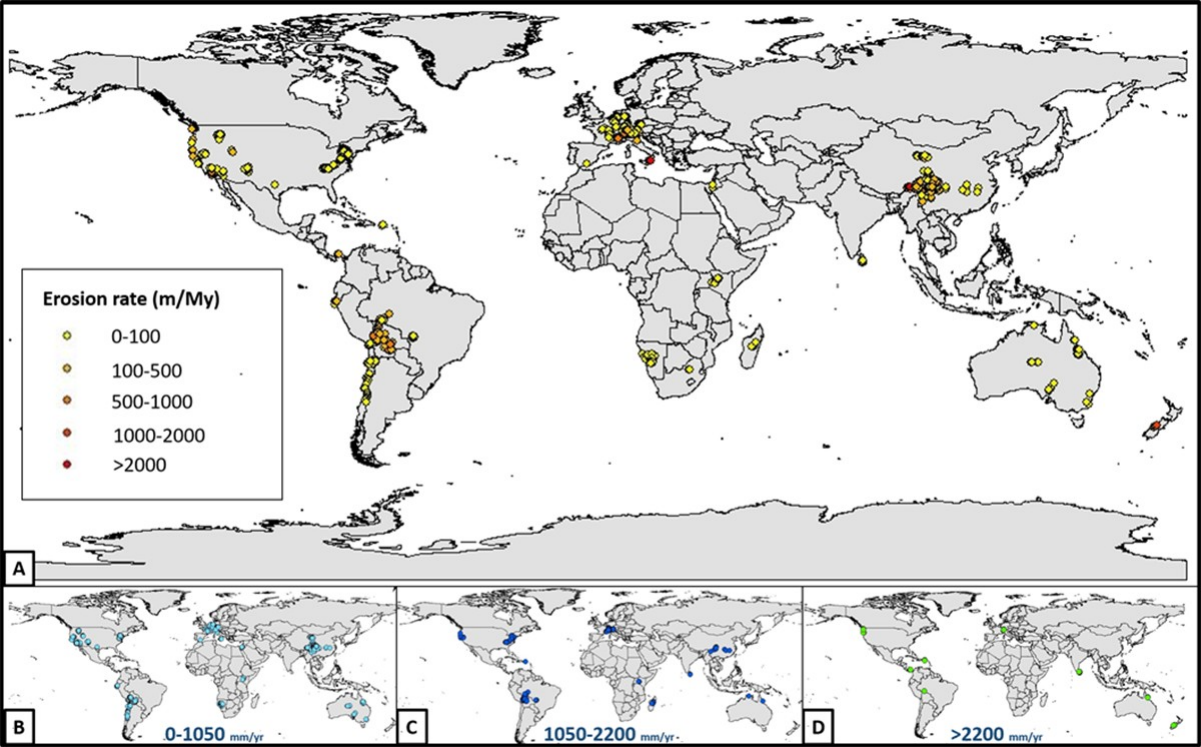
(A.) Geographic distribution of basin averaged ^10^Be-derived erosions rate samples (refer to S1 File for data and the list of source publications). (B.) Samples with mean annual precipitation between 0–1050 mm/yr. (C.) Samples with mean annual precipitation between 1050–2200 mm/yr. (D.) Samples with mean annual precipitation >2200 mm/yr.

Here, we have compiled data (n = 1790) from 93 published studies that use the concentration of ^10^Be in sediment samples to determine millennial-scale erosion rates (Fig 1). The scope of our compilation is similar to that of [32], [33], and [38]. The data was collected from various studies and compilations published prior to 2016, so erosion rates were recalculated based on the new update of CRONUS (Version 2.3) [86](hess.ess.washington.edu). We used [31] and [87] scaling scheme for our entire dataset. Erosion rates from Antarctica are excluded in this compilation.

### Mean basin slope, mean annual precipitation, and tree cover

‘Mean basin slope’ for this compilation was retrieved using DEM with ArcGIS, using ~3 arc-seconds (90m) SRTM data (http://srtm.csi.cgiar.org), ensuring that all mean basin slope values in our compilation were calculated using similar DEM and SRTM resolution. Mean annual precipitation values for the entire dataset were retrieved using available data set from [88]. Tree cover data was retrieved from an existing dataset using 1-km resolution by [89]. Following similar approaches of [32] and [38], 0–10% values in the data set were replaced with 5% and non-vegetated areas were replaced with 0%. To standardize our data, all parameters were retrieved using similar method, and erosion rates were re-calculated based on the latest updated version of CRONUS.

### Statistics

All statistical analyses used R (R Core Team, 2016). Univariate and multiple regressions and R^2^ estimates used the base statistics package. Mixed-effects models used the nlme and lme4 packages, with pseudo-R^2^ values provided by the MuMIn package. We performed all statistical analyses assessing significance at the 95% confidence level; therefore, p-values > 0.05 are not statistically significant [90].

To check for secondary influences of mean annual precipitation and tree cover on erosion rate, we used multiple regression models and mixed effect models. The main objective is to examine if the model between response variable (in this case erosion rate) and the predicting variable improves by adding another variable to the model. Thus, if adding a particular parameter improves the explanation of the variances (R^2^ value), then that parameter is of secondary statistical significance.

We undertook three sets of analyses. The first used unadjusted univariate linear and polynomial regressions to evaluate the overall relationship of each of three individual explanatory variables (mean basin slope, precipitation and tree-cover percentage) with the log-transformed erosion rate. The second set of analyses included all three of these explanatory variables in the same model. These two sets of analyses treat every available data point as independent and do not consider correlation between erosion rates observed within different studies. The mixed-effects analyses described below, which allowed for intra-study correlation, indicated that greater complexity was not justified, so we restricted the polynomial fits to linear, quadratic or cubic regressions. Inflection points for the tree-cover and precipitation curves were also identified.

Within individual studies, estimates of erosion rate tended to be very similar, indicating that individual measurements within a study could not be regarded as completely independent, presumably because they shared a range of other attributes (e.g. rock type, tectonic setting) with local influences. The third set of analyses, therefore, used an additive, mixed-effects random intercept model with citation (the study providing the data) as a random effect, and with the fixed effects listed below:

- Mean basin slope
- Tree cover percentage (as a quadratic polynomial)
- Precipitation (as a cubic polynomial)

Aikake Information Criterion (AIC) and Analysis of variance (ANOVA) comparisons identified the level of polynomial used for each explanatory variable. All analyses are attached as supplementary materials.

## Results

Erosion rates determined using CRONUS 2.3 range from 0.07 m/My to 4119.53 m/My. The slowest erosion rates are observed in the driest regions of the Atacama Desert, Chile (Slope = 2.4°; Precipitation = 3 mm/yr) [41, 91], whereas the fastest is observed in Namche Barwa-Gyala Peri Massif, Tibet (Slope = 21.6°; Precipitation = 559 mm/yr)[92].

### Primary influence

**Fig 2:**
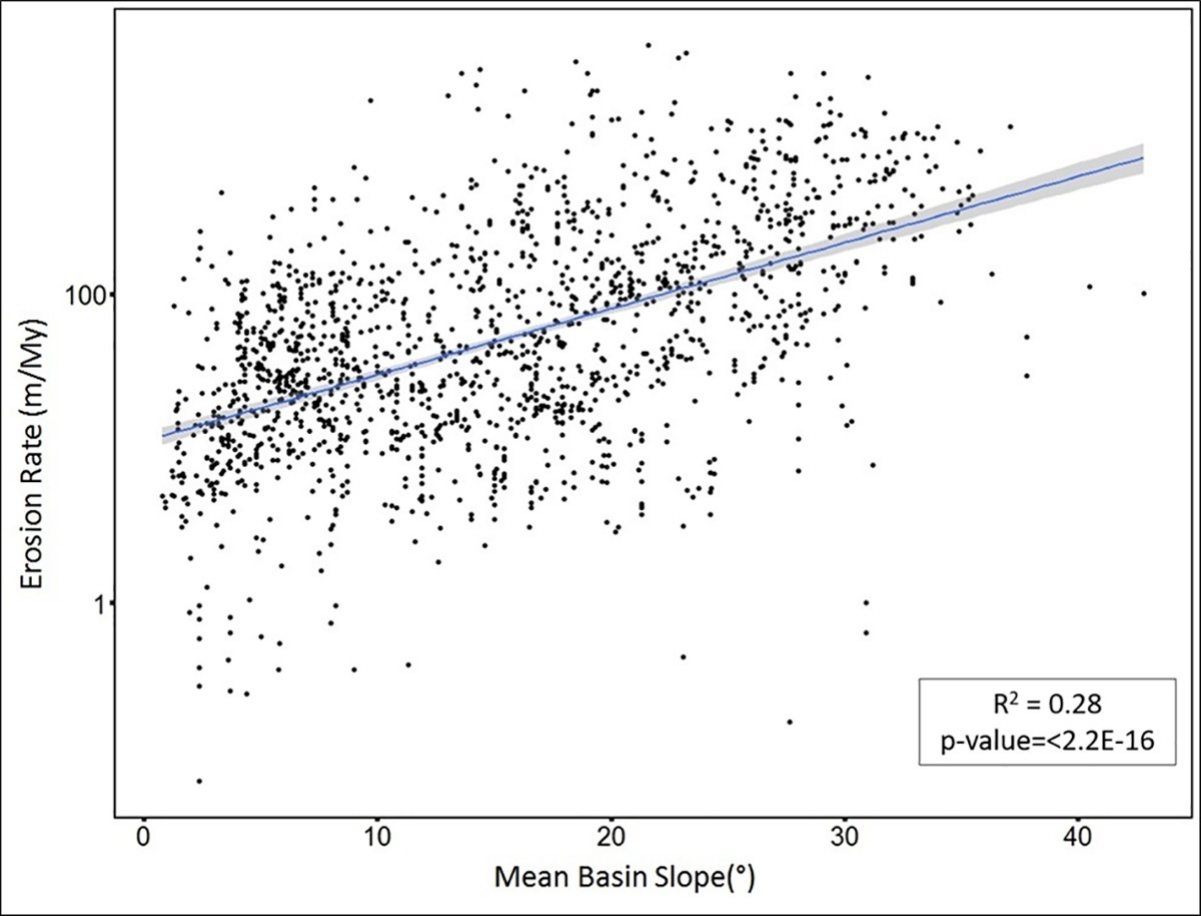
Mean basin slope (x-axis) versus log erosion rate (y-axis). The blue line indicates the linear relationship best fit line between erosion rate and mean basin slope. The grey area around the blue line represents confidence interval.

Results of the correlation of variables with ^10^Be-derived erosion rate is expressed according to their R^2^ values in Table 1. On a global scale, a significant positive, but moderate correlation is observed between rate of erosion and mean basin slope, with an R^2^ value of 0.28 and p-value <2.2e-16 (Fig 2). There is no evidence of curvature in this relationship. For all areas where slope is <11°, correlation of mean basin slope with erosion rate weakens to R^2^ = 0.08, p-value = 2.4e-13.

**Table 1:** Variables with their R^2^ value using erosion rate as response variable.

Predictors/Variables	R^2^ Value of this compilation	P-value	R^2^ value from Portenga and Bierman (2011)	R^2^ values from Willenbring et al., 2013)	R^2^ values from Harel et al., (2016) (used channel steepness index)
Slope	0.284	<2.2e-16	0.346	0.48	_
Precipitation	0.017	0.0000001	0.008	_	-0.003
Precipitation (polynomial)	0.049	<2.2e-16	_	_	_
Vegetation	0.006	0.0006457	0.028	_	-0.034
Vegetation (polynomial)	0.124	<2.2e-16	_	_	_

**Fig 3:**
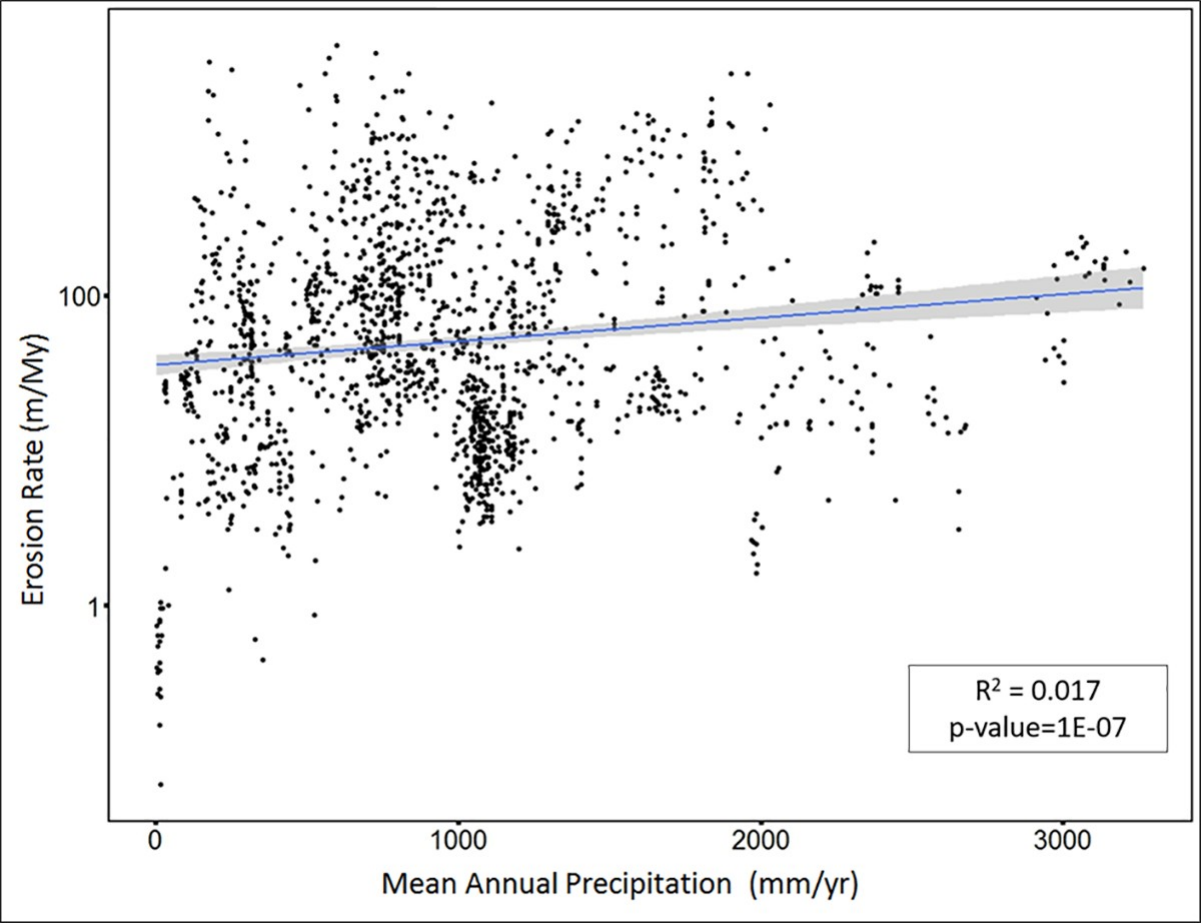
Linear regression: Mean annual precipitation (x-axis) versus log erosion rate (y-axis). Blue line indicates linear relationship best fit line between erosion rate and mean annual precipitation. The grey area around the blue line represents confidence interval.

**Fig 4:**
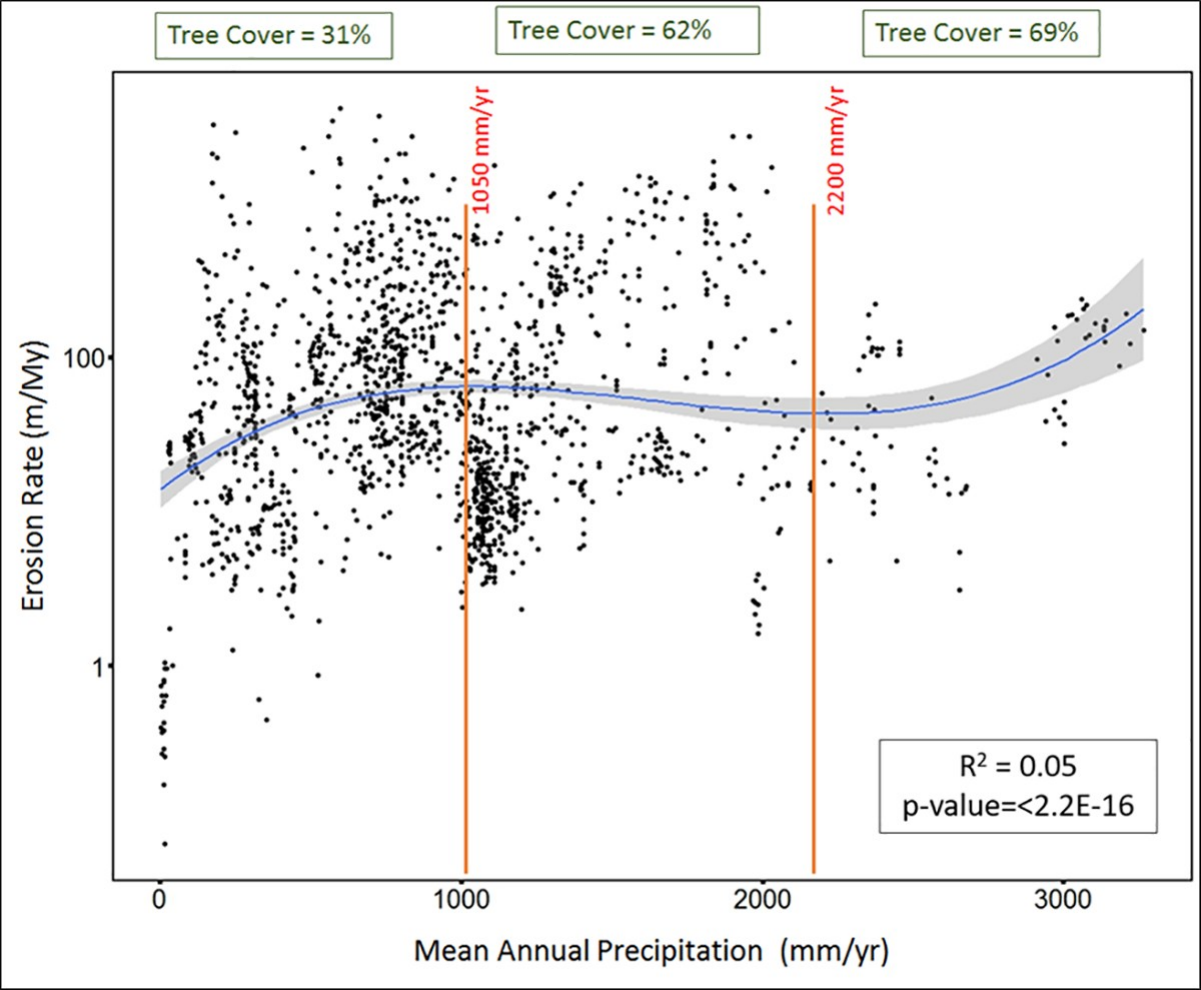
Non-linear regression: Mean annual precipitation (x-axis) versus log erosion rate (y-axis). Blue line indicates the non-linear relationship curve between erosion rate and mean annual precipitation. The grey area around the blue line represents confidence interval. Orange vertical line represents inflection points, where the relationship curve trend changes. Green box on top represents average tree cover percentage in each regime.

Mean annual precipitation shows a very weakly significant primary linear correlation with erosion rate (R^2^ = 0.017 and p-value = 0.000001; Fig 3). However, the data and its distribution indicates a non-linear relationship. The polynomial correlation between erosion rate and mean annual precipitation has R^2^ value of 0.05, and p-value <2.2e-16 (Fig 4). The relationship is best described by a 3^rd^ order polynomial model according to the AIC values (See S2 File). The AIC values for each model was very close, which might be due to the variability within a study and the variability between studies with similar values of the explanatory variables. Therefore, we analyzed the AIC values for models using single data points per study (i.e. by calculating average values for each study for both the explanatory variables and log erosion rates). The analysis of AIC values for order 3 and above in the mixed effects model are very close, and the higher-order polynomials do not retain their advantage when each study is allowed to contribute only a single data point. Therefore, we decided to take the most conservative option and use the 3rd-order polynomial. Inflection points denoting the maxima and minima of the best fit curve are points where the fitted relationship between erosion rate and precipitation changes its trend. In this case, erosion rate continues to increase with precipitation until the precipitation value reaches ~1050 mm/yr (first inflection point). From there, the relationship between erosion rate and precipitation is inverse until the precipitation value of ~2200 mm/yr (second inflection point), after which erosion rate again increases with further increase in precipitation.

**Fig 5:**
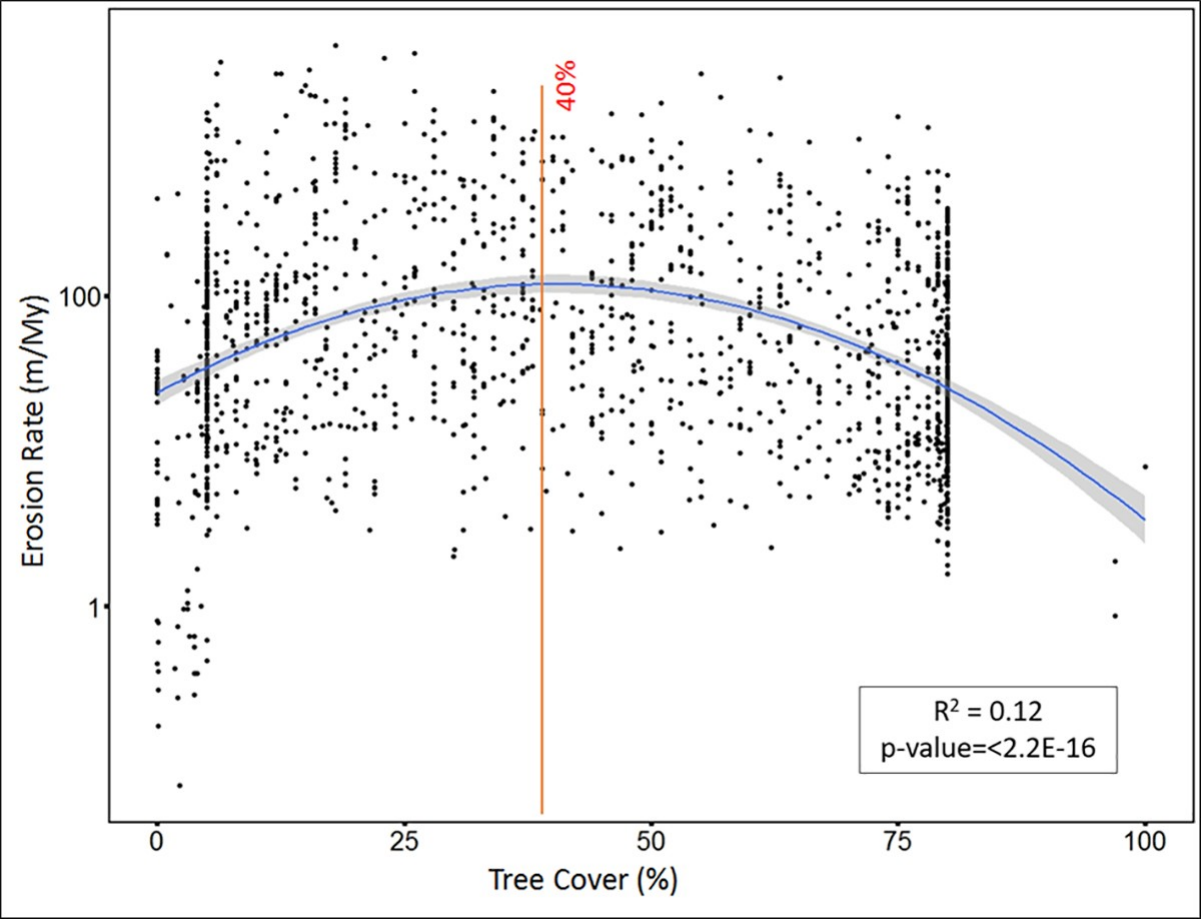
Non-linear regression: Percentage of tree cover (x-axis) versus log erosion rate (y-axis). Blue curve line indicates non-linear relationship curve between erosion rate and tree cover percentage. Orange vertical line indicates the maxima or threshold value of tree cover percentage.

**Fig 6:**
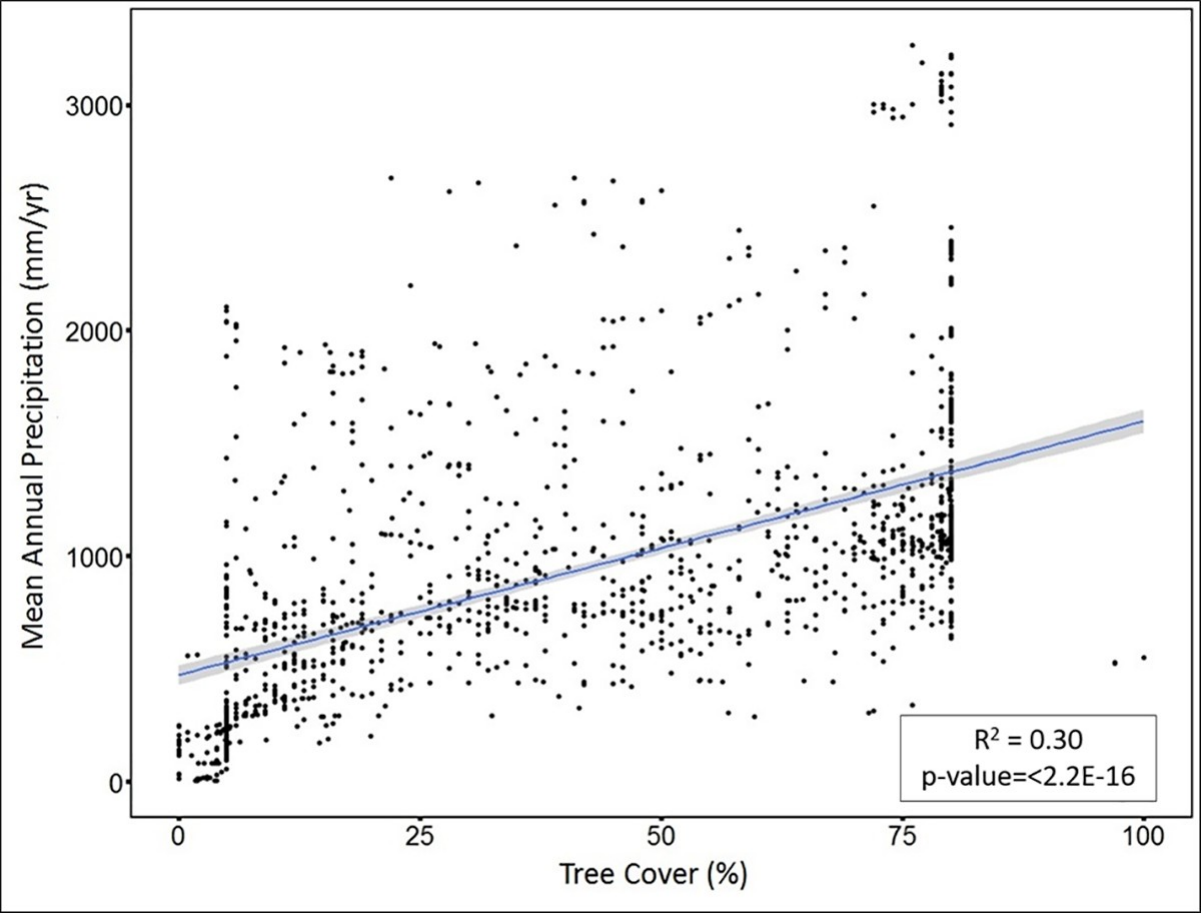
Linear regression: Percentage of tree cover (x-axis) versus mean annual precipitation (y-axis).

The percentage of tree cover also shows a weak linear correlation with erosion rate, with R^2^ value of 0.006 and p-value = 0.0006457. Allowing for curvature via a quadratic fit increases the R^2^ value to 0.12, p-value <2.2e-16 (Fig 5). The inflection point of the relationship implies that the maxima is at 40% tree cover. The percentage of tree cover is linearly correlated with precipitation, with R^2^ value of 0.30 and p-value < 2.2e-16 (Fig 6).

The R^2^ values here indicates the variation in response variable (in this case erosion rate) explained by the model. The R^2^ values reported are extremely low because data was compiled from different environmental settings that are widely scattered. However, with such a large dataset that consists of wide range of variables, having low R^2^ value to express correlation is not unusual.

### Secondary influence of precipitation and percentage of tree cover:

The relationship between precipitation and percentage of tree cover has a slightly better correlation with erosion rate when curvature is allowed (R^2^ value increasing from 0.01 to 0.05 for precipitation, and from 0.006 to 0.13 for vegetation). However, when the percentage of tree cover and precipitation both were added to the model, the combined correlation between them and erosion rate increased the R^2^ value to 0.18 (p-value <2.2e-16), suggesting that precipitation and tree cover together better explains the variation in erosion rate data (See S2 File). However, in the combined model, precipitation and vegetation have opposite effects on rate of erosion (see estimated regression coefficients in S2 File), suggesting both mechanistically and statistically, precipitation and tree cover act in different directions in terms of their influence on erosion.

Of the compared variables, mean basin slope has the most dominant correlation with erosion rate (R^2^ value of 0.28 and p-value <2.2e-16). Adding precipitation and tree cover to the model between erosion rate and mean basin slope increases the R^2^ value to 0.40 and the p-value is <2.2e-16. This indicates that the explanation of the variances in the erosion rate data is improved when mean basin slope, precipitation and tree cover are all considered.

We included study identifier (i.e. citation) as a random effect in the mixed effect model. This improved the explanatory power of the model substantially (R^2^ = 0.81 and p-value 0.0015). In order to check how much of this explanatory power is improved by mean annual precipitation alone, we considered another mixed effect model, without precipitation, and compared the R^2^ values. Our comparisons suggests that precipitation improves the explanation of variance in the model by ~40% (fixed effect R^2^ value improves from 0.138 to 0.194) (Table 2).

**Table 2:** Statistical models and their R^2^ value and p value.

Model	R^2^ value	p-value
Erosion rate ~ Precipitation(poly)	0.05	<2.2E-16
Erosion rate ~ Tree Cover (poly)	0.12	<2.2E-16
Erosion rate ~ Precipitation(poly) + Tree cover(poly)	0.18	<2.2E-16
Erosion rate ~ Slope	0.28	<2.2E-16
Erosion rate ~ Slope + Precipitation(poly) + Tree Cover(poly)	0.4	<2.2E-16
Erosion rate ~ Slope + Precipitation(poly) + Tree Cover(poly) (Random effect = Citation)	0.81	0.0015
Erosion rate ~ Slope (for all areas with <11° slope)	0.08	2.48E-15
Erosion rate ~ Precipitation(poly) (for all areas with <11° slope)	0.08	2.98E-13

**Fig 7:**
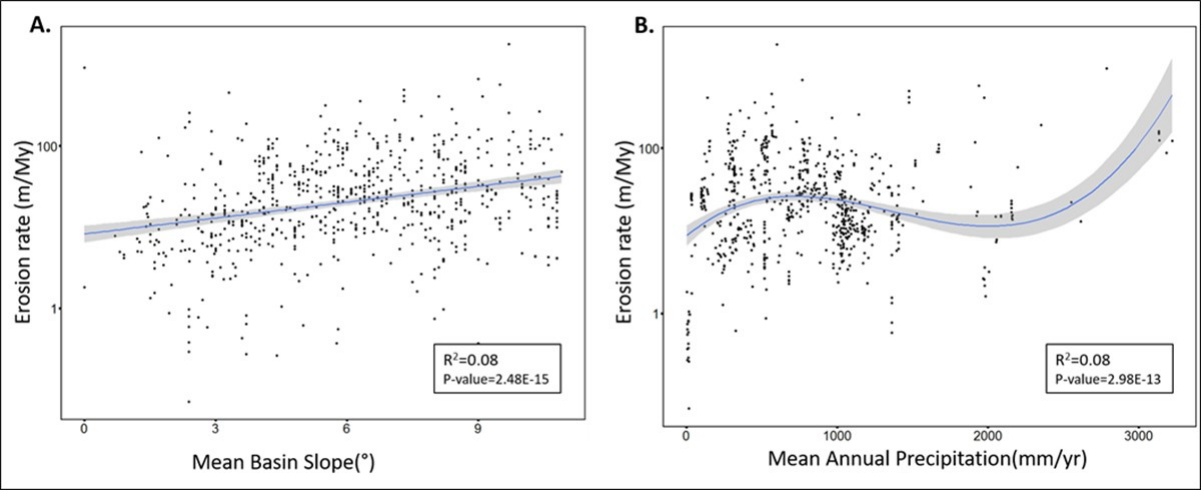
Plots of mean basin slope and precipitation with erosion rate in areas with <11° slope. A. Mean basin slope (x-axis) versus log erosion rate (y-axis). B. mean annual precipitation (x-axis) versus log erosion rate (y-axis).

We also examined the correlation of both slope and precipitation with erosion rate for areas with slope <11° and found that mean annual precipitation has a similar correlation (R^2^ = 0.08, p-value = 2.98e-13) as mean basin slope (R^2^ = 0.08, p-value = 2.48e-15) (Fig 7). Correspondingly, we also found that the correlation between mean annual precipitation and erosion rate is better (R^2^ = 0.1717, p-value = <2.2e-16) in areas where elevation is <1000m and slope is <11° (Table 3). In addition to that, the response of erosion rate to change in mean annual precipitation was also examined for different lithologies (S1 Fig). The correlation between mean annual precipitation and erosion rate was found to be highest for ‘mixed’ lithologies (R^2^ = 0.1063, p-value = 2.89e-15). However, in areas with elevation <1000m, the correlation between mean annual precipitation and erosion rate was highest when the lithology was ‘igneous’ (R^2^ = 0.6681, p-value = <2.2e-16; Table 3).

**Table 3:** R^2^ value and p-value of erosion rate vs. mean annual precipitation when other variables are constant.

Condition	All Data		Elevation>1000m		Elevation<1000m	
Erosion vs. Precipitation in areas where:	R^2^ value	p-value	R^2^ value	p-value	R^2^ value	p-value
Igneous lithology	0.0019	0.299	-0.00952	0.9084	0.6681	<2.2E-16
Metamorphic lithology	-0.0000903	0.3985	0.036	0.003906	0.077	0.0034
Mixed lithology	0.1063	2.89E-15	0.315	<2.2E-16	0.009	0.1408
Sedimentary lithology	0.0052	0.1873	0.1515	0.00021	0.005	0.2363
Slope <11°	0.05	<2.2E-16	0.0143	0.06079	0.1717	<2.2E-16
Slope>11°	-0.0017	0.7784	0.0089	0.0255	0.024	0.007541

## Discussion

Our results suggest a relationship between mean basin slope and erosion rate that is similar to that observed in previous studies and compilations [32, 33, 39–41]. Observationally, sites (e.g. Atacama, Namibian Desert and Escarpment, Sechura Desert) in hyper-arid areas have average basin slopes (11°, 6.1°, and 0.8°, respectively) that are lower than the average slopes for the entire dataset (15.1°) (refer S1 File) and sites with higher mean annual precipitation tend to have higher mean basin slope (e.g. Southern Alps in New Zealand—31°, Tibetan Plateau—16.08°, and Sri Lankan escarpment—28.6°) (refer to S1 File). However, mean basin slope and mean annual precipitation do not show any direct significant statistical correlation (R^2^ = 0.08, p-value = 2.48e-13) (S2 Fig).

### Precipitation’s influence

Across the entire data set, mean annual precipitation has a very weak correlation with erosion rate when compared linearly and directly (Fig 3). However, the relationship between erosion rate and precipitation is somewhat better (R^2^ value 0.05) when polynomial fit is allowed (Fig 4). The correlation remains weak, indicating that noise created by other variables significantly influences erosion rate. However, including precipitation in a statistical model of erosion rate with mean basin slope gives a significantly improved model. Absence of precipitation from the model decreases the explanatory power of fixed effects by ~40%, which again indicates that the optimum model for factors influencing erosion rate must include precipitation. The large variation attributable to individual studies suggests that other locally specific environmental attributes, such as lithology, uplift rate, or relief, also have a major influence on erosion rate. This response of erosion rate to precipitation is valid only for silicate terrains, and carbonate terrains might have different response, as rate and characteristics of weathering of carbonate terrains is different and are beyond the scope of this study [93, 94].

In areas with slope less than 11°, which includes more than 92% of Earth’s surface [33], mean annual precipitation correlates with erosion rate (R^2^ value 0.08) as much as mean basin slope (R^2^ value 0.08). This correlation between mean annual precipitation and erosion rate further improves in areas where slope <11° and elevation is <1000m (R^2^ value 0.1717) (Table 3). This implies that when topographic influence is low (i.e. when elevation and slope are low), the correlation between mean annual precipitation and erosion rate improves. Lithology also plays an important role in this correlation. Mixed lithology favors a stronger influence of mean annual precipitation on erosion rate in areas where elevation is >1000m (R^2^ value 0.315). However, in areas where elevation is <1000m, igneous lithology strengthens precipitation’s influence on erosion rate (R^2^ value 0.6681).

The non-linear relationship between erosion rate and mean annual precipitation suggests that erosion rate responds to change in mean annual precipitation in three different regimes. First, it tends to increase with an increase in mean annual precipitation until ~1000 mm/yr, after which it starts decreasing with an increase in mean annual precipitation until ~2200 mm/yr. At greater than ~2200 mm/yr erosion rate again starts increasing with further increase in mean annual precipitation (Fig 4). This complex trend also potentially lowers the overall statistical correlation of precipitation with erosion rate.

The percentage of tree cover correlates with erosion rate non-linearly, and the threshold (maxima) value of this correlation is 40%. In other words, once tree cover reaches more than 40%, it starts to counteract precipitation’s influence on erosion rate, thereby slowing down erosion rate. This is consistent with several simulation studies [95, 96, 97] that found that vegetation fails to slow down erosion rate significantly until vegetation cover reaches a threshold value. This complex interaction between tree cover and erosion rate, and a threshold value for tree cover acting against precipitation’s influence on erosion rate, explains the three regimes of mean annual precipitation’s influences on erosion rate:

- **First regimes:** In the first regime, erosion rate increases with increased mean annual precipitation (from 0–1050 mm/yr). Over this regime, the mean value of tree cover is 31%. Although tree cover increases with increasing precipitation, the influence of tree cover is not enough to counteract the erosive ability of higher rainfall, and therefore erosion rate increases with increased mean annual precipitation. This observation is consistent with observations made in previous studies that indicated slow erosion in arid and hyper arid environments [41, 61, 91]. However, it is also noteworthy that vegetation does not always acts against the erosive ability of precipitation. In some settings, such as in transition from arid to semi-arid environment, vegetation facilitates erosion by promoting weathering in the root zone due to high pCO_2_ [98].
- **Second regime:** The second regime (1050–2200 mm/yr) is characterized by erosion rates that do not increase with an increase in mean annual precipitation. The mean value of tree cover in this regime is ~62%. In this regime, the combined effect of mean annual precipitation and tree cover results in decreasing erosion rate with further increase in precipitation. Although an increase in precipitation tends to increase erosion rate, the response of vegetation to higher rainfall neutralizes precipitation’s influence and ultimately stabilizes and lowers the erosion rate. A similar response has been noted in sediment yield studies [21, 69, 70], where the response of vegetation lowers sediment yield despite increasing precipitation.
- **Third regime:** The third regime (>2200 mm/yr) is characterized by erosion rates increasing with increased mean annual precipitation, and is quite complex. In this regime the mean value of tree cover is ~69%. In spite of the tree cover percentage being greater than the threshold value, erosion rate in this regime tends to increase with an increase in precipitation. This is likely due to the mechanism of how trees lower soil erosion against high intensity of rainfall. Trees primarily control soil erosion in two ways: first, roots hold the soil together; and second, leaf litter accumulates on the topsoil and protects it from eroding [99–101]. Areas where the percentage of tree cover and precipitation are both high are often rich in nutrients [99, 100]. This abundance of nutrients means that the roots don’t spread laterally and leaf litter decomposes faster [99, 100] resulting in a lower protection of soil against erosion. High tree cover percentage often also means a thick canopy, which ultimately restricts under-story growth, resulting in less protection against erosion [102]. Furthermore, areas with high mean annual precipitation experience a higher frequency of landslides, resulting in a high erosion rate [76]. The influence of relief is also apparent in this high rainfall regime. For instance, sites from Sri Lanka [42] with average slope of ~13.3° and average precipitation of ~2610 mm/yr yielded an average erosion rate of ~18m/My. Conversely, sites from Swiss Alps [103] with average slope ~26.6° and average precipitation of ~2450mm/yr recorded an average erosion rate of ~217m/My. Thus, the erosion rates from Swiss Alps are ~10 times higher than average erosion rate observed from Sri Lankan sites, although both sites experience almost same average precipitation. This further implies that areas with lower relief, but very high precipitation will have lower erosion rate, despite the fact that high precipitation acts toward increasing erosion rate.

**Fig 8:**
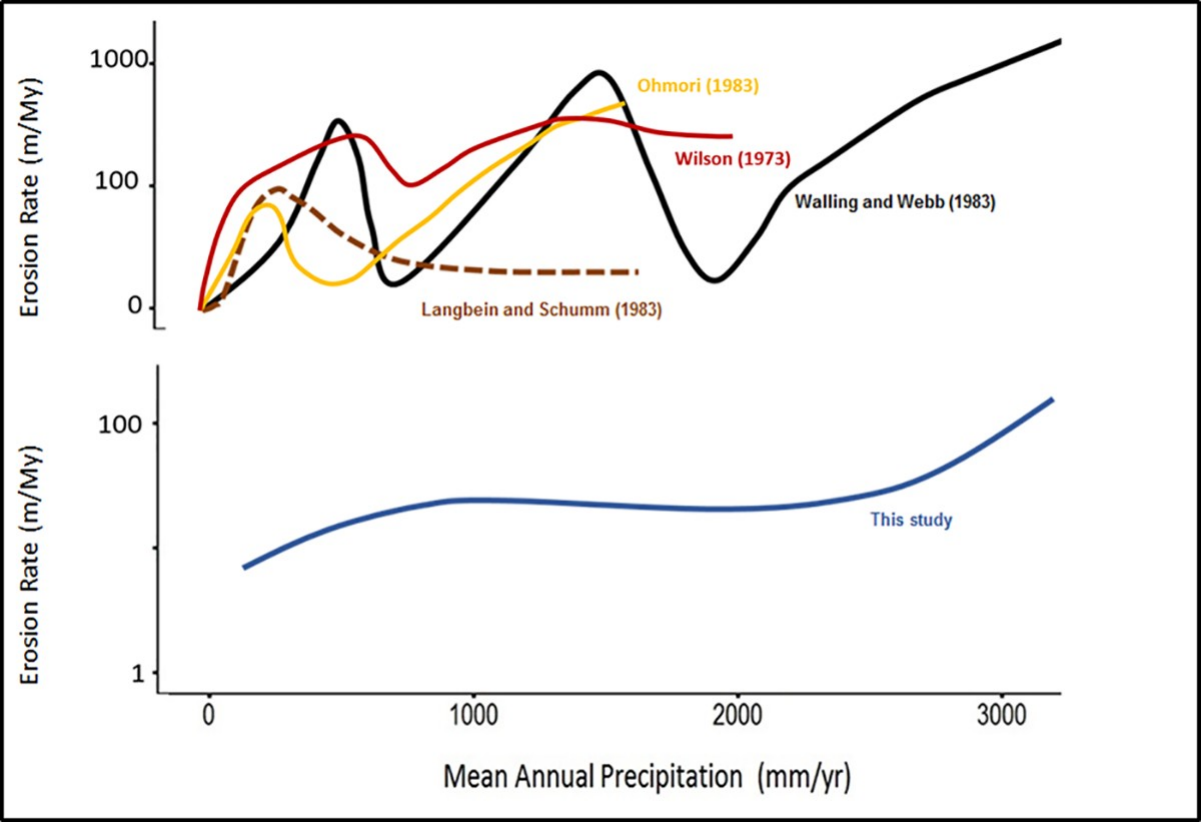
Comparison of the relationship between erosion rate and mean annual precipitation from different studies. Black solid curve represents the relationship between precipitation and erosion rate from Wailing and Webb (1983), Brown dashed curve represents the relationship from Langbein and Schumm (1958), yellow solid curve represents relationship from Ohmori (1983), Red solid curve represents the relationship from Wilson (1973), and the blue solid curve represents the curve from this study. Wailing and Webb (1983) and Langbein and Schumm (1958) originally depicted the relationship between sediment yield and precipitation; sediment yield (t km^-2^yr^-1^) is converted to m/My using 1 m/My = 2.7 t km^-2^yr^-1^.

This three-regime relationship accounts for the response of erosion rate to both change in precipitation and the co-related change in tree cover percentage. This non-linear relationship between erosion rate and mean annual precipitation also suggests similarity to the curve observed by previous sediment yield studies [21, 69, 70](Fig 8). Although, not entirely comparable, the sediment yield curves attest the non-linearity of the relationship between precipitation and erosion rate. This observation is also consistent with the findings of [71] in Chilean Coastal Cordillera, where denudation rates increased with increase in mean annual precipitation until ~1000 +/- 500 mm/yr; after this value, denudation rates did not increase with further increase in mean annual precipitation [71]. This stability in denudation rate at ~1000 mm/yr is attributed to vegetation cover, suggesting a vegetation-induced non-linear relationship between precipitation and erosion rate [71]. Our results suggests that this interrelationship operates on a global scale.

Results of our compilation highlight the critical role of vegetation and tree cover, and illustrates how tree cover is inseparable from precipitation’s influence on erosion rate. From an erosion management perspective high tree cover is widely recognized and associated with lower erosion rate [2, 9, 25, 77]. Additionally, soil production rates decline exponentially with soil depth [104] and forested landscapes are characterized by thick soils, so production of material available to erode should be less in environments where tree cover is high. However, our results suggests that at very high precipitation, erosion rate increases in-spite of high tree cover percentage.

This response of erosion rate to high mean annual precipitation and high tree cover percentage in third regime is based on limited data available from these areas. Indeed, there is an overall lack of erosion rate studies from areas with very high precipitation (>2000mm/yr). Addition of sites with very high precipitation and high tree cover percentage could potentially affect the reported trend of the third regime. For example, sites from Shillong plateau [105], where precipitation ranges between 4000 to 6000mm/yr and vegetation cover is >90%, reported average erosion rate of <100m/My. The erosion rate was higher for areas that were recently deforested [105]. Therefore, more detailed constraints are required to understand this relationship in areas where tree cover percentage and precipitation both are high. Such areas currently constitute only ~10% of our dataset and further data in such regions may further inform on this regime.

## Conclusions and implications

Although it is consistently suggested that precipitation is an important factor impacting erosion rate, global ^10^Be compilations have failed to find its significance. This may be because previous global ^10^Be compilations focused on precipitation’s primary and linear correlation with erosion rate. The relationship between mean annual precipitation and erosion rate is best explained non-linearly. From this compilation of ^10^Be-derived erosion rates, several important conclusions can be made: 1) the relationship between mean annual precipitation and erosion rate is non-linear and significant; 2) mean annual precipitation influences erosion rate in three regimes i.e. erosion rate first increases, then decreases, and then again increases with an increase in mean annual precipitation; 3) increased precipitation results in increased vegetation, causing a complex and competing influence on erosion rate that is responsible for the three regimes. These competing trends intersect at precipitation values between ~1000 and 2200 mm/yr; 4) tree cover lowers the influence of increased precipitation on erosion rate when the percentage of tree cover is 40% or more; 5) the influence of mean annual precipitation on erosion rate is often non-apparent because it is obscured by its closed coupled interaction with vegetation; and 6) in areas where slope is low (~90% of the Earth’s surface [33]), slope’s influence on erosion rate is not clear, and mean annual precipitation correlates with erosion rate as much as mean basin slope.

In the geologic record, high sediment yields are often interpreted as a result of climate change and our results suggest that high sediment fluxes occur when forests transition to grasslands/savannahs; however, over millennial timescales, aridification of grasslands or savannahs into deserts will result in lower sediment fluxes. These results also have relevance to anthropogenic influences on sediment yield, as it is widely asserted that changes in land use have increased short-term [2, 25, 42, 55] or mid-term [106] erosion. Our results suggest that vegetation loss will result in significantly higher sediment yield. In particular, deforestation in areas with high precipitation should result in very high rates of erosion.

## Supporting information

S1 Fig Plots of mean annual precipitation (x-axis) vs erosion rate (y-axis) in for each lithology. Clockwise from top-left is Igneous, Metamorphic, Sedimentary and Mixed.
(TIF)

S2 Fig Mean annual precipitation (x-axis) versus mean basin slope (y-axis). The blue line indicates the linear relationship best fit line between mean annual precipitation and mean basin slope. The grey area around the blue line represents confidence interval.
(TIF)

S1 File Supplementary dataset. (XLSX)

S2 File Supplementary statistics. (PDF)

S3 File Supplementary information and references for dataset. (PDF)
