# Supplementary figures and images for: Coupled influence of precipitation and vegetation on millennial-scale erosion rates derived from 10Be
Source: PLoS One. 2019 Jan 25;14(1):e0211325. doi: 10.1371/journal.pone.0211325 (PMC6347257; doi:10.1371/journal.pone.0211325)

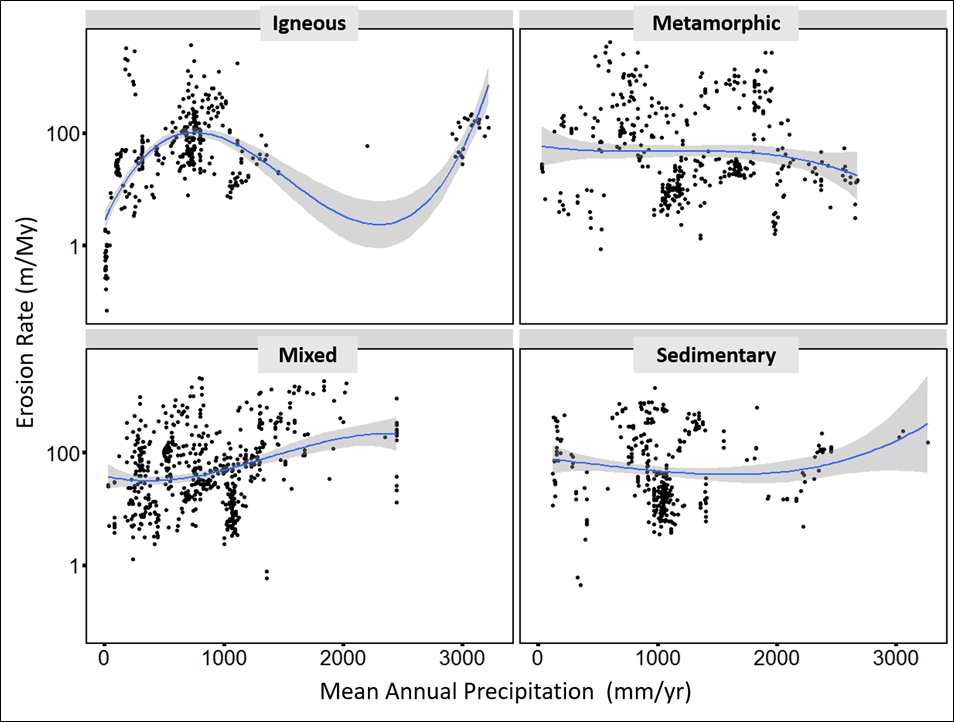

Supplement: S1 Fig — Clockwise from top-left is Igneous, Metamorphic, Sedimentary and Mixed. (TIF) [file pone.0211325.s001.tif]

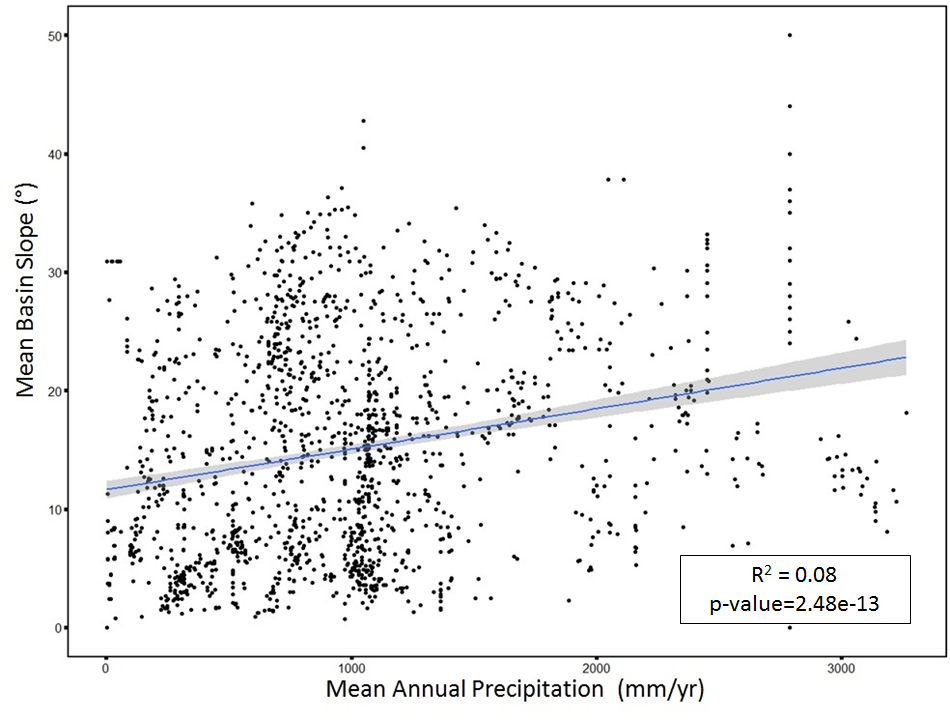

Supplement: S2 Fig — The blue line indicates the linear relationship best fit line between mean annual precipitation and mean basin slope. The grey area around the blue line represents confidence interval. (TIF) [file pone.0211325.s002.tif]
