# Supplementary material for: Coupled influence of precipitation and vegetation on millennial-scale erosion rates derived from 10Be
Source: PLoS One. 2019 Jan 25;14(1):e0211325. doi: 10.1371/journal.pone.0211325 (PMC6347257; doi:10.1371/journal.pone.0211325)
Supplement: S2 File — (PDF) [file pone.0211325.s004.pdf]

Erosion Stats

```
library(ggplot2)
```

```
## Warning: package 'ggplot2' was built under R version 3.4.4
```

```
library(lmerTest)
```

```
## Loading required package: lme4

## Warning: package 'lme4' was built under R version 3.4.4

## Loading required package: Matrix

##
## Attaching package: 'lmerTest'

## The following object is masked from 'package:lme4':
##
##     lmer

## The following object is masked from 'package:stats':
##
##     step
```

```
library(lme4)
library(dplyr)
```

```
##
## Attaching package: 'dplyr'

## The following objects are masked from 'package:stats':
##
##     filter, lag

## The following objects are masked from 'package:base':
##
##     intersect, setdiff, setequal, union
```

```
b<-theme(

   panel.grid.major=element_blank(),
   panel.grid.minor=element_blank(),
   panel.background=element_rect(colour="black",size=1.2,fill=NA),
   axis.line=element_line(colour="black"),


   axis.title.y=element_text(vjust=2.5),
   axis.title.x=element_text(vjust=0.5),

   axis.text.x=element_text(size=20, colour="black"),
   axis.text.y=element_text(size=20, colour="black"),

   axis.ticks.x=element_line(size=2, colour="black"),
   axis.ticks.y=element_line(size=2, colour="black")

   )
```

```r
nydata<-read.csv(file.choose())
summary(nydata)
```

```
##   Study.number                     Citation             Sample.ID   
##  Min.   : 1.00   Schaller et al 2001   :  81   2            :   5  
##  1st Qu.:26.00   Reuter 2005           :  79   4            :   5  
##  Median :48.00   Duxbury 2008          :  70   3            :   4  
##  Mean   :45.09   Duxbury et. al. (2014):  66   05-3R-11b-SAL:   3  
##  3rd Qu.:69.00   Ouimet et al 2009     :  66   06-3R-26-MEK :   3  
##  Max.   :82.00   Miller et al. (2013)  :  59   06-3R-27-MEK :   3  
##                  (Other)               :1368   (Other)      :1766  
##      Slope              RockType   Precipitation    Vegetation    
##  Min.   : 0.00              : 68   Min.   :   3   Min.   :  0.00  
##  1st Qu.: 7.00    Mixed     :  1   1st Qu.: 582   1st Qu.: 13.00  
##  Median :14.40   Igneous    :354   Median : 949   Median : 45.00  
##  Mean   :15.09   Metamorphic:416   Mean   :1009   Mean   : 43.84  
##  3rd Qu.:21.70   Mixed      :606   3rd Qu.:1229   3rd Qu.: 76.00  
##  Max.   :50.00   Sedimentary:344   Max.   :3265   Max.   :100.00  
##                                                   NA's   :158     
##     Erosion        
##  Min.   :   0.07  
##  1st Qu.:  14.66  
##  Median :  40.55  
##  Mean   : 167.87  
##  3rd Qu.: 141.07  
##  Max.   :4119.53  
## 
```

```r
nydata = nydata[!is.na(nydata$Vegetation),]
```

Plotting erosion rate against Slope

```r
ggplot(nydata, aes(x=Slope, y=Erosion)) + geom_point() +
  geom_smooth(method="lm") + b + scale_y_log10()
```

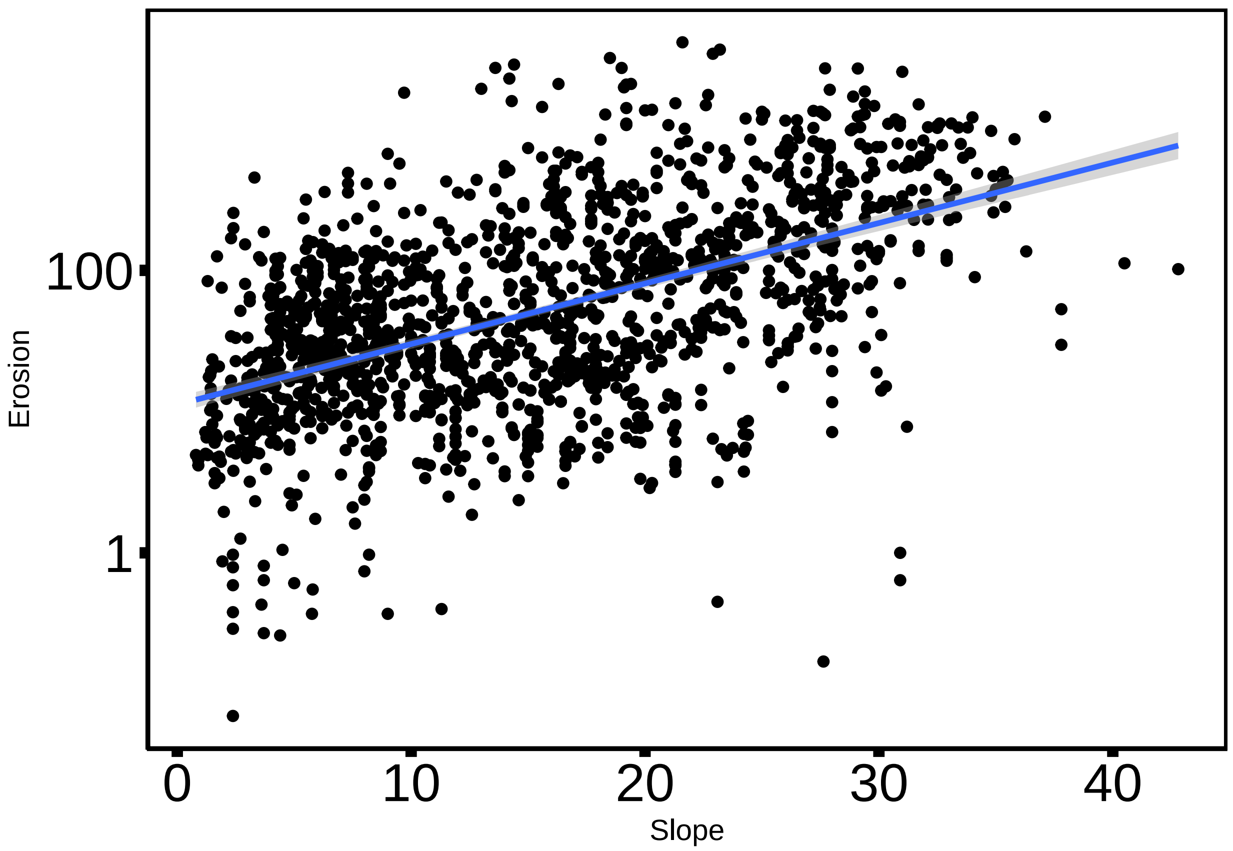

#correlation test for Slope:

```
regression.m1<-lm(log(Erosion) ~ Slope, data=nydata)
plot(regression.m1)
```

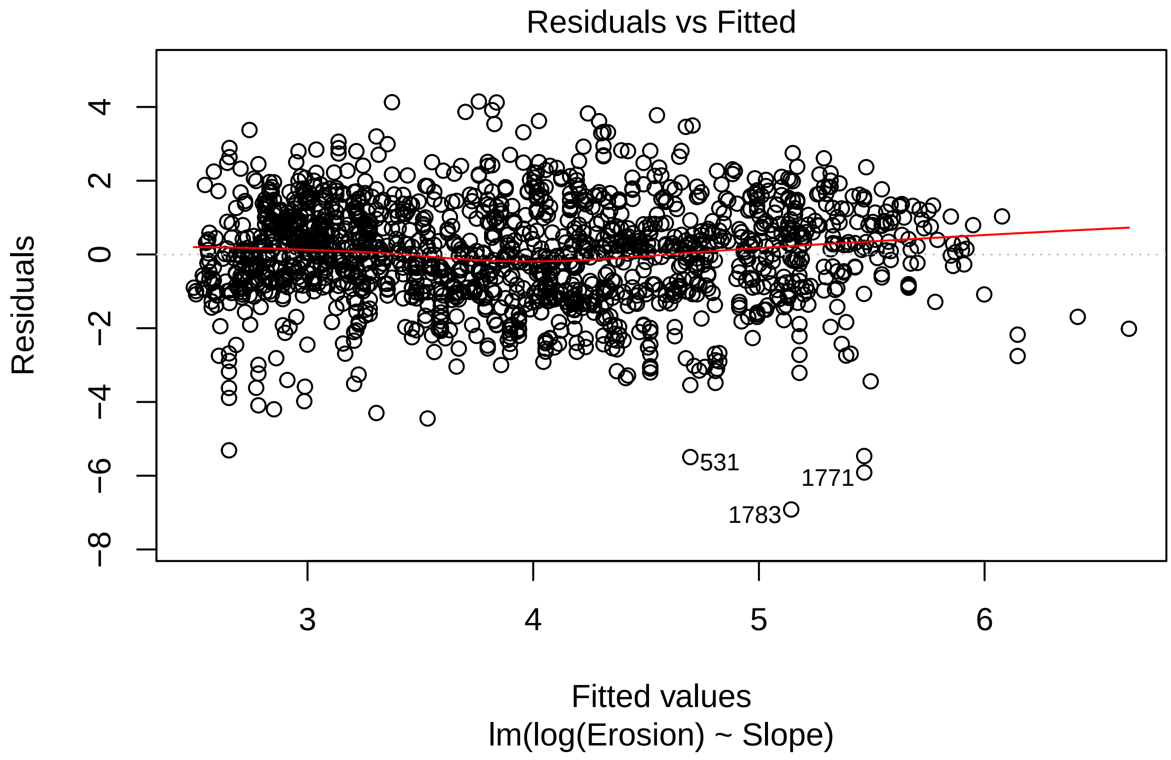
Residuals vs Fitted
Residuals
4
2
0
−2
−4
−6
−8
3
4
5
6
531
1771
1783
Fitted values
lm(log(Erosion) ~ Slope)

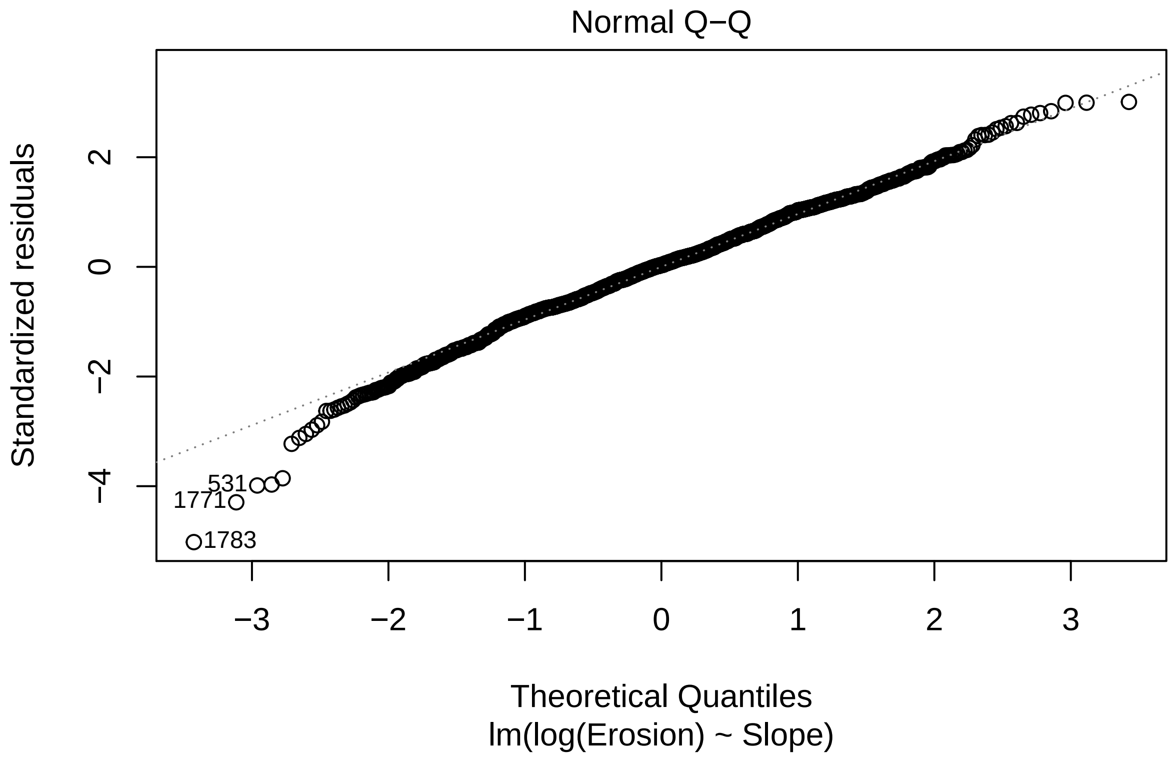
Normal Q−Q
Standardized residuals
Theoretical Quantiles
lm(log(Erosion) ~ Slope)
531
1771
1783
−4
−2
0
2
−3
−2
−1
0
1
2
3

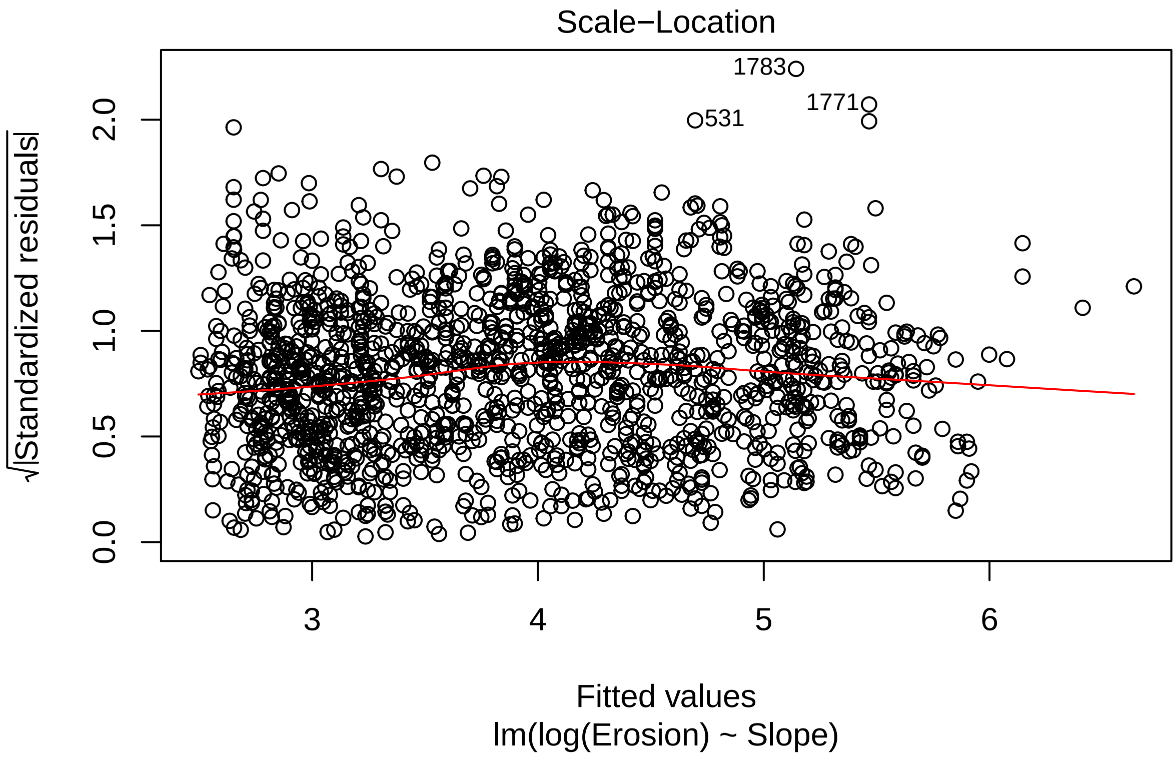

Scale−Location
√|Standardized residuals|
0.0
0.5
1.0
1.5
2.0
3
4
5
6
1783
1771
531
Fitted values
lm(log(Erosion) ~ Slope)

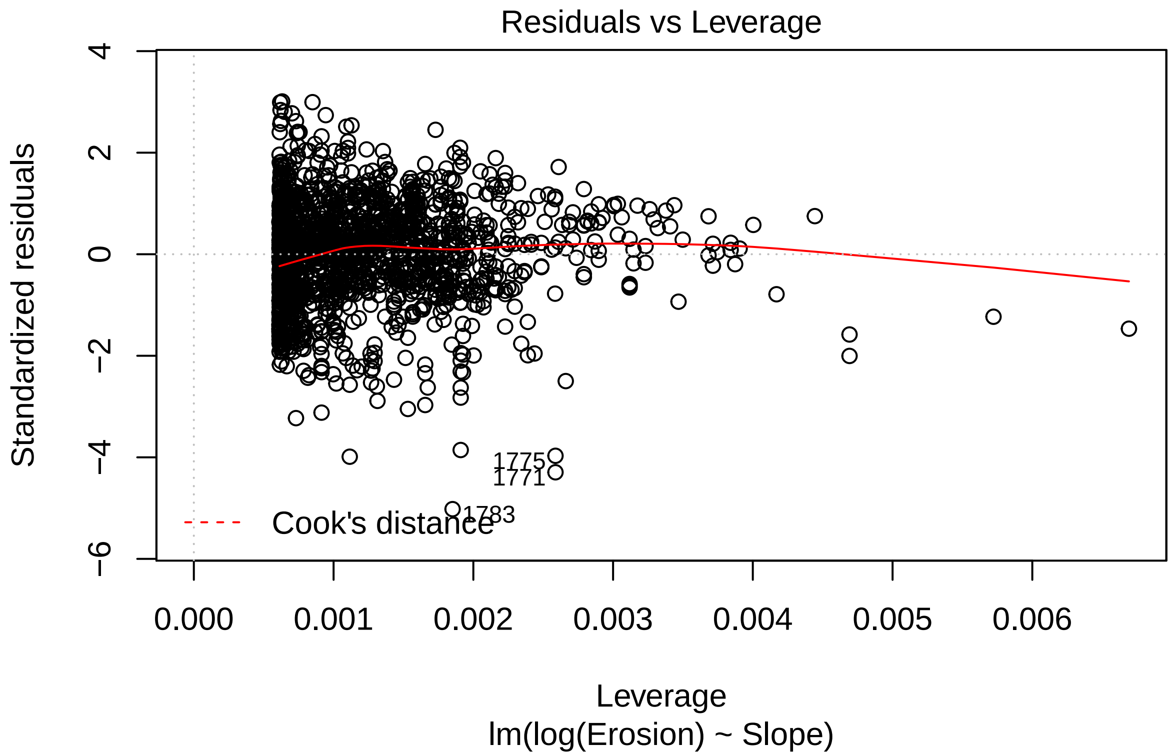


```r
summary(regression.m1)
```

```
##
## Call:
## lm(formula = log(Erosion) ~ Slope, data = nydata)
##
## Residuals:
##     Min      1Q  Median      3Q     Max
## -6.9150 -0.8939  0.0420  0.8970  4.1471
##
## Coefficients:
##             Estimate Std. Error t value Pr(>|t|)
## (Intercept) 2.417298   0.068075   35.51   <2e-16 ***
## Slope       0.098653   0.003888   25.38   <2e-16 ***
## ---
## Signif. codes:  0 '***' 0.001 '**' 0.01 '*' 0.05 '.' 0.1 ' ' 1
##
## Residual standard error: 1.379 on 1629 degrees of freedom
## Multiple R-squared:  0.2833, Adjusted R-squared:  0.2829
## F-statistic:   644 on 1 and 1629 DF,  p-value: < 2.2e-16
```

Plotting erosion rate against precipitation:

```r
ggplot(nydata, aes(x=Precipitation, y=Erosion)) + geom_point() +
  geom_smooth(method="lm") + b + scale_y_log10()
```

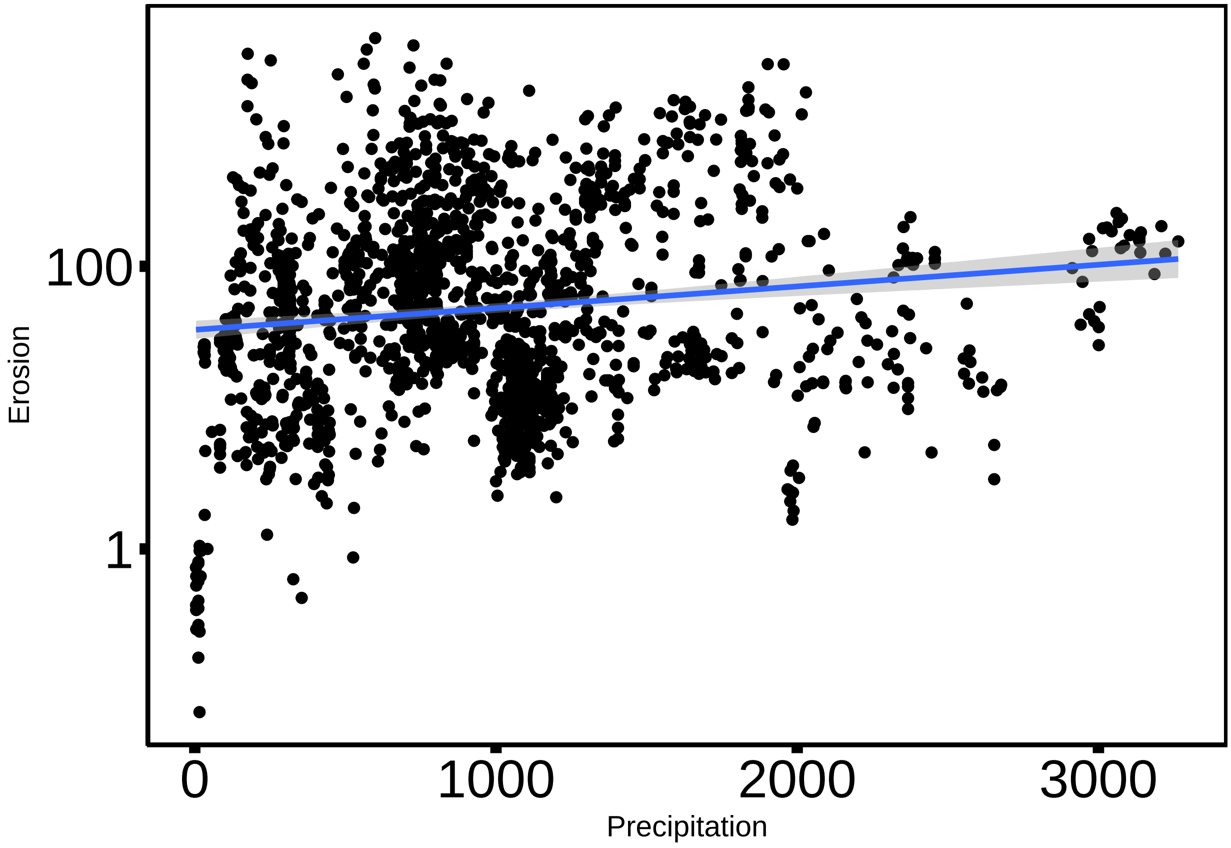

#correlation of precipitation with erosion rate:

```r
regression.m2<-lm(log(Erosion) ~ Precipitation, data=nydata)
plot(regression.m2)
```

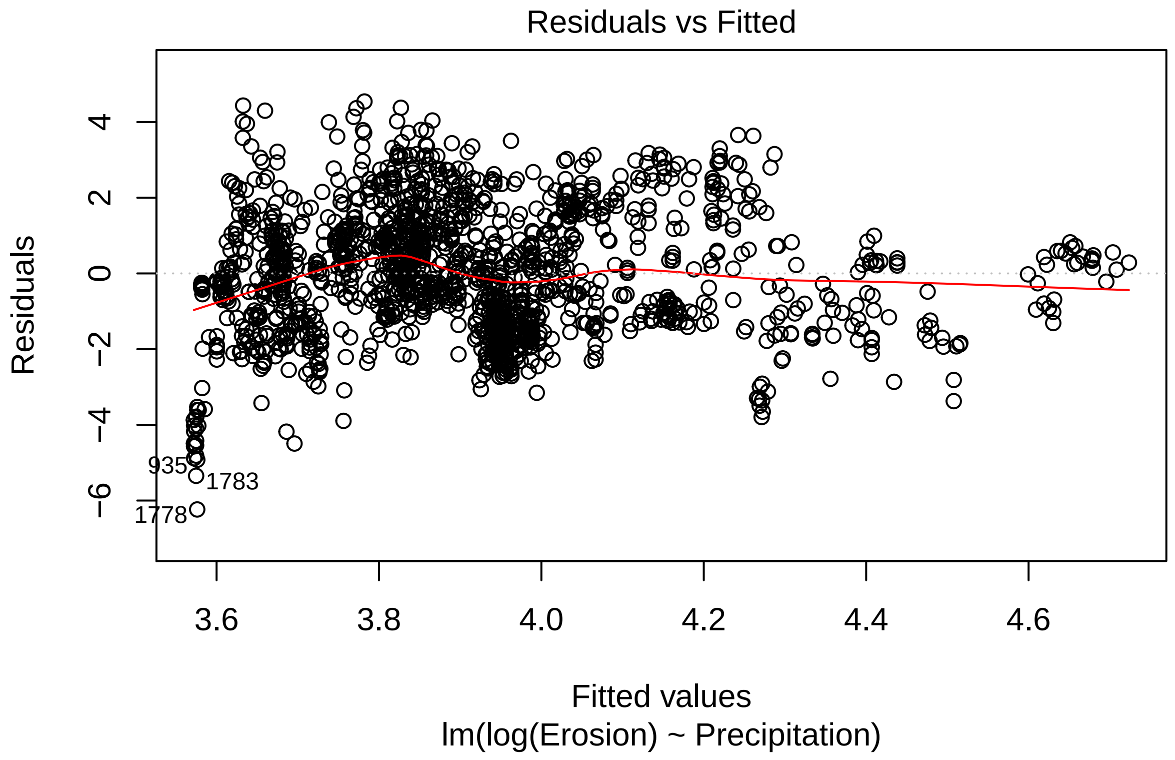
Residuals vs Fitted
Residuals
4
2
0
−2
−4
−6
935
1783
1778
3.6
3.8
4.0
4.2
4.4
4.6
Fitted values
lm(log(Erosion) ~ Precipitation)

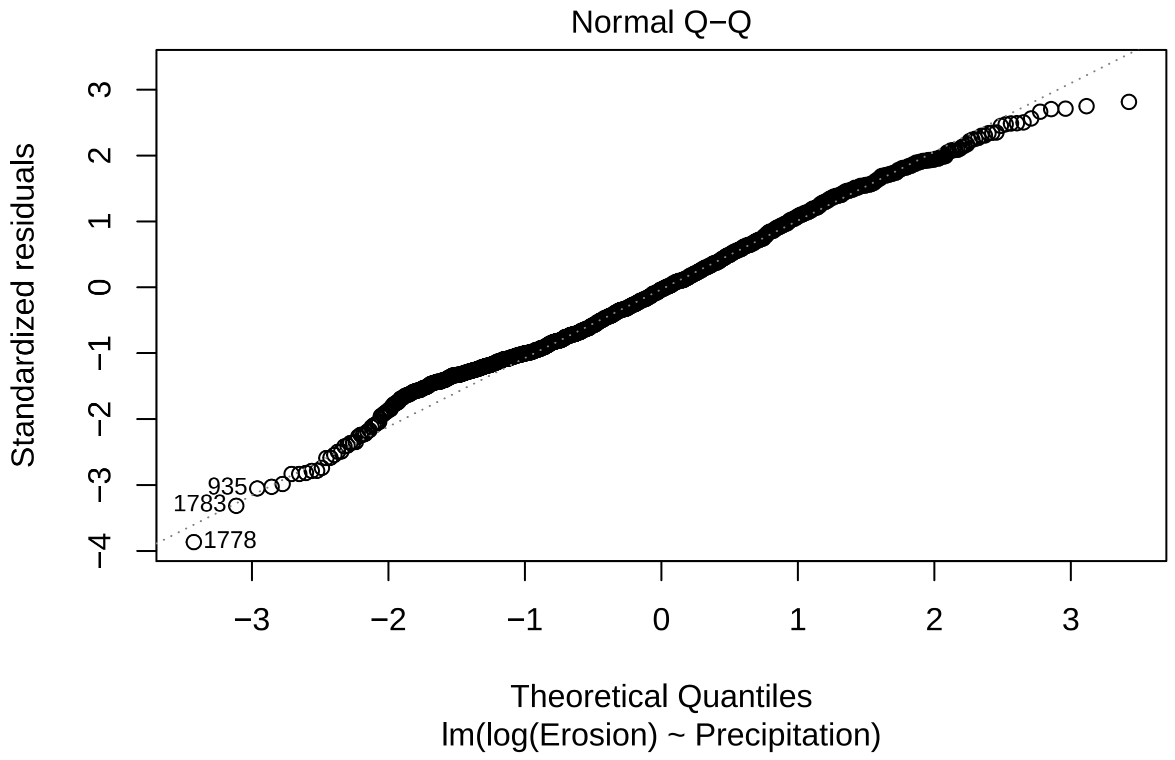

Normal Q−Q
Standardized residuals
Theoretical Quantiles
lm(log(Erosion) ~ Precipitation)
935
1783
1778

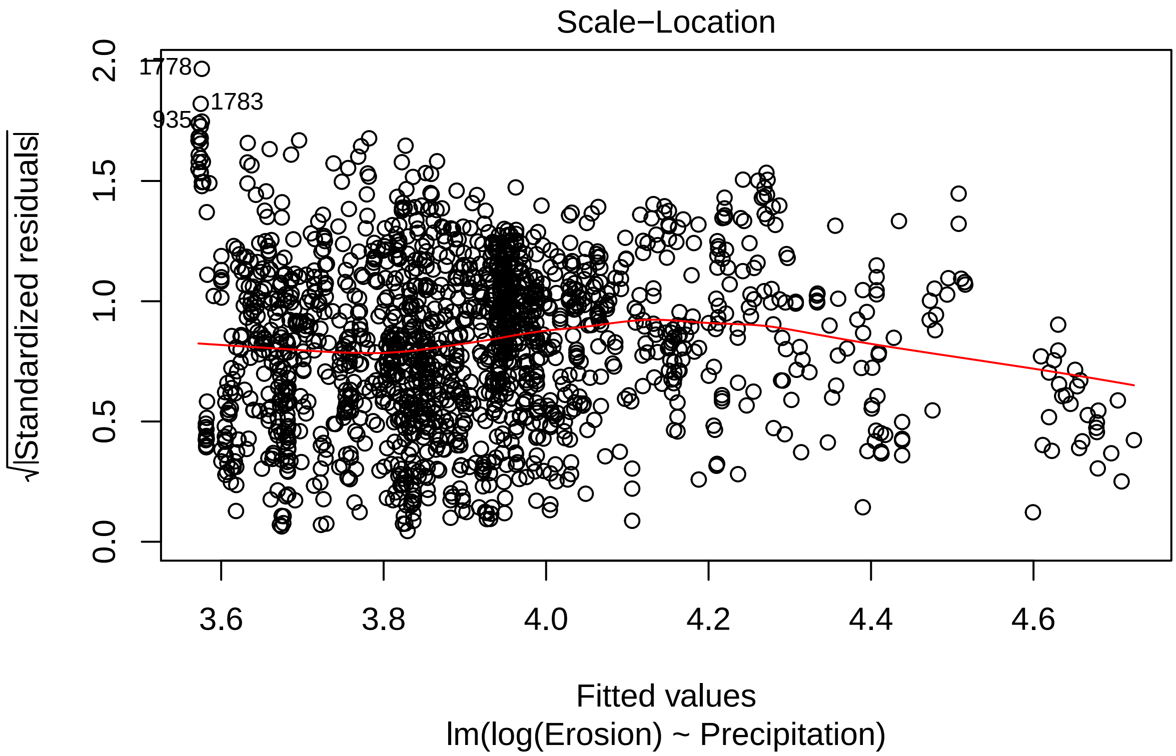
Scale−Location
√|Standardized residuals|
0.0
0.5
1.0
1.5
2.0
1778
1783
935
3.6
3.8
4.0
4.2
4.4
4.6
Fitted values
lm(log(Erosion) ~ Precipitation)

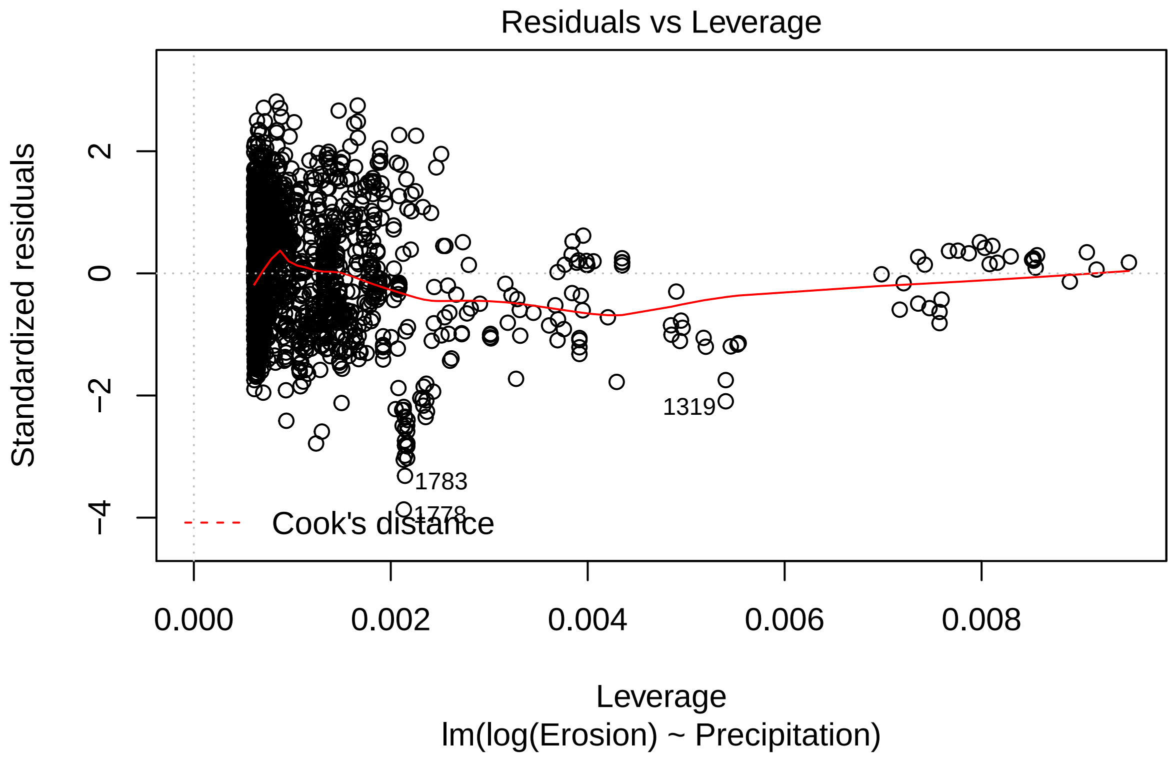


```
summary(regression.m2)
```

```
##
## Call:
## lm(formula = log(Erosion) ~ Precipitation, data = nydata)
##
## Residuals:
##     Min      1Q  Median      3Q     Max
## -6.2354 -1.1825 -0.0532  1.0871  4.5414
##
## Coefficients:
##                Estimate Std. Error t value Pr(>|t|)
## (Intercept)   3.5706068  0.0754383  47.332  < 2e-16 ***
## Precipitation 0.0003531  0.0000662   5.334 1.09e-07 ***
## ---
## Signif. codes:  0 '***' 0.001 '**' 0.01 '*' 0.05 '.' 0.1 ' ' 1
##
## Residual standard error: 1.615 on 1629 degrees of freedom
## Multiple R-squared:  0.01717,    Adjusted R-squared:  0.01656
## F-statistic: 28.46 on 1 and 1629 DF,  p-value: 1.093e-07
```

Selecting best suited model for Precipitation

```r
nydata$Precipitation= as.numeric(nydata$Precipitation)
nydata = mutate(nydata,
              Precipitation2 = Precipitation * Precipitation,
              Precipitation3 = Precipitation * Precipitation* Precipitation,
              Precipitation4 = Precipitation * Precipitation * Precipitation * Precipitation,
              Precipitation5 = Precipitation * Precipitation * Precipitation* Precipitation * Precipita
              Precipitation6 = Precipitation * Precipitation * Precipitation* Precipitation * Precipita
              Precipitation7 = Precipitation * Precipitation * Precipitation* Precipitation * Precipita
              Precipitation8 = Precipitation * Precipitation * Precipitation* Precipitation * Precipita

model.1 = lm (log(Erosion) ~ Precipitation,                               data=nydata)
model.2 = lm (log(Erosion) ~ Precipitation + Precipitation2,              data=nydata)
model.3 = lm (log(Erosion) ~ Precipitation + Precipitation2 + Precipitation3, data=nydata)
model.4 = lm (log(Erosion) ~ Precipitation + Precipitation2 + Precipitation3 + Precipitation4, data=nyda
model.5 = lm (log(Erosion) ~ Precipitation + Precipitation2 + Precipitation3 + Precipitation4 + Precipi
model.6 = lm (log(Erosion) ~ Precipitation + Precipitation2 + Precipitation3 + Precipitation4 + Precipi
model.7 = lm (log(Erosion) ~ Precipitation + Precipitation2 + Precipitation3 + Precipitation4 + Precipi
model.8 = lm (log(Erosion) ~ Precipitation + Precipitation2 + Precipitation3 + Precipitation4 + Precipi
```

AIC of models:

```r
AIC(model.1, model.2, model.3, model.4, model.5, model.6, model.7, model.8)
```

```
##         df      AIC
## model.1  3 6195.295
## model.2  4 6177.035
## model.3  5 6141.354
## model.4  6 6115.865
## model.5  7 6026.973
## model.6  8 6028.497
## model.7  9 5998.598
## model.8 10 5996.645
```

BIC Value of Models:

```r
BIC(model.1, model.2, model.3, model.4, model.5, model.6, model.7, model.8)
```

```
##         df      BIC
## model.1  3 6211.486
## model.2  4 6198.623
## model.3  5 6168.338
## model.4  6 6148.247
## model.5  7 6064.751
## model.6  8 6071.673
## model.7  9 6047.171
## model.8 10 6050.615
```

ANOVA of models:

```
anova(model.1, model.2, model.3, model.4, model.5, model.6, model.7, model.8)
```

```
## Analysis of Variance Table
##
## Model 1: log(Erosion) ~ Precipitation
## Model 2: log(Erosion) ~ Precipitation + Precipitation2
## Model 3: log(Erosion) ~ Precipitation + Precipitation2 + Precipitation3
## Model 4: log(Erosion) ~ Precipitation + Precipitation2 + Precipitation3 +
##     Precipitation4
## Model 5: log(Erosion) ~ Precipitation + Precipitation2 + Precipitation3 +
##     Precipitation4 + Precipitation5
## Model 6: log(Erosion) ~ Precipitation + Precipitation2 + Precipitation3 +
##     Precipitation4 + Precipitation5 + Precipitation6
## Model 7: log(Erosion) ~ Precipitation + Precipitation2 + Precipitation3 +
##     Precipitation4 + Precipitation5 + Precipitation6 + Precipitation7
## Model 8: log(Erosion) ~ Precipitation + Precipitation2 + Precipitation3 +
##     Precipitation4 + Precipitation5 + Precipitation6 + Precipitation7 +
##     Precipitation8
##   Res.Df    RSS Df Sum of Sq       F    Pr(>F)
## 1   1629 4246.5
## 2   1628 4194.1  1    52.424 22.8123 1.947e-06 ***
## 3   1627 4098.3  1    95.786 41.6815 1.415e-10 ***
## 4   1626 4029.8  1    68.493 29.8049 5.519e-08 ***
## 5   1625 3811.4  1   218.431 95.0507 < 2.2e-16 ***
## 6   1624 3810.3  1     1.110  0.4832   0.48707
## 7   1623 3736.5  1    73.798 32.1132 1.718e-08 ***
## 8   1622 3727.4  1     9.045  3.9358   0.04744 *
## ---
## Signif. codes:  0 '***' 0.001 '**' 0.01 '*' 0.05 '.' 0.1 ' ' 1
```

Plotting erosion rate against precipitation:

```
ggplot(nydata, aes(x=Precipitation, y=Erosion)) + geom_point() +
  stat_smooth(method="lm", formula=y ~ poly(x, 3, raw=TRUE)) + b +scale_y_log10()
```

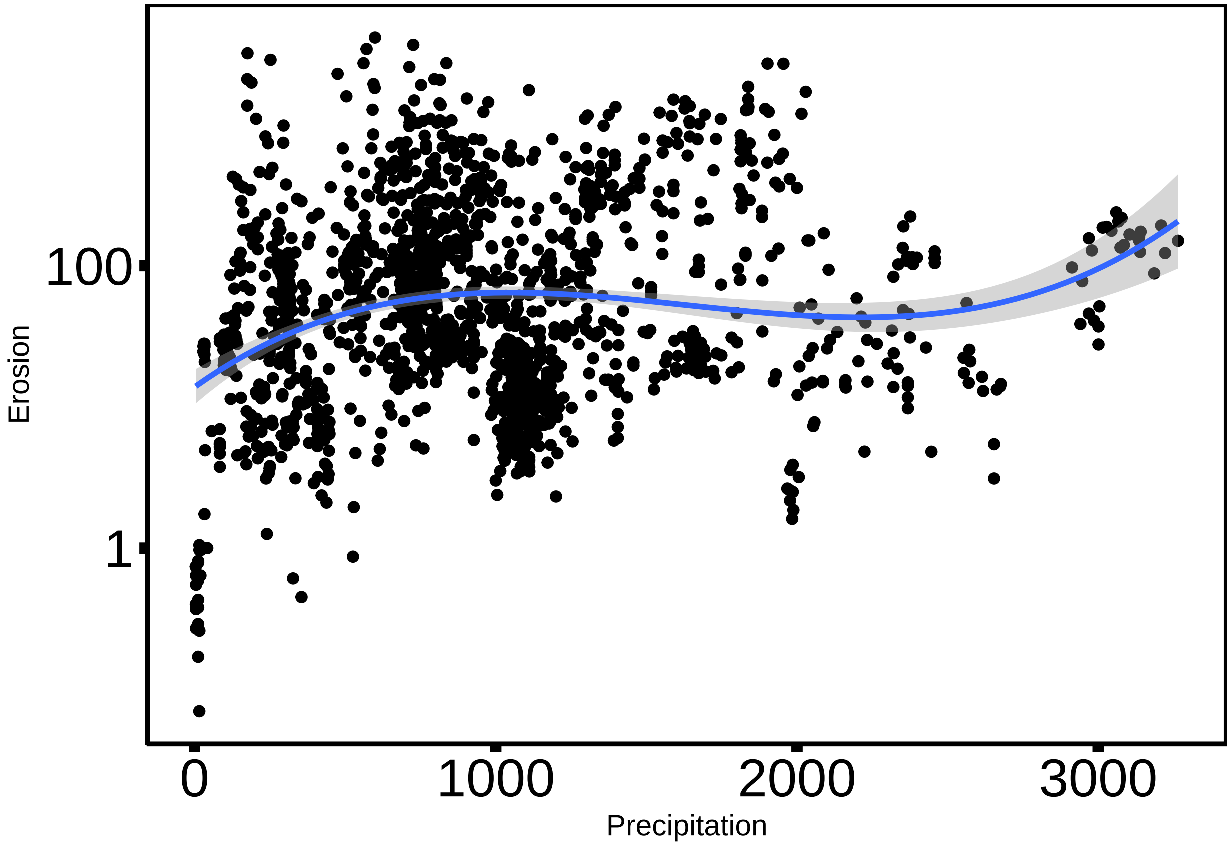

#Checking correlation of Precip in polynomial:

```
regression.m3<-lm(log(Erosion) ~ poly(Precipitation, 3), data=nydata)
plot(regression.m3)
```

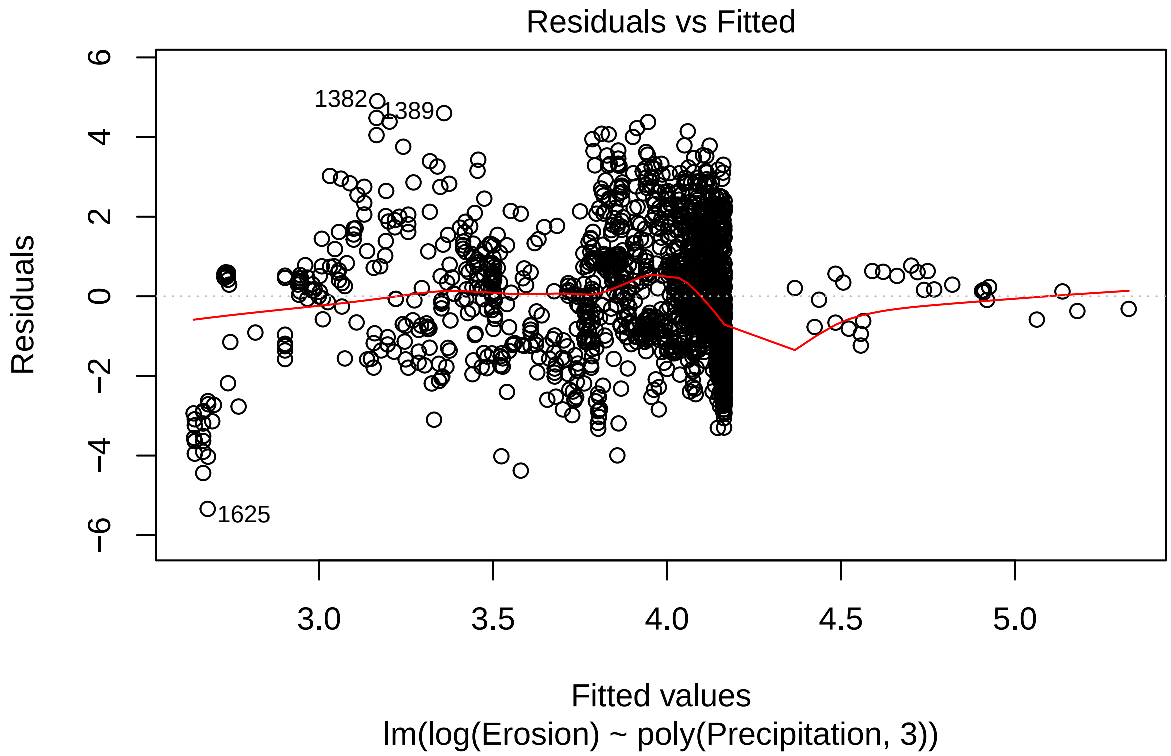

Residuals vs Fitted
Residuals
Fitted values
lm(log(Erosion) ~ poly(Precipitation, 3))
1382
1389
1625
6
4
2
0
−2
−4
−6
3.0
3.5
4.0
4.5
5.0

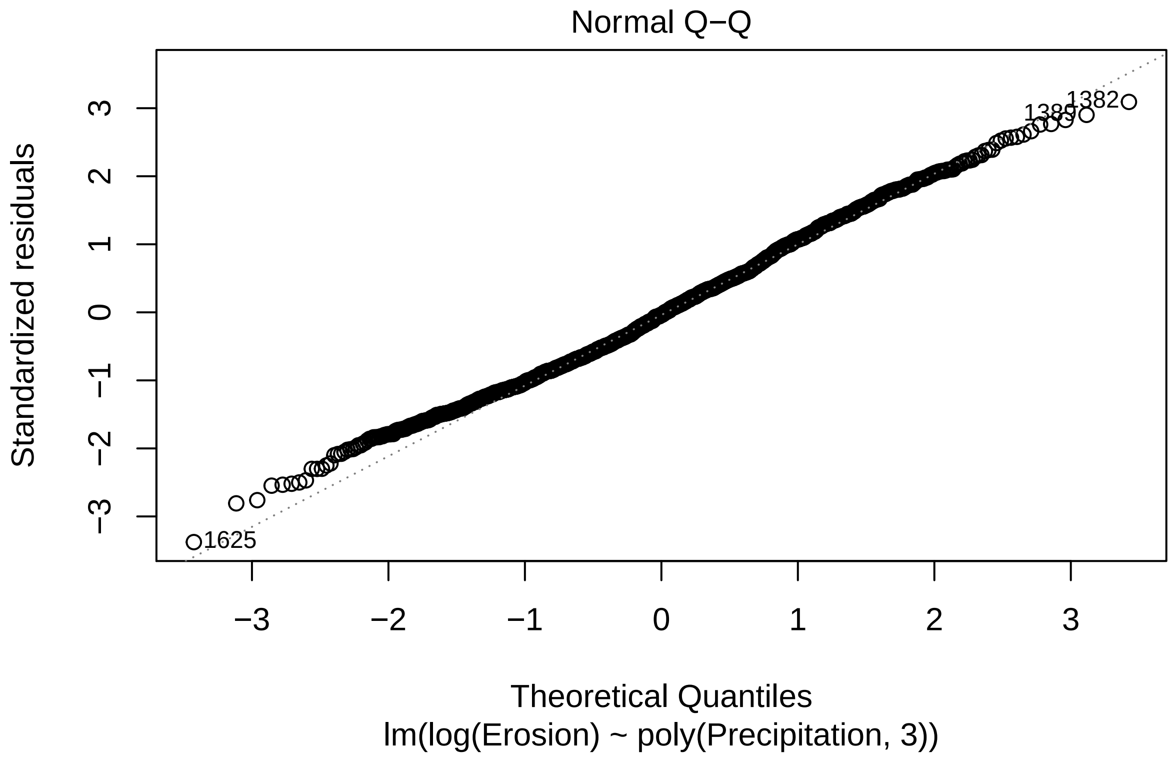
Normal Q−Q
Standardized residuals
3
2
1
0
−1
−2
−3
1382
1389
1625
−3
−2
−1
0
1
2
3
Theoretical Quantiles
lm(log(Erosion) ~ poly(Precipitation, 3))

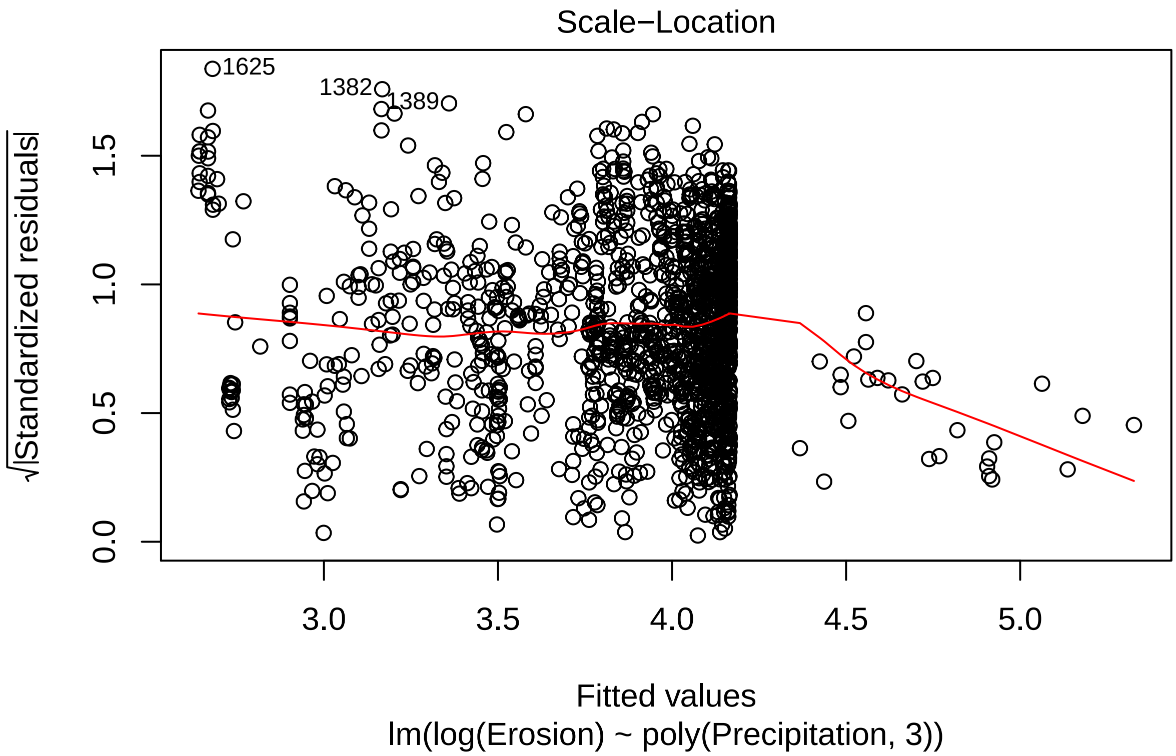
Scale−Location
1625
1382
1389
1.5
1.0
0.5
0.0
√|Standardized residuals|
3.0
3.5
4.0
4.5
5.0
Fitted values
lm(log(Erosion) ~ poly(Precipitation, 3))

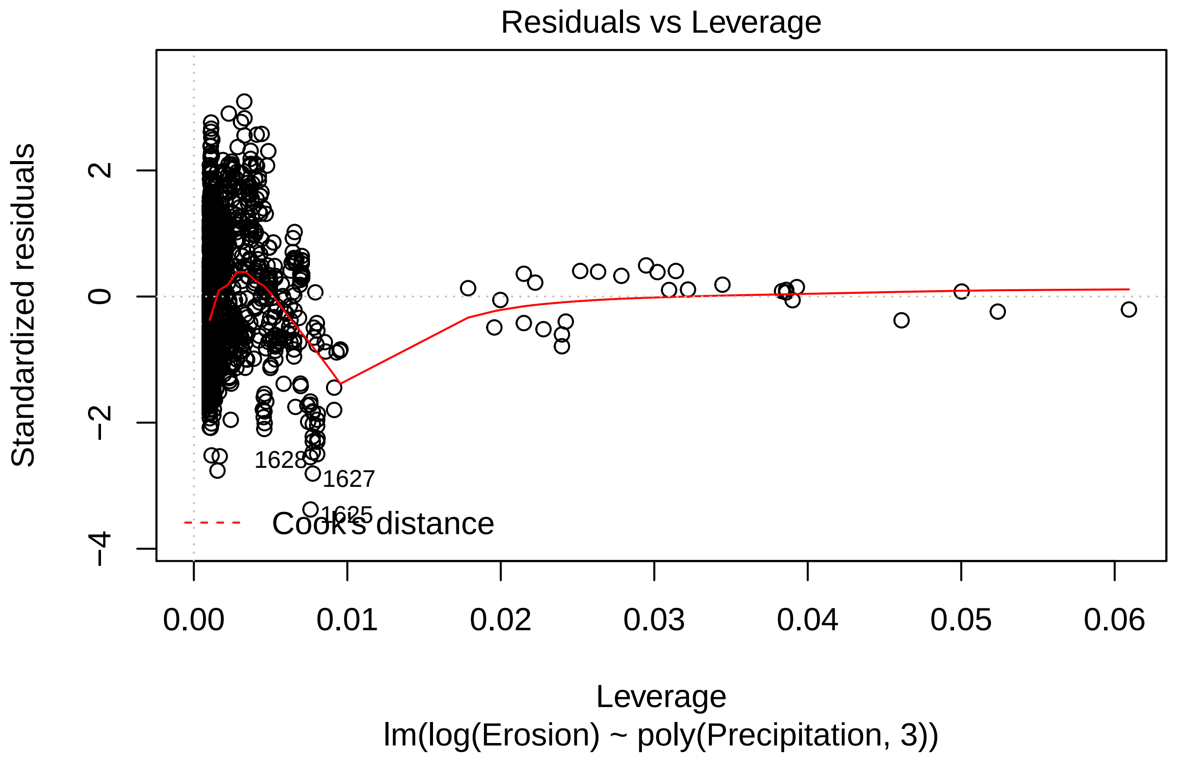


```r
summary(regression.m3)
```

```
##
## Call:
## lm(formula = log(Erosion) ~ poly(Precipitation, 3), data = nydata)
##
## Residuals:
##     Min      1Q  Median      3Q     Max
## -5.3392 -1.1717 -0.0617  1.0488  4.8999
##
## Coefficients:
##                         Estimate Std. Error t value Pr(>|t|)
## (Intercept)               3.9119     0.0393  99.541  < 2e-16 ***
## poly(Precipitation, 3)1   8.6127     1.5871   5.427 6.61e-08 ***
## poly(Precipitation, 3)2  -7.2404     1.5871  -4.562 5.45e-06 ***
## poly(Precipitation, 3)3   9.7871     1.5871   6.167 8.79e-10 ***
## ---
## Signif. codes:  0 '***' 0.001 '**' 0.01 '*' 0.05 '.' 0.1 ' ' 1
##
## Residual standard error: 1.587 on 1627 degrees of freedom
## Multiple R-squared:  0.05147,    Adjusted R-squared:  0.04972
## F-statistic: 29.43 on 3 and 1627 DF,  p-value: < 2.2e-16
```

Plotting erosion rate against Vegtation:

```
ggplot(nydata, aes(x=Vegetation, y=Erosion)) + geom_point() +
  geom_smooth(method="lm") + b + scale_y_log10()
```

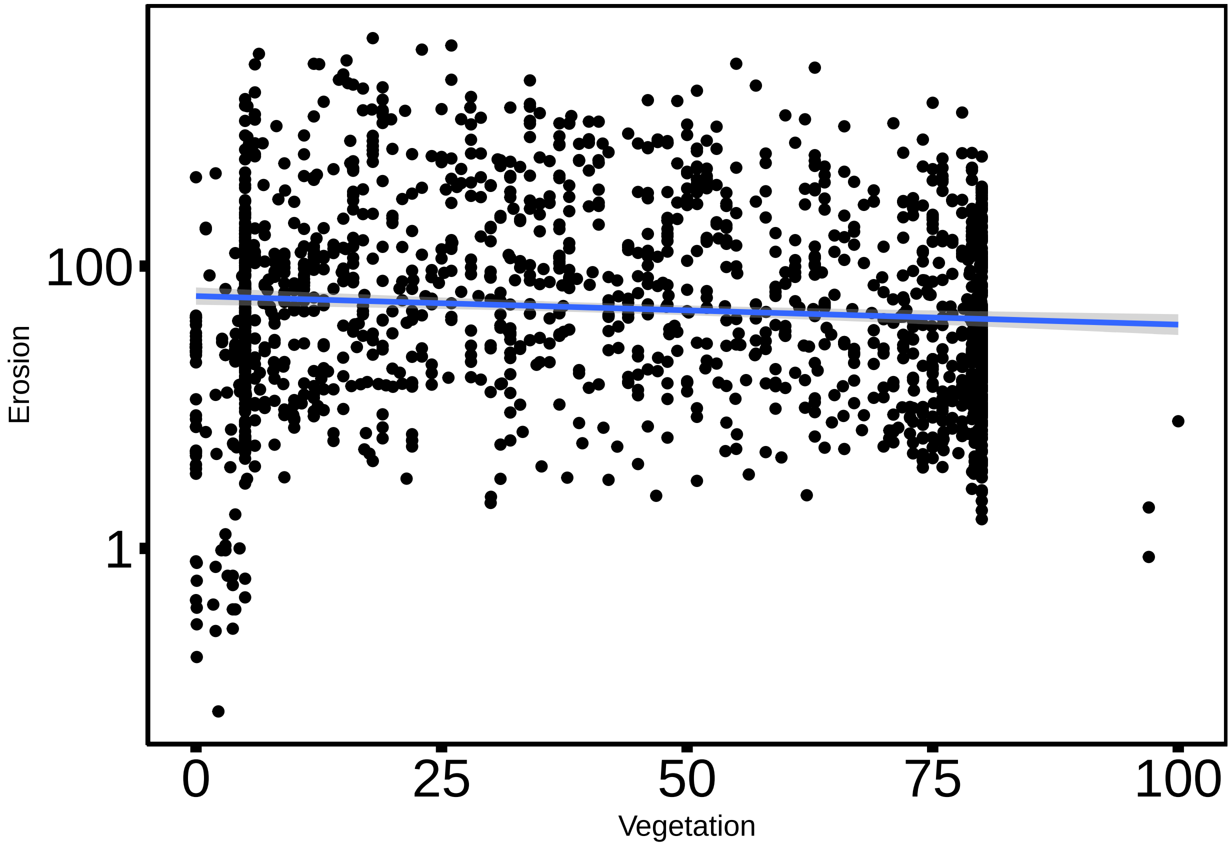

#correlation of vegetation with erosion:

```
regression.m4<-lm(log(Erosion) ~ Vegetation, data=nydata)
plot(regression.m4)
```

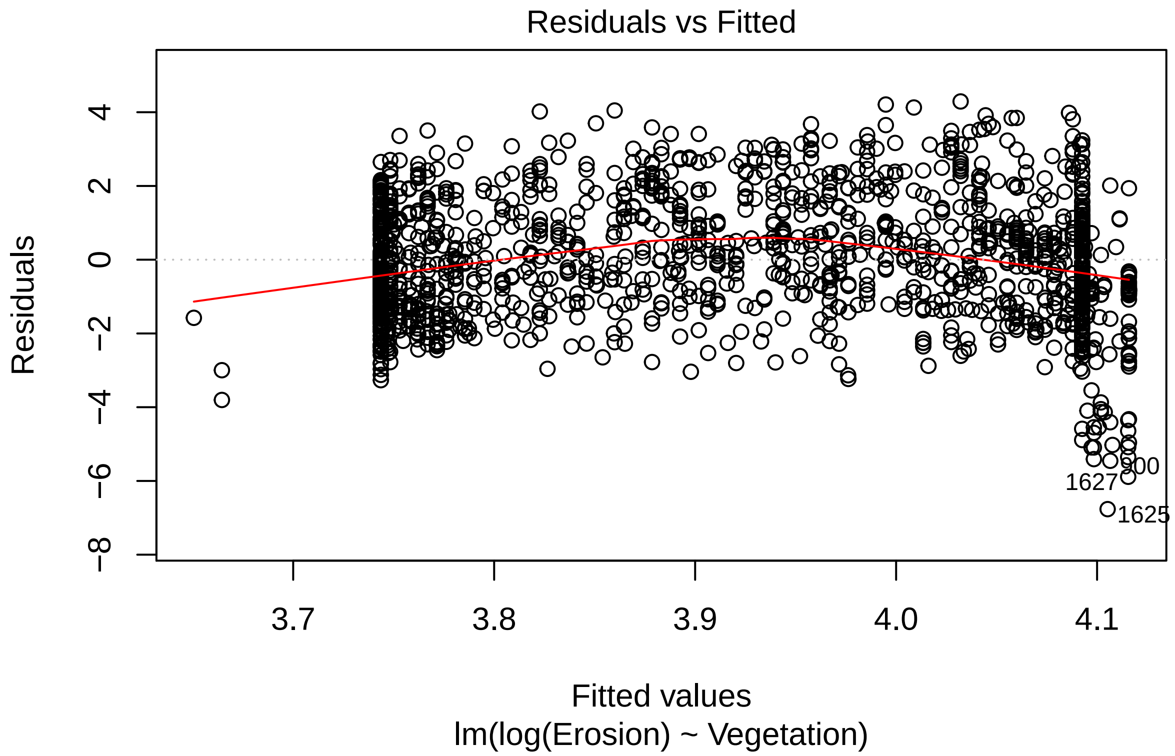
Residuals vs Fitted
Residuals
4
2
0
−2
−4
−6
−8
3.7
3.8
3.9
4.0
4.1
900
1627
1625
Fitted values
lm(log(Erosion) ~ Vegetation)

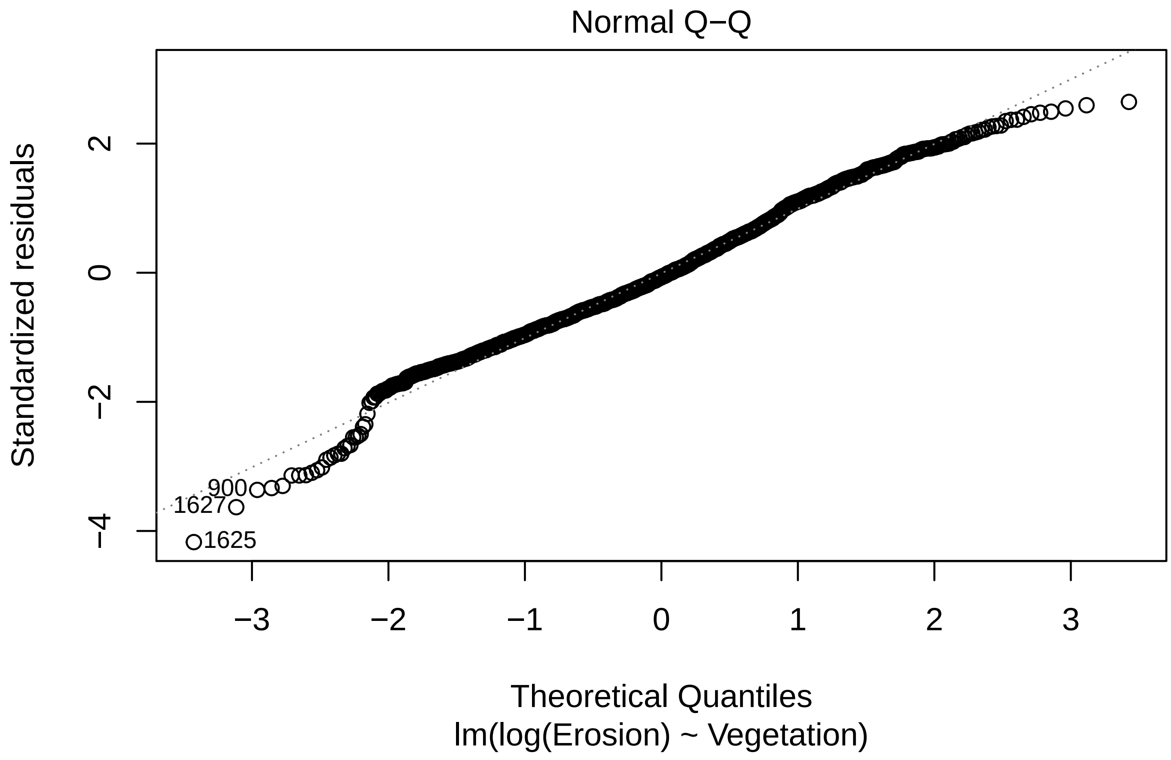
Normal Q−Q
Standardized residuals
Theoretical Quantiles
lm(log(Erosion) ~ Vegetation)
2
0
−2
−4
−3
−2
−1
0
1
2
3
900
1627
1625

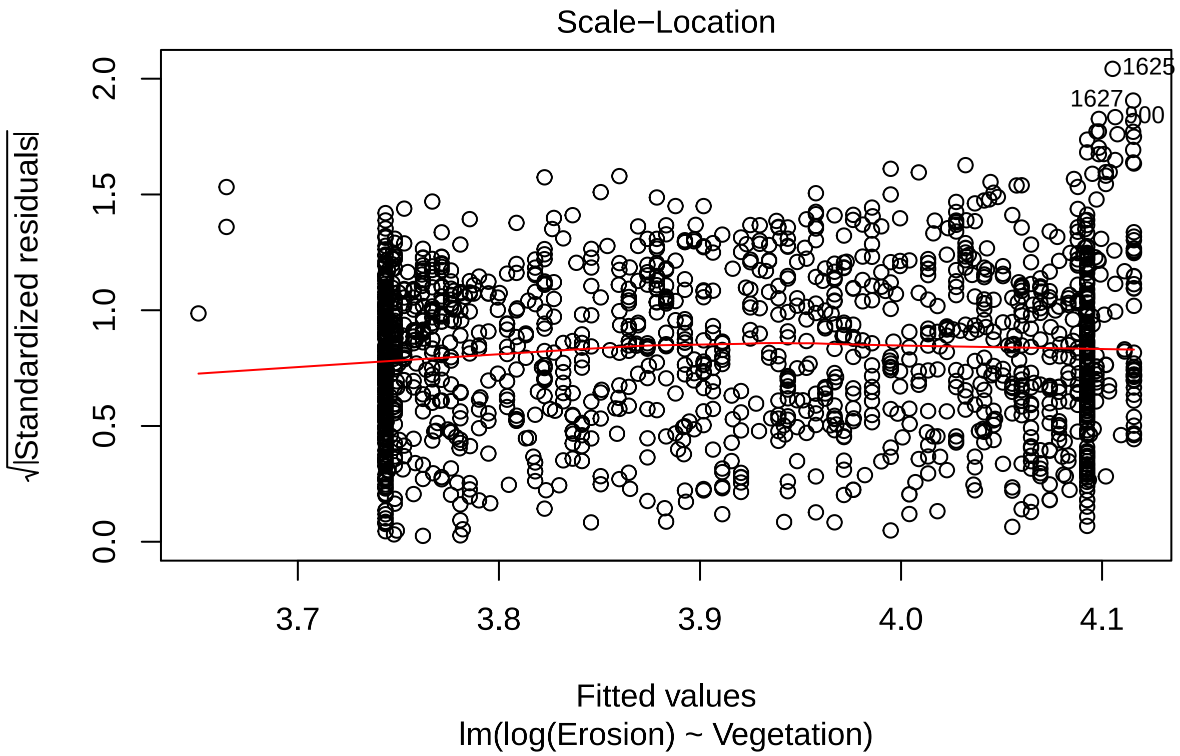
Scale−Location
√|Standardized residuals|
0.0
0.5
1.0
1.5
2.0
3.7
3.8
3.9
4.0
4.1
1625
1627
900
Fitted values
lm(log(Erosion) ~ Vegetation)

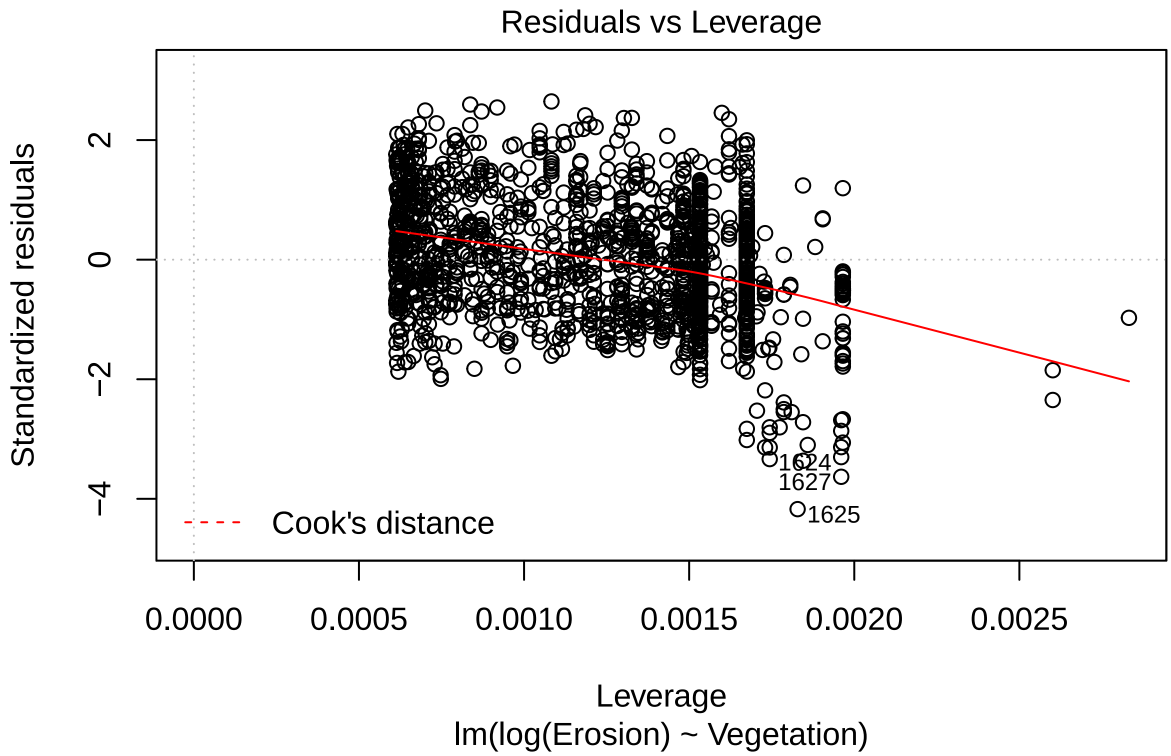


```r
summary(regression.m4)
```

```
##
## Call:
## lm(formula = log(Erosion) ~ Vegetation, data = nydata)
##
## Residuals:
##     Min      1Q  Median      3Q     Max
## -6.7645 -1.1110 -0.1094  1.0805  4.2914
##
## Coefficients:
##              Estimate Std. Error t value Pr(>|t|)
## (Intercept)  4.115855   0.071943  57.210  < 2e-16 ***
## Vegetation  -0.004653   0.001361  -3.418 0.000646 ***
## ---
## Signif. codes:  0 '***' 0.001 '**' 0.01 '*' 0.05 '.' 0.1 ' ' 1
##
## Residual standard error: 1.623 on 1629 degrees of freedom
## Multiple R-squared:  0.007122,   Adjusted R-squared:  0.006512
## F-statistic: 11.68 on 1 and 1629 DF,  p-value: 0.0006457
```

plotting vegetation with erosion rate on polynomial:

```r
ggplot(nydata, aes(x=Vegetation, y=Erosion)) + geom_point() +
  geom_smooth(method="lm", formula=y ~ poly(x, 2, raw=TRUE)) + b +scale_y_log10()
```

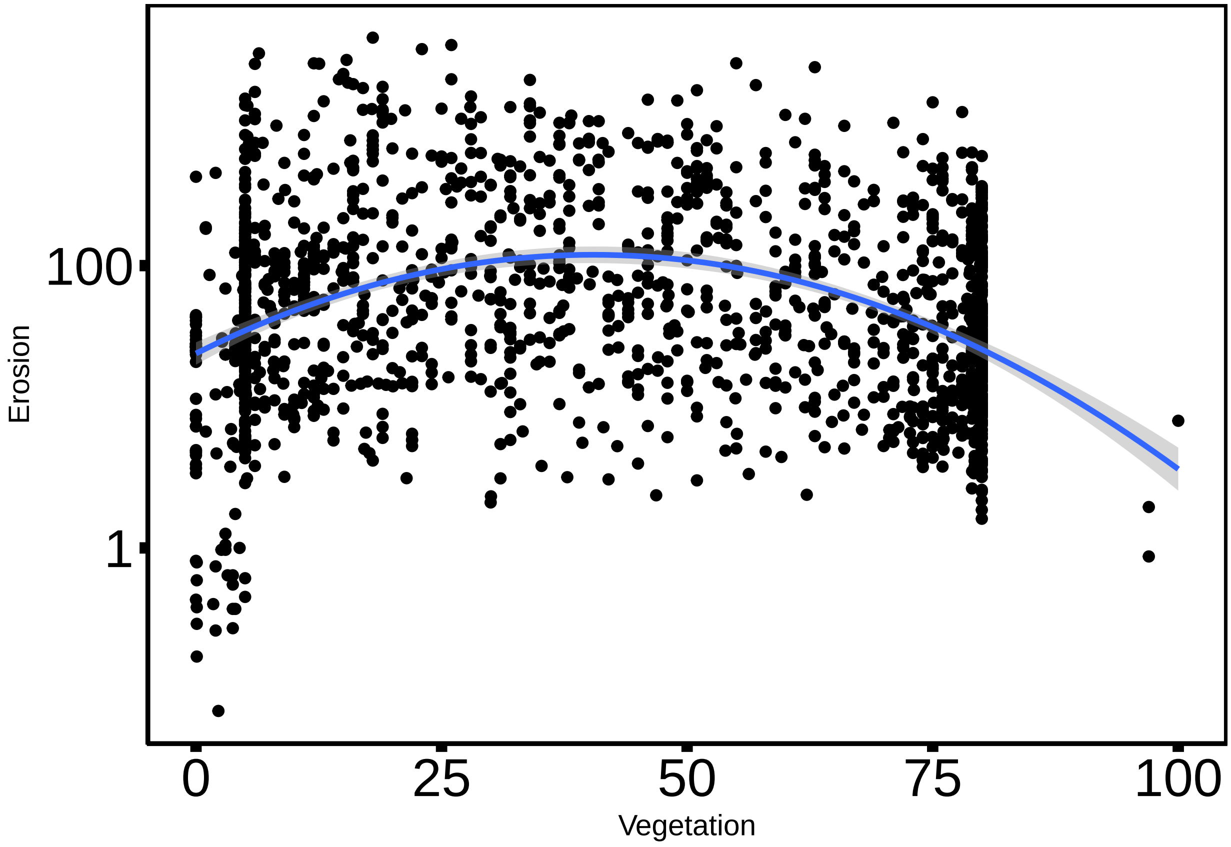

#correlation of erosion with poly vegetation:

```r
regression.m5<-lm(log(Erosion) ~ poly(Vegetation, 2), data=nydata)
plot(regression.m5)
```

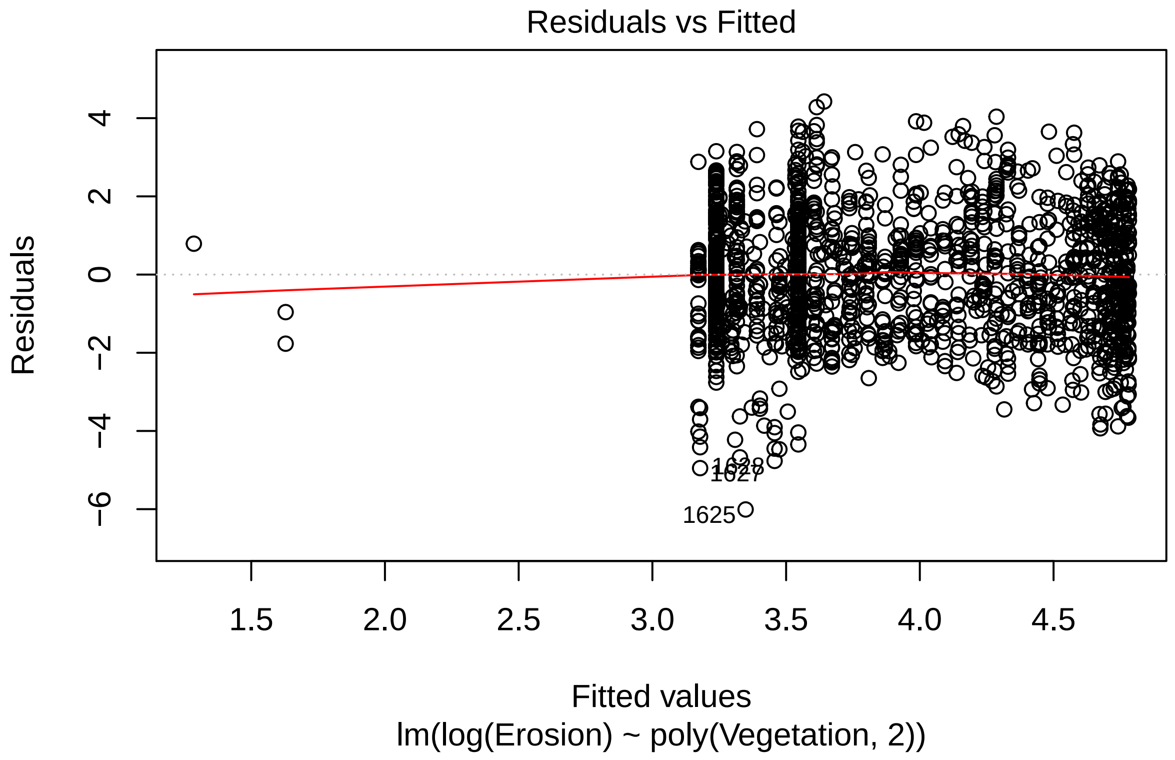

Residuals vs Fitted
Residuals
Fitted values
lm(log(Erosion) ~ poly(Vegetation, 2))
1625
1627
4
2
0
−2
−4
−6
1.5
2.0
2.5
3.0
3.5
4.0
4.5

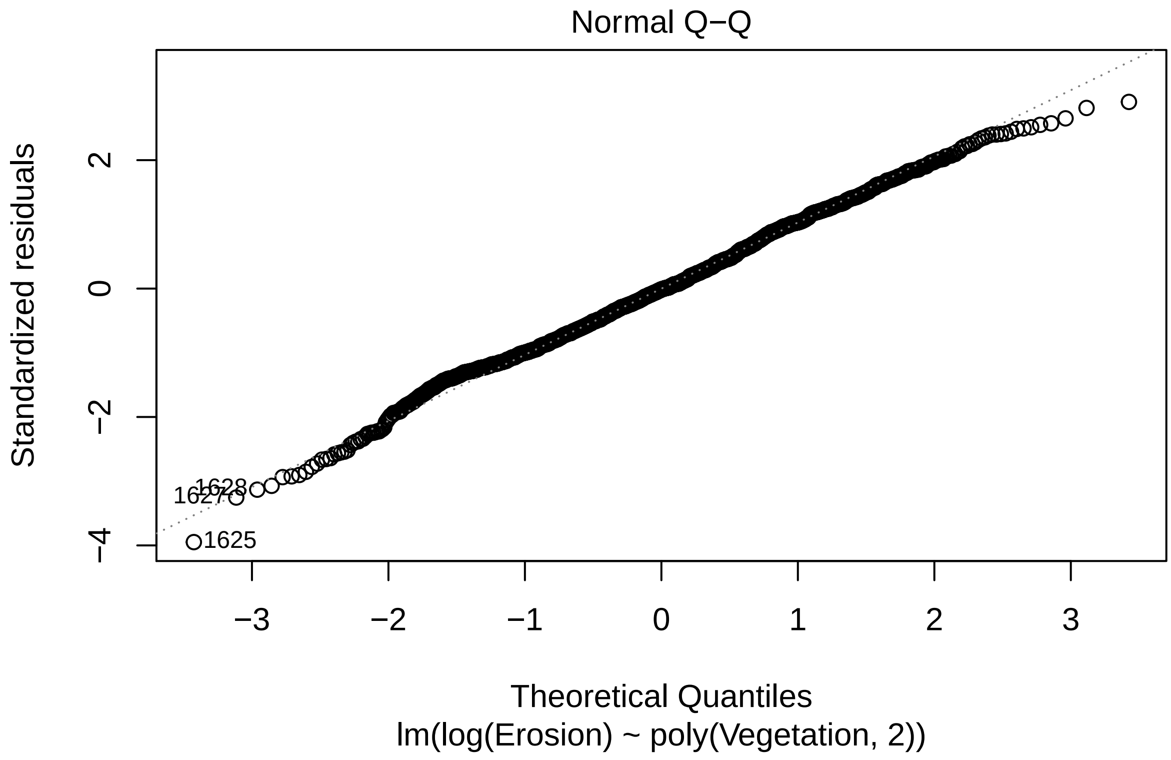
Normal Q−Q
Standardized residuals
Theoretical Quantiles
lm(log(Erosion) ~ poly(Vegetation, 2))
1628
1627
1625
−4
−2
0
2
−3
−2
−1
0
1
2
3

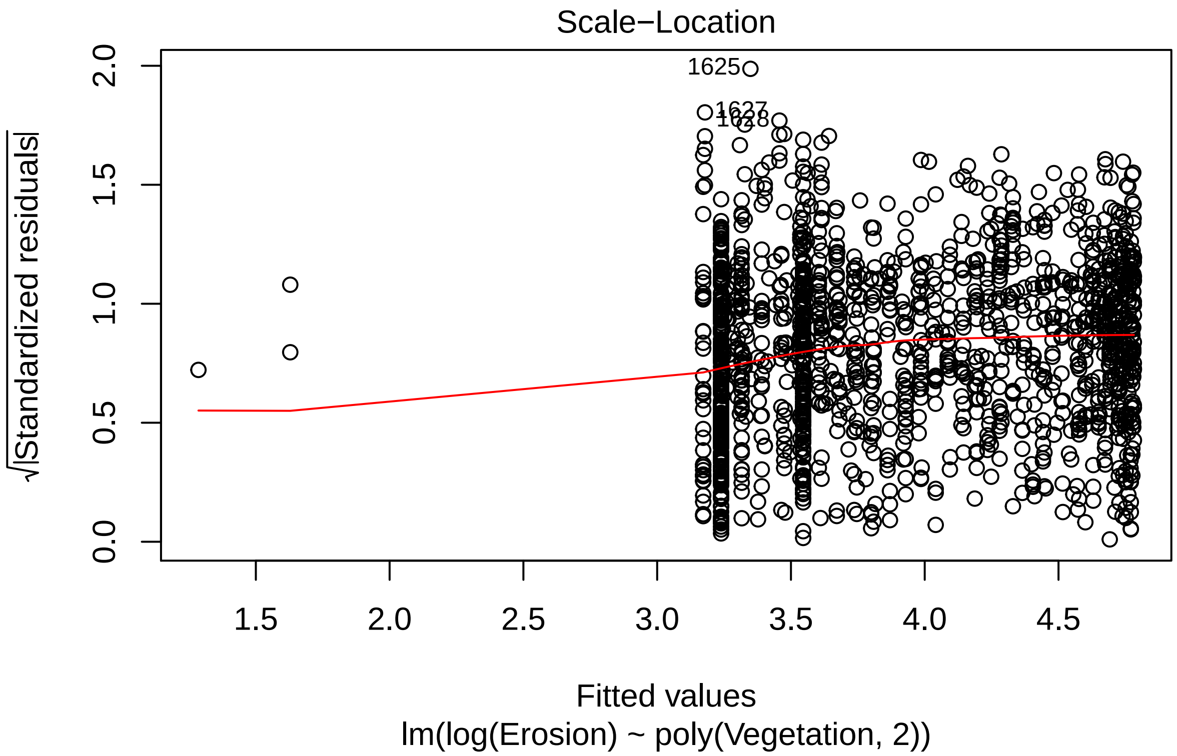
Scale−Location
√|Standardized residuals|
2.0
1.5
1.0
0.5
0.0
1625
1627
1628
1.5
2.0
2.5
3.0
3.5
4.0
4.5
Fitted values
lm(log(Erosion) ~ poly(Vegetation, 2))

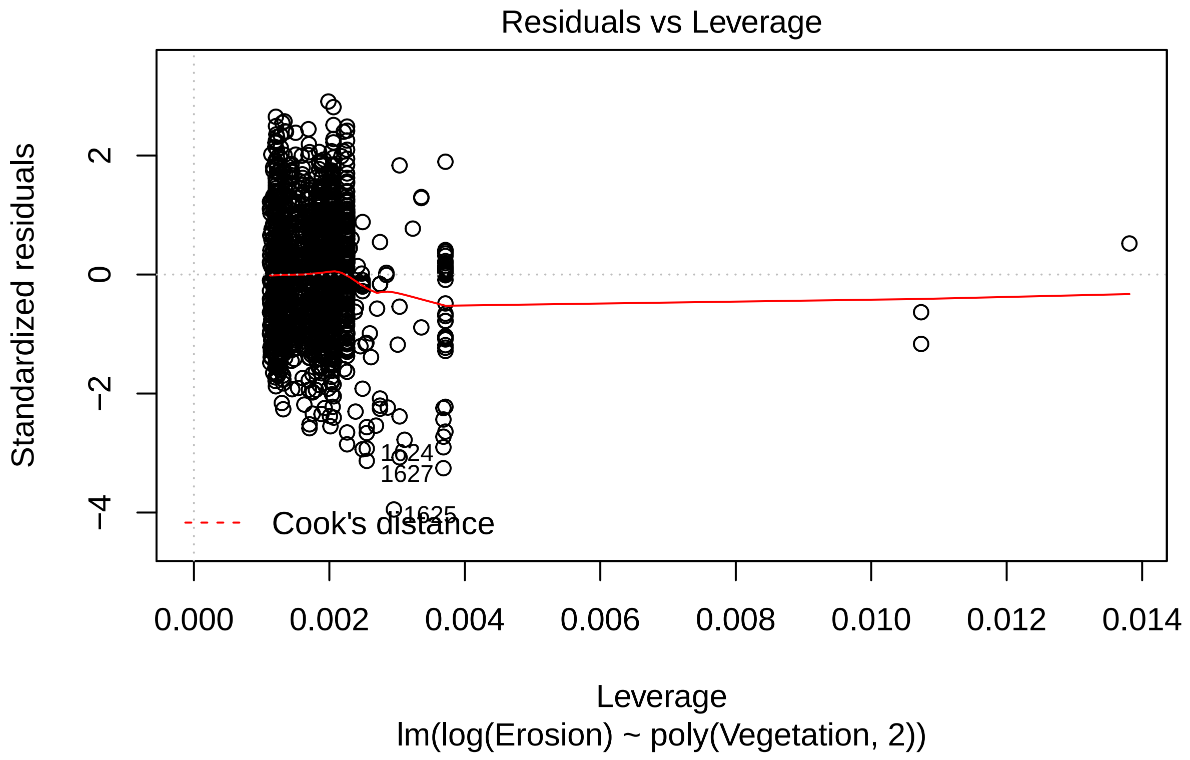


```r
summary(regression.m5)
```

```
##
## Call:
## lm(formula = log(Erosion) ~ poly(Vegetation, 2), data = nydata)
##
## Residuals:
##     Min      1Q  Median      3Q     Max
## -6.0078 -1.0597 -0.0201  1.0565  4.4252
##
## Coefficients:
##                      Estimate Std. Error t value Pr(>|t|)
## (Intercept)           3.91187    0.03773  103.67  < 2e-16 ***
## poly(Vegetation, 2)1  -5.54718    1.52386   -3.64 0.000281 ***
## poly(Vegetation, 2)2 -22.57114    1.52386  -14.81  < 2e-16 ***
## ---
## Signif. codes:  0 '***' 0.001 '**' 0.01 '*' 0.05 '.' 0.1 ' ' 1
##
## Residual standard error: 1.524 on 1628 degrees of freedom
## Multiple R-squared:  0.125,  Adjusted R-squared:  0.124
## F-statistic: 116.3 on 2 and 1628 DF,  p-value: < 2.2e-16
```

plotting vegegtation vs precipitation

```
ggplot(nydata, aes(x=Vegetation, y=Precipitation)) + geom_point() +
  geom_smooth(method="lm") + b
```

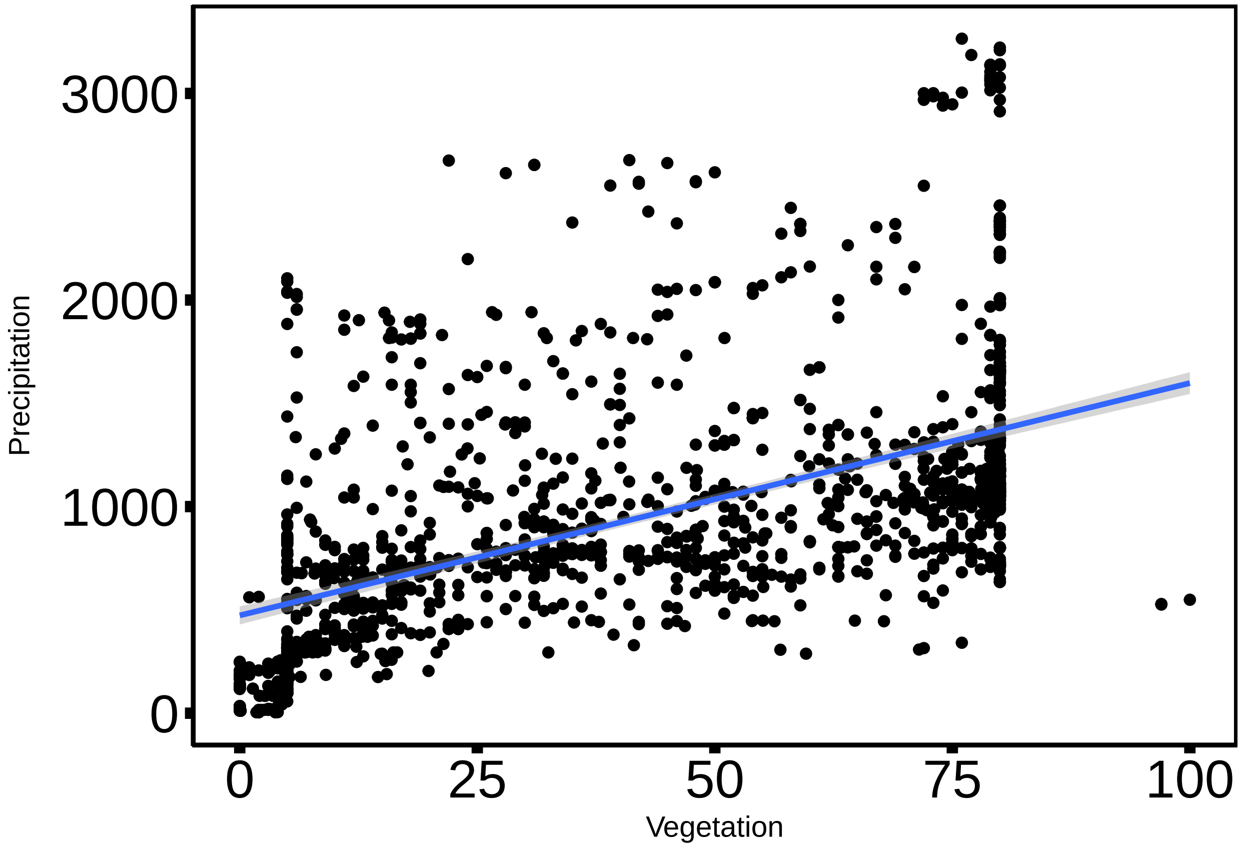

#Linear regression: do precip and vegcorrelate?

```
regression.m6<-lm(Precipitation ~ Vegetation, data=nydata)
plot(regression.m6)
```

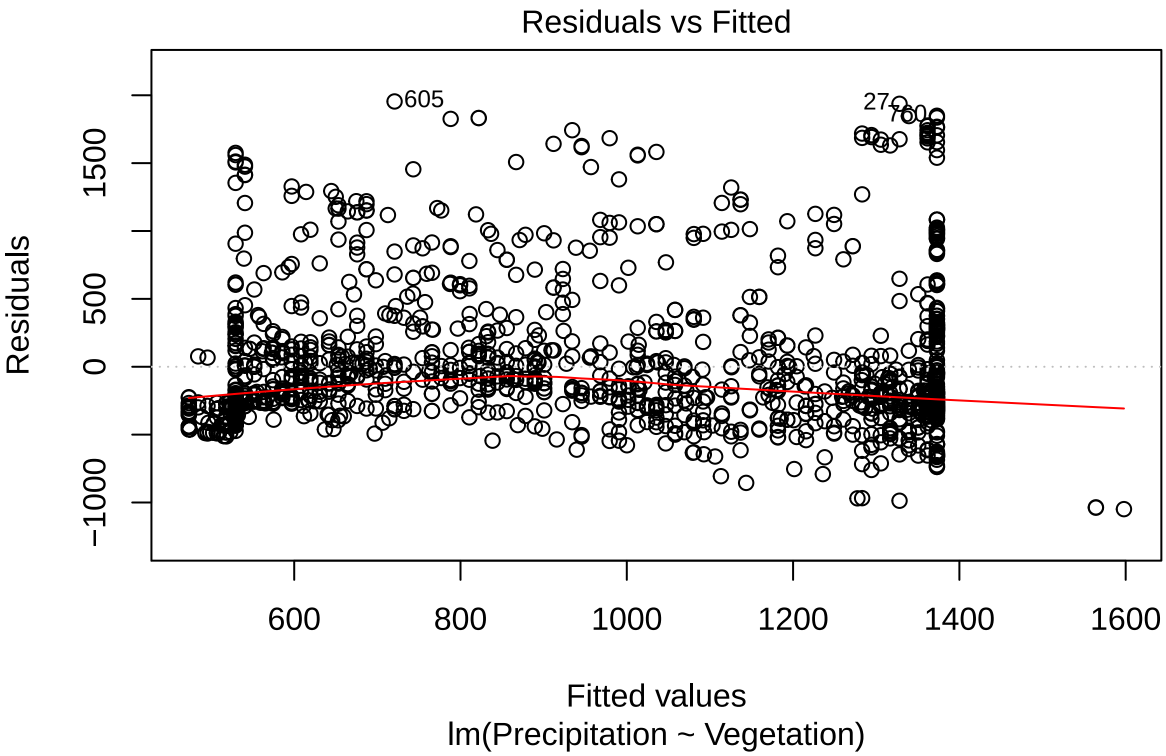

Residuals vs Fitted
605
27
760
Residuals
−1000
0
500
1500
600
800
1000
1200
1400
1600
Fitted values
lm(Precipitation ~ Vegetation)

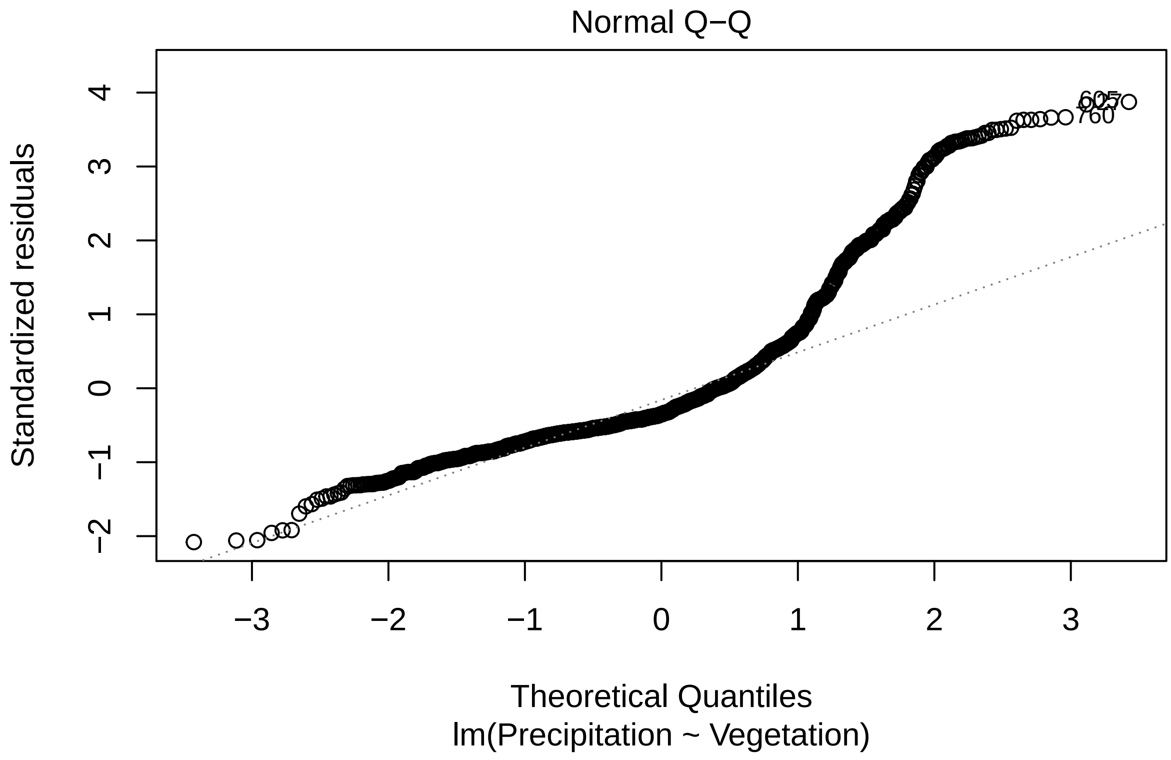
Normal Q−Q
Standardized residuals
Theoretical Quantiles
lm(Precipitation ~ Vegetation)
605
527
760
−3
−2
−1
0
1
2
3
4

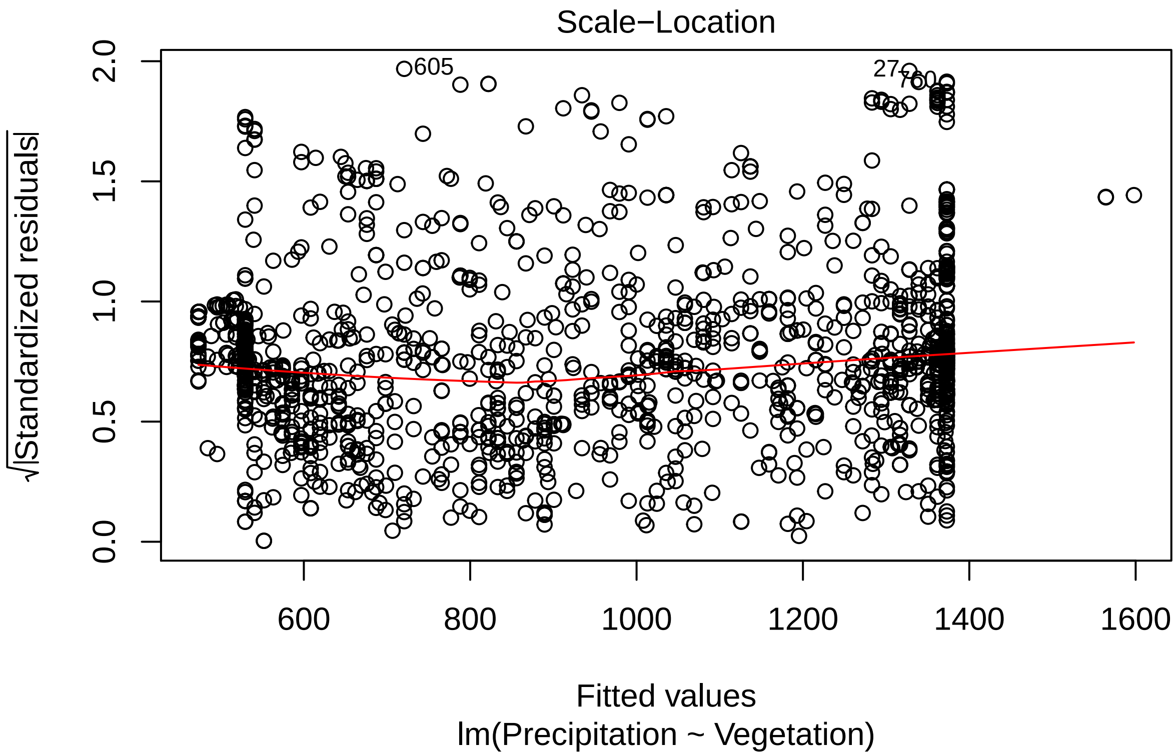

Scale−Location
605
27
760
2.0
1.5
1.0
0.5
0.0
√|Standardized residuals|
600
800
1000
1200
1400
1600
Fitted values
lm(Precipitation ~ Vegetation)

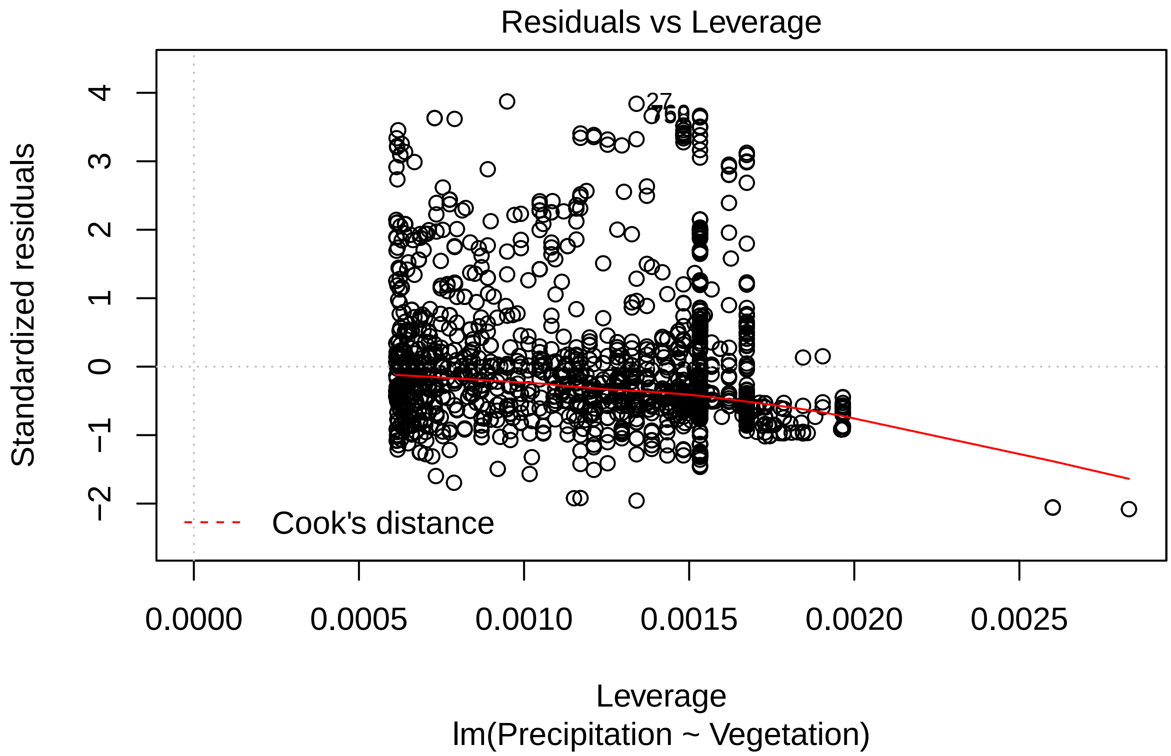


```r
summary(regression.m6)
```

```
##
## Call:
## lm(formula = Precipitation ~ Vegetation, data = nydata)
##
## Residuals:
##     Min      1Q  Median      3Q     Max
## -1048.8  -299.2  -176.5   139.6  1954.3
##
## Coefficients:
##             Estimate Std. Error t value Pr(>|t|)
## (Intercept) 473.2666    22.3767   21.15   <2e-16 ***
## Vegetation   11.2466     0.4234   26.57   <2e-16 ***
## ---
## Signif. codes:  0 '***' 0.001 '**' 0.01 '*' 0.05 '.' 0.1 ' ' 1
##
## Residual standard error: 504.7 on 1629 degrees of freedom
## Multiple R-squared:  0.3023, Adjusted R-squared:  0.3018
## F-statistic: 705.7 on 1 and 1629 DF,  p-value: < 2.2e-16
```

```r
regression.m7<-lm(Slope ~ Precipitation, data=nydata)
plot(regression.m7)
```

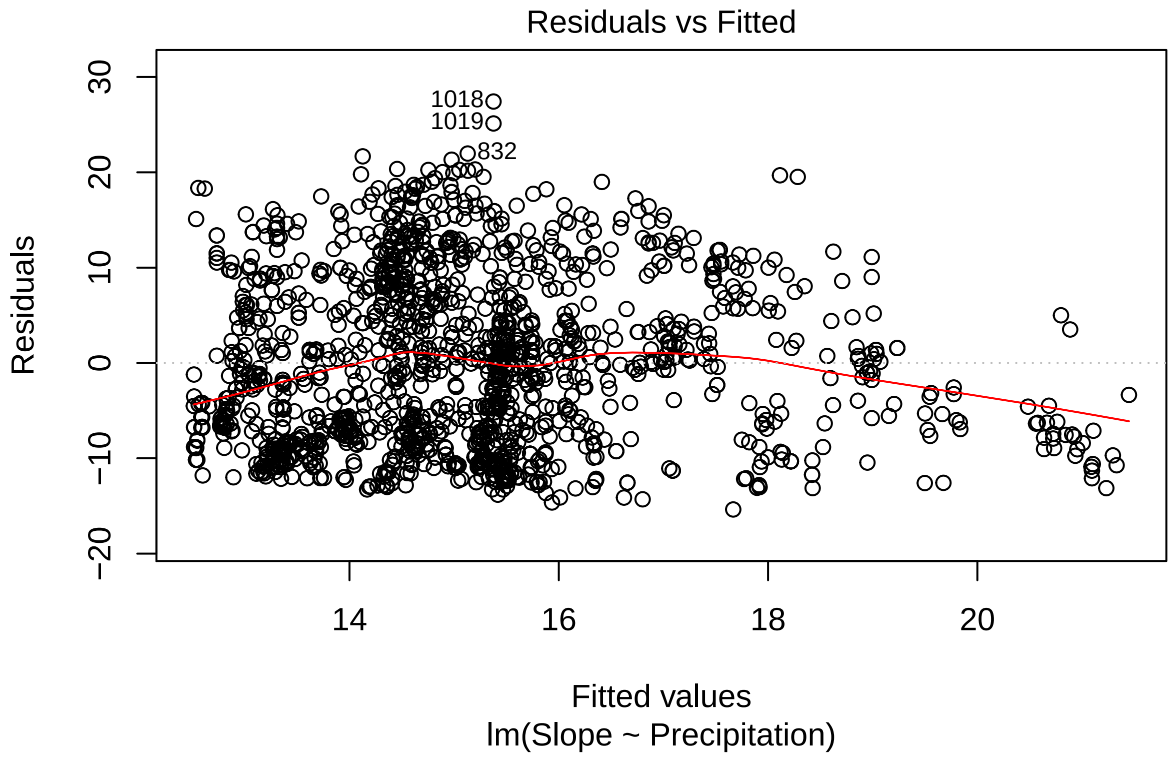
Residuals vs Fitted
Residuals
30
20
10
0
−10
−20
1018
1019
832
14
16
18
20
Fitted values
lm(Slope ~ Precipitation)

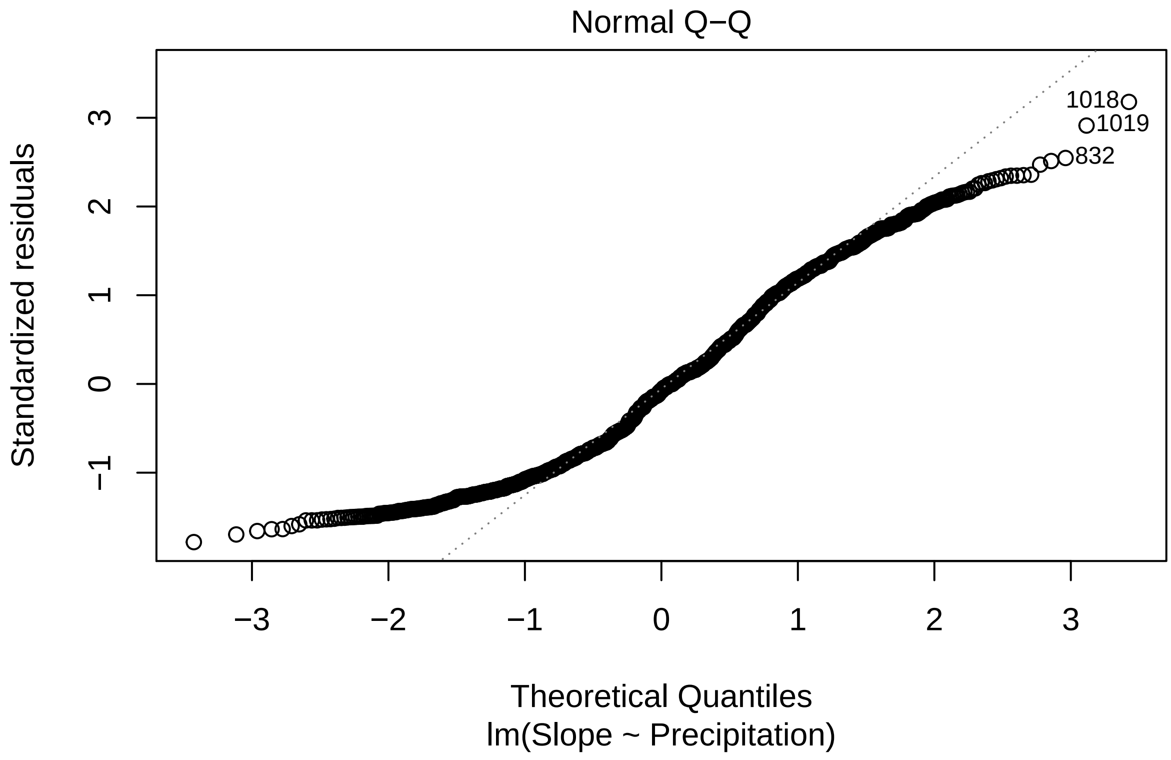
Normal Q−Q
Standardized residuals
Theoretical Quantiles
lm(Slope ~ Precipitation)
1018
1019
832
−1
0
1
2
3
−3
−2
−1
0
1
2
3

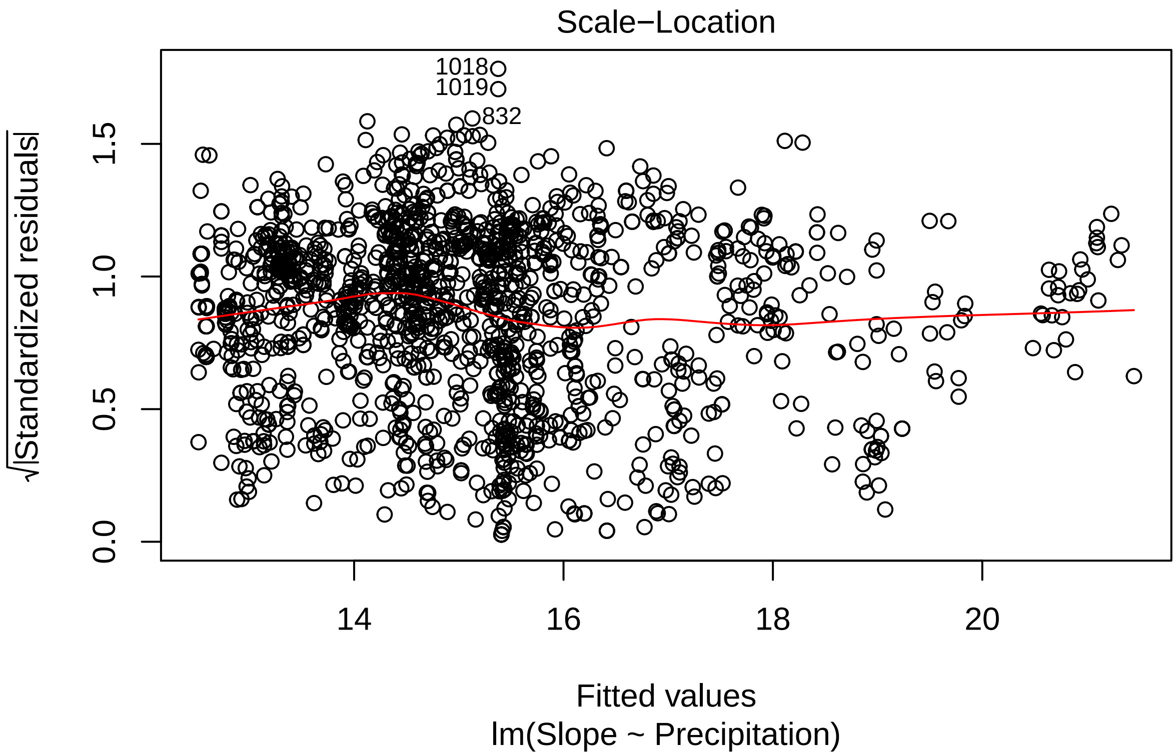
Scale−Location
1018
1019
832
√|Standardized residuals|
0.0
0.5
1.0
1.5
14
16
18
20
Fitted values
lm(Slope ~ Precipitation)

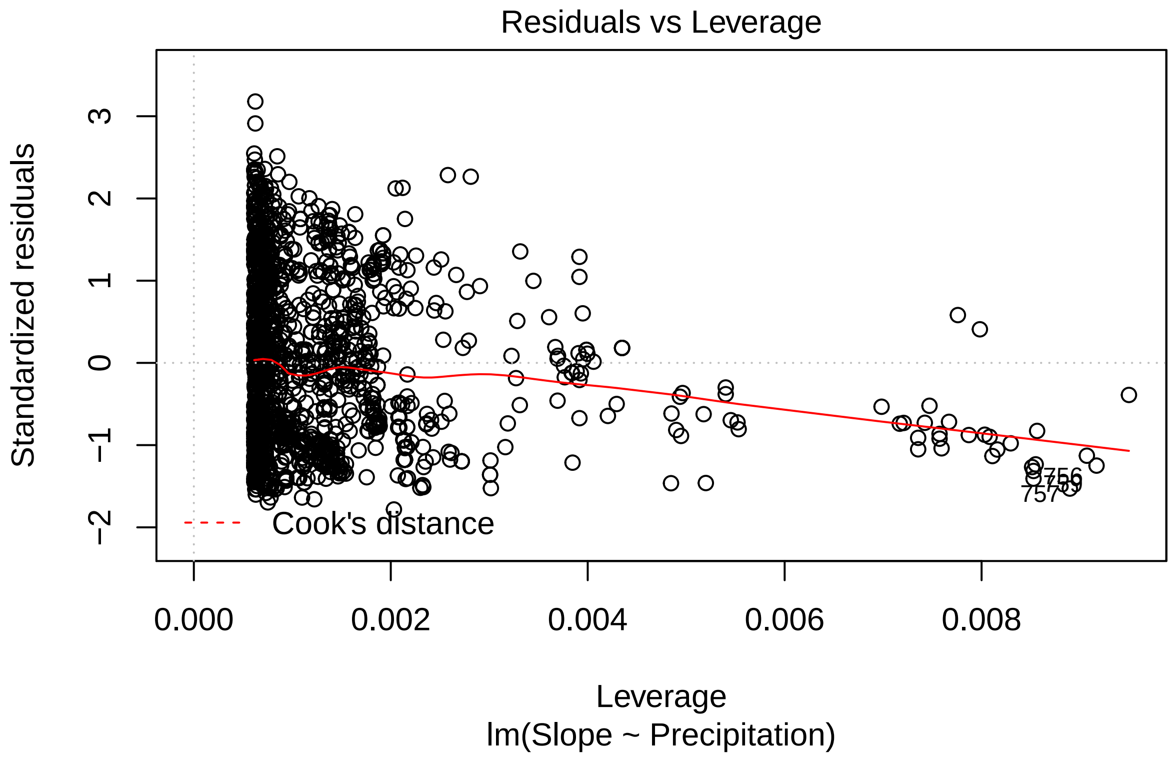


```
summary(regression.m7)
```

```
##
## Call:
## lm(formula = Slope ~ Precipitation, data = nydata)
##
## Residuals:
##      Min       1Q   Median       3Q      Max
## -15.3670  -7.4342  -0.6601   6.4827  27.4237
##
## Coefficients:
##                Estimate Std. Error t value Pr(>|t|)
## (Intercept)   1.250e+01  4.032e-01  31.006  < 2e-16 ***
## Precipitation 2.740e-03  3.538e-04   7.744 1.68e-14 ***
## ---
## Signif. codes:  0 '***' 0.001 '**' 0.01 '*' 0.05 '.' 0.1 ' ' 1
##
## Residual standard error: 8.63 on 1629 degrees of freedom
## Multiple R-squared:  0.03551,    Adjusted R-squared:  0.03492
## F-statistic: 59.97 on 1 and 1629 DF,  p-value: 1.68e-14
```

Use citation as random effect

```
glob.all = read.csv(file.choose())
glob.all.complete = glob.all[!is.na(glob.all$Vegetation),]
```

```r
str(glob.all)
```

```
## 'data.frame':    1789 obs. of  8 variables:
##  $ Study.number : int  1 1 1 1 1 1 1 1 1 1 ...
##  $ Citation     : Factor w/ 89 levels "Abbuhl et al. (2010)",..: 1 1 1 1 1 1 1 1 1 1 ...
##  $ Sample.ID    : Factor w/ 1633 levels " Piu11"," SH-32 ",..: 1132 195 1136 1134 1135 1137 1138 113
##  $ Slope        : num  10.8 12.4 14.9 19.3 18.6 19.2 20.8 21.4 21.1 20.1 ...
##  $ RockType     : Factor w/ 6 levels ""," Mixed","Igneous",..: 5 5 5 5 5 5 5 5 5 5 ...
##  $ Precipitation: num  338 378 429 510 626 639 716 743 763 815 ...
##  $ Vegetation   : num  8 8 9 10 9 9 9 13 13 9 ...
##  $ Erosion      : num  15.9 11.1 45.6 27.7 64.9 ...
```

```r
library(nlme)
```

```
##
## Attaching package: 'nlme'

## The following object is masked from 'package:dplyr':
##
##     collapse

## The following object is masked from 'package:lme4':
##
##     lmList
```

Improvemnent in model for precipitation + vegetation

```r
lme3p = lm(log(Erosion) ~  poly(Precipitation, 3) + poly (Vegetation, 2),
              data =glob.all.complete)
```

R2 value of model

```r
library(MuMIn)
r.squaredLR(lme3p)
```

```
## [1] 0.1831283
## attr(,"adj.r.squared")
## [1] 0.1872672
```

Improvement by addint Slope+precipitation+ vegetation

```r
lme3pp = lm(log(Erosion) ~  Slope + poly(Precipitation, 3) + poly(Vegetation, 2),
              data =glob.all.complete)
library(MuMIn)
r.squaredLR(lme3pp)
```

```
## [1] 0.3939928
## attr(,"adj.r.squared")
## [1] 0.4028975
```

```r
summary(lme3pp)
```

```
##
## Call:
## lm(formula = log(Erosion) ~ Slope + poly(Precipitation, 3) +
##     poly(Vegetation, 2), data = glob.all.complete)
##
## Residuals:
##     Min      1Q  Median      3Q     Max
## -5.6522 -0.7913 -0.0053  0.8331  4.3113
##
## Coefficients:
##                          Estimate Std. Error t value Pr(>|t|)
## (Intercept)              2.553771   0.065211  39.161  < 2e-16 ***
## Slope                    0.089645   0.003771  23.771  < 2e-16 ***
## poly(Precipitation, 3)1  13.585700   1.723171   7.884 5.76e-15 ***
## poly(Precipitation, 3)2  -8.026773   1.591010  -5.045 5.04e-07 ***
## poly(Precipitation, 3)3  12.968986   1.393732   9.305  < 2e-16 ***
## poly(Vegetation, 2)1    -22.076652   1.887597 -11.696  < 2e-16 ***
## poly(Vegetation, 2)2     -7.330610   1.507246  -4.864 1.26e-06 ***
## ---
## Signif. codes:  0 '***' 0.001 '**' 0.01 '*' 0.05 '.' 0.1 ' ' 1
##
## Residual standard error: 1.27 on 1624 degrees of freedom
## Multiple R-squared:  0.394,  Adjusted R-squared:  0.3918
## F-statistic:   176 on 6 and 1624 DF,  p-value: < 2.2e-16
```

maximum likelihood fits for model comparison

```r
lme2a = lme(log(Erosion) ~  Slope + poly(Vegetation,2) + poly(Precipitation, 2),
           method="ML", random = ~1|Citation, data = glob.all.complete)
summary(lme2a)
```

```
## Linear mixed-effects model fit by maximum likelihood
##  Data: glob.all.complete
##        AIC     BIC    logLik
##   3514.235 3557.41 -1749.117
##
## Random effects:
##  Formula: ~1 | Citation
##         (Intercept)  Residual
## StdDev:    1.318831 0.6399108
##
## Fixed effects: log(Erosion) ~ Slope + poly(Vegetation, 2) + poly(Precipitation,      2)
##                             Value Std.Error   DF   t-value p-value
## (Intercept)              2.982093 0.1586054 1545 18.801969  0.0000
## Slope                    0.063651 0.0033328 1545 19.098523  0.0000
## poly(Vegetation, 2)1    -2.232718 1.3809897 1545 -1.616752  0.1061
## poly(Vegetation, 2)2    -3.797537 0.9845335 1545 -3.857195  0.0001
## poly(Precipitation, 2)1  6.238972 2.0052657 1545  3.111294  0.0019
## poly(Precipitation, 2)2 -5.794004 2.2379151 1545 -2.589019  0.0097
##  Correlation:
##                         (Intr) Slope  p(V,2)1 p(V,2)2 p(P,2)1
```

```
## Slope                      -0.312
## poly(Vegetation, 2)1        0.071 -0.187
## poly(Vegetation, 2)2       -0.032  0.100 -0.233
## poly(Precipitation, 2)1    0.041 -0.137 -0.189   0.034
## poly(Precipitation, 2)2 -0.118 -0.029  0.266  -0.185  -0.028
##
## Standardized Within-Group Residuals:
##         Min          Q1         Med          Q3         Max
## -5.13279248 -0.54032155  0.02167082  0.55967625  4.18226353
##
## Number of Observations: 1631
## Number of Groups: 81
```

```
lme3a = lme(log(Erosion) ~  Slope + poly(Vegetation,2) + poly(Precipitation, 3),
            method="ML", random = ~1|Citation, data = glob.all.complete)
summary(lme3a)
```

```
## Linear mixed-effects model fit by maximum likelihood
##  Data: glob.all.complete
##        AIC      BIC    logLik
##   3506.107 3554.679 -1744.053
##
## Random effects:
##  Formula: ~1 | Citation
##         (Intercept) Residual
## StdDev:    1.277552 0.638853
##
## Fixed effects: log(Erosion) ~ Slope + poly(Vegetation, 2) + poly(Precipitation,      3)
##                              Value Std.Error   DF   t-value p-value
## (Intercept)               2.973089 0.1543598 1544 19.260776  0.0000
## Slope                     0.064336 0.0033311 1544 19.313476  0.0000
## poly(Vegetation, 2)1     -3.288882 1.4159441 1544 -2.322748  0.0203
## poly(Vegetation, 2)2     -3.214717 0.9997377 1544 -3.215560  0.0013
## poly(Precipitation, 3)1  8.955289 2.1634181 1544  4.139417  0.0000
## poly(Precipitation, 3)2 -5.866888 2.2214915 1544 -2.640968  0.0084
## poly(Precipitation, 3)3  5.569072 1.7394381 1544  3.201650  0.0014
##  Correlation:
##                         (Intr) Slope  p(V,2)1 p(V,2)2 p(P,3)1 p(P,3)2
## Slope                   -0.321
## poly(Vegetation, 2)1     0.075 -0.194
## poly(Vegetation, 2)2    -0.036  0.109 -0.265
## poly(Precipitation, 3)1  0.032 -0.103 -0.262   0.104
## poly(Precipitation, 3)2 -0.120 -0.028  0.259  -0.183  -0.031
## poly(Precipitation, 3)3 -0.017  0.058 -0.230   0.184   0.388  -0.005
##
## Standardized Within-Group Residuals:
##          Min           Q1          Med           Q3          Max
## -5.206163840 -0.552850519  0.008293124  0.549472635  4.170585799
##
## Number of Observations: 1631
## Number of Groups: 81
```

```
lme4a = lme(log(Erosion) ~  Slope + poly(Vegetation,2) + poly(Precipitation, 4),
            method="ML", random = ~1|Citation, data = glob.all.complete)
summary(lme4a)
```

```
## Linear mixed-effects model fit by maximum likelihood
##  Data: glob.all.complete
##        AIC      BIC    logLik
##   3499.409 3553.379 -1739.705
##
## Random effects:
##  Formula: ~1 | Citation
##         (Intercept)  Residual
## StdDev:    1.278939 0.6370313
##
## Fixed effects: log(Erosion) ~ Slope + poly(Vegetation, 2) + poly(Precipitation,      4)
##                               Value Std.Error   DF   t-value p-value
## (Intercept)                3.000931 0.1547759 1543 19.388876  0.0000
## Slope                      0.063564 0.0033330 1543 19.070998  0.0000
## poly(Vegetation, 2)1      -2.990806 1.4159748 1543 -2.112189  0.0348
## poly(Vegetation, 2)2      -3.072036 0.9984301 1543 -3.076867  0.0021
## poly(Precipitation, 4)1  8.944013 2.1597292 1543  4.141266  0.0000
## poly(Precipitation, 4)2 -7.163302 2.2614720 1543 -3.167540  0.0016
## poly(Precipitation, 4)3  5.503288 1.7362207 1543  3.169694  0.0016
## poly(Precipitation, 4)4 -3.897136 1.3227742 1543 -2.946184  0.0033
##  Correlation:
##                         (Intr) Slope  p(V,2)1 p(V,2)2 p(P,4)1 p(P,4)2
## Slope                   -0.323
## poly(Vegetation, 2)1     0.079 -0.199
## poly(Vegetation, 2)2    -0.032  0.104 -0.260
## poly(Precipitation, 4)1  0.032 -0.103 -0.261   0.104
## poly(Precipitation, 4)2 -0.129 -0.013  0.240  -0.189  -0.030
## poly(Precipitation, 4)3 -0.018  0.058 -0.230   0.183   0.388  -0.002
## poly(Precipitation, 4)4 -0.061  0.077 -0.070  -0.049   0.001   0.196
##                         p(P,4)3
## Slope
## poly(Vegetation, 2)1
## poly(Vegetation, 2)2
## poly(Precipitation, 4)1
## poly(Precipitation, 4)2
## poly(Precipitation, 4)3
## poly(Precipitation, 4)4  0.011
##
## Standardized Within-Group Residuals:
##         Min          Q1         Med          Q3         Max
## -5.31628568 -0.54442487  0.01350764  0.55310783  4.18505556
##
## Number of Observations: 1631
## Number of Groups: 81
```

```r
lme5a = lme(log(Erosion) ~  Slope + poly(Vegetation,2) + poly(Precipitation, 5),
            method="ML", random = ~1|Citation, data = glob.all.complete)
summary(lme5a)
```

```
## Linear mixed-effects model fit by maximum likelihood
##  Data: glob.all.complete
##        AIC      BIC    logLik
##   3499.023 3558.389 -1738.512
##
## Random effects:
```

```
##  Formula: ~1 | Citation
##         (Intercept)  Residual
## StdDev:    1.271842 0.6367208
##
## Fixed effects: log(Erosion) ~ Slope + poly(Vegetation, 2) + poly(Precipitation,      5)
##                              Value Std.Error   DF   t-value p-value
## (Intercept)               3.003097 0.1540844 1542 19.489944  0.0000
## Slope                     0.063659 0.0033324 1542 19.103030  0.0000
## poly(Vegetation, 2)1    -2.630610 1.4352916 1542 -1.832806  0.0670
## poly(Vegetation, 2)2    -3.034449 0.9984713 1542 -3.039095  0.0024
## poly(Precipitation, 5)1  8.915261 2.1571100 1542  4.132966  0.0000
## poly(Precipitation, 5)2 -7.322042 2.2606497 1542 -3.238911  0.0012
## poly(Precipitation, 5)3  5.500299 1.7345160 1542  3.171086  0.0015
## poly(Precipitation, 5)4 -4.403993 1.3625733 1542 -3.232114  0.0013
## poly(Precipitation, 5)5  1.681510 1.0902895 1542  1.542260  0.1232
##  Correlation:
##                         (Intr) Slope  p(V,2)1 p(V,2)2 p(P,5)1 p(P,5)2
## Slope                   -0.324
## poly(Vegetation, 2)1     0.080 -0.193
## poly(Vegetation, 2)2    -0.032  0.105 -0.253
## poly(Precipitation, 5)1  0.032 -0.103 -0.259   0.103
## poly(Precipitation, 5)2 -0.130 -0.013  0.229  -0.190  -0.030
## poly(Precipitation, 5)3 -0.018  0.058 -0.228   0.183   0.388  -0.002
## poly(Precipitation, 5)4 -0.062  0.071 -0.107  -0.053   0.004   0.200
## poly(Precipitation, 5)5  0.010  0.015  0.165   0.024  -0.010  -0.043
##                         p(P,5)3 p(P,5)4
## Slope
## poly(Vegetation, 2)1
## poly(Vegetation, 2)2
## poly(Precipitation, 5)1
## poly(Precipitation, 5)2
## poly(Precipitation, 5)3
## poly(Precipitation, 5)4  0.012
## poly(Precipitation, 5)5 -0.005  -0.242
##
## Standardized Within-Group Residuals:
##         Min          Q1         Med          Q3         Max
## -5.35518367 -0.54625242  0.01446509  0.55111692  4.19933332
##
## Number of Observations: 1631
## Number of Groups: 81
```

```r
lme6a = lme(log(Erosion) ~  Slope + poly(Vegetation,2) + poly(Precipitation, 6),
            method="ML", random = ~1|Citation, data = glob.all.complete)
summary(lme6a)
```

```
## Linear mixed-effects model fit by maximum likelihood
##  Data: glob.all.complete
##        AIC     BIC    logLik
##   3498.077 3562.84 -1737.038
##
## Random effects:
##  Formula: ~1 | Citation
##         (Intercept) Residual
## StdDev:    1.284052  0.63581
```

```
##
## Fixed effects: log(Erosion) ~ Slope + poly(Vegetation, 2) + poly(Precipitation,      6)
##                              Value Std.Error   DF   t-value p-value
## (Intercept)               3.015692 0.1555320 1541 19.389528  0.0000
## Slope                     0.063468 0.0033309 1541 19.054408  0.0000
## poly(Vegetation, 2)1     -2.685224 1.4344993 1541 -1.871889  0.0614
## poly(Vegetation, 2)2     -3.097306 0.9981025 1541 -3.103195  0.0019
## poly(Precipitation, 6)1  8.513181 2.1718333 1541  3.919813  0.0001
## poly(Precipitation, 6)2 -7.868523 2.2870974 1541 -3.440397  0.0006
## poly(Precipitation, 6)3  4.861471 1.7724358 1541  2.742819  0.0062
## poly(Precipitation, 6)4 -4.525387 1.3639585 1541 -3.317833  0.0009
## poly(Precipitation, 6)5  0.711235 1.2258205 1541  0.580211  0.5619
## poly(Precipitation, 6)6  1.895765 1.1041206 1541  1.716991  0.0862
##  Correlation:
##                         (Intr) Slope  p(V,2)1 p(V,2)2 p(P,6)1 p(P,6)2
## Slope                   -0.322
## poly(Vegetation, 2)1     0.078 -0.193
## poly(Vegetation, 2)2    -0.034  0.106 -0.252
## poly(Precipitation, 6)1  0.026 -0.099 -0.254   0.106
## poly(Precipitation, 6)2 -0.134 -0.009  0.230  -0.183  -0.013
## poly(Precipitation, 6)3 -0.027  0.062 -0.218   0.186   0.400   0.028
## poly(Precipitation, 6)4 -0.063  0.073 -0.106  -0.051   0.009   0.206
## poly(Precipitation, 6)5 -0.013  0.027  0.159   0.037   0.040   0.027
## poly(Precipitation, 6)6  0.046 -0.028 -0.026  -0.035  -0.105  -0.143
##                         p(P,6)3 p(P,6)4 p(P,6)5
## Slope
## poly(Vegetation, 2)1
## poly(Vegetation, 2)2
## poly(Precipitation, 6)1
## poly(Precipitation, 6)2
## poly(Precipitation, 6)3
## poly(Precipitation, 6)4  0.022
## poly(Precipitation, 6)5  0.089  -0.192
## poly(Precipitation, 6)6 -0.203  -0.051  -0.458
##
## Standardized Within-Group Residuals:
##        Min         Q1        Med         Q3        Max
## -5.3410517 -0.5354982  0.0176847  0.5519878  4.1879335
##
## Number of Observations: 1631
## Number of Groups: 81
```

```r
lme7a = lme(log(Erosion) ~  Slope + poly(Vegetation,2) + poly(Precipitation, 7),
           method="ML", random = ~1|Citation, data = glob.all.complete)
summary(lme7a)
```

```
## Linear mixed-effects model fit by maximum likelihood
##  Data: glob.all.complete
##        AIC      BIC    logLik
##   3500.016 3570.176 -1737.008
##
## Random effects:
##  Formula: ~1 | Citation
##         (Intercept)  Residual
## StdDev:    1.283772 0.6358045
```

```
##
## Fixed effects: log(Erosion) ~ Slope + poly(Vegetation, 2) + poly(Precipitation,      7)
##                               Value Std.Error   DF   t-value p-value
## (Intercept)                3.013979 0.1557045 1540 19.357052  0.0000
## Slope                      0.063526 0.0033401 1540 19.019409  0.0000
## poly(Vegetation, 2)1      -2.668471 1.4365698 1540 -1.857530  0.0634
## poly(Vegetation, 2)2      -3.085548 0.9995285 1540 -3.087004  0.0021
## poly(Precipitation, 7)1  8.588042 2.1933515 1540  3.915489  0.0001
## poly(Precipitation, 7)2 -7.753682 2.3347587 1540 -3.320978  0.0009
## poly(Precipitation, 7)3  4.961704 1.8184570 1540  2.728524  0.0064
## poly(Precipitation, 7)4 -4.378545 1.4887024 1540 -2.941182  0.0033
## poly(Precipitation, 7)5  0.686918 1.2302150 1540  0.558372  0.5767
## poly(Precipitation, 7)6  1.980520 1.1570287 1540  1.711729  0.0871
## poly(Precipitation, 7)7 -0.291437 1.1827957 1540 -0.246397  0.8054
##  Correlation:
##                         (Intr) Slope  p(V,2)1 p(V,2)2 p(P,7)1 p(P,7)2
## Slope                   -0.324
## poly(Vegetation, 2)1     0.075 -0.189
## poly(Vegetation, 2)2    -0.036  0.108 -0.249
## poly(Precipitation, 7)1  0.020 -0.089 -0.245   0.112
## poly(Precipitation, 7)2 -0.141  0.005  0.235  -0.169   0.015
## poly(Precipitation, 7)3 -0.036  0.076 -0.201   0.192   0.417   0.071
## poly(Precipitation, 7)4 -0.076  0.094 -0.078  -0.028   0.063   0.265
## poly(Precipitation, 7)5 -0.009  0.021  0.154   0.033   0.028   0.010
## poly(Precipitation, 7)6  0.031 -0.006 -0.011  -0.019  -0.059  -0.074
## poly(Precipitation, 7)7  0.044 -0.070 -0.048  -0.048  -0.138  -0.200
##                         p(P,7)3 p(P,7)4 p(P,7)5 p(P,7)6
## Slope
## poly(Vegetation, 2)1
## poly(Vegetation, 2)2
## poly(Precipitation, 7)1
## poly(Precipitation, 7)2
## poly(Precipitation, 7)3
## poly(Precipitation, 7)4  0.109
## poly(Precipitation, 7)5  0.068  -0.208
## poly(Precipitation, 7)6 -0.122   0.075  -0.460
## poly(Precipitation, 7)7 -0.222  -0.400   0.081  -0.298
##
## Standardized Within-Group Residuals:
##         Min          Q1         Med          Q3         Max
## -5.34047578 -0.53809735  0.01588461  0.55610404  4.19804884
##
## Number of Observations: 1631
## Number of Groups: 81
```

```
lme8a = lme(log(Erosion) ~  Slope + poly(Vegetation,2) + poly(Precipitation, 8),
            method="ML", random = ~1|Citation, data = glob.all.complete)
summary(lme8a)
```

```
## Linear mixed-effects model fit by maximum likelihood
##  Data: glob.all.complete
##       AIC      BIC   logLik
##   3500.78 3576.337 -1736.39
##
## Random effects:
```

```
##  Formula: ~1 | Citation
##         (Intercept)  Residual
## StdDev:    1.271987 0.6358476
##
## Fixed effects: log(Erosion) ~ Slope + poly(Vegetation, 2) + poly(Precipitation,      8)
##                             Value Std.Error   DF   t-value p-value
## (Intercept)              3.012768 0.1545321 1539 19.496068  0.0000
## Slope                    0.063250 0.0033517 1539 18.871124  0.0000
## poly(Vegetation, 2)1    -2.610614 1.4380678 1539 -1.815362  0.0697
## poly(Vegetation, 2)2    -3.207482 1.0060211 1539 -3.188285  0.0015
## poly(Precipitation, 8)1  8.966202 2.2136023 1539  4.050502  0.0001
## poly(Precipitation, 8)2 -7.336575 2.3605734 1539 -3.107963  0.0019
## poly(Precipitation, 8)3  5.625871 1.9041336 1539  2.954557  0.0032
## poly(Precipitation, 8)4 -4.414495 1.4879011 1539 -2.966928  0.0031
## poly(Precipitation, 8)5  1.062673 1.2729317 1539  0.834823  0.4039
## poly(Precipitation, 8)6  1.561259 1.2138393 1539  1.286216  0.1986
## poly(Precipitation, 8)7  0.117409 1.2380576 1539  0.094833  0.9245
## poly(Precipitation, 8)8 -1.203535 1.0809846 1539 -1.113369  0.2657
##  Correlation:
##                         (Intr) Slope  p(V,2)1 p(V,2)2 p(P,8)1 p(P,8)2
## Slope                   -0.325
## poly(Vegetation, 2)1     0.076 -0.191
## poly(Vegetation, 2)2    -0.035  0.117 -0.251
## poly(Precipitation, 8)1  0.020 -0.099 -0.237   0.093
## poly(Precipitation, 8)2 -0.140 -0.008  0.238  -0.184   0.037
## poly(Precipitation, 8)3 -0.036  0.048 -0.180   0.148   0.436   0.114
## poly(Precipitation, 8)4 -0.076  0.096 -0.079  -0.025   0.058   0.256
## poly(Precipitation, 8)5 -0.010 -0.001  0.160   0.003   0.064   0.051
## poly(Precipitation, 8)6  0.031  0.019 -0.023   0.015  -0.100  -0.118
## poly(Precipitation, 8)7  0.041 -0.091 -0.033  -0.078  -0.085  -0.139
## poly(Precipitation, 8)8  0.005  0.081 -0.041   0.111  -0.148  -0.161
##                         p(P,8)3 p(P,8)4 p(P,8)5 p(P,8)6 p(P,8)7
## Slope
## poly(Vegetation, 2)1
## poly(Vegetation, 2)2
## poly(Precipitation, 8)1
## poly(Precipitation, 8)2
## poly(Precipitation, 8)3
## poly(Precipitation, 8)4  0.096
## poly(Precipitation, 8)5  0.140  -0.207
## poly(Precipitation, 8)6 -0.202   0.079  -0.501
## poly(Precipitation, 8)7 -0.112  -0.388   0.151  -0.362
## poly(Precipitation, 8)8 -0.301   0.024  -0.258   0.303  -0.297
##
## Standardized Within-Group Residuals:
##         Min          Q1         Med          Q3         Max
## -5.31825900 -0.54641218  0.01656766  0.56082429  4.18388449
##
## Number of Observations: 1631
## Number of Groups: 81
```

```
anova(lme2a, lme3a,lme4a,lme5a,lme6a,lme7a, lme8a)
```

```
##       Model df      AIC      BIC    logLik   Test   L.Ratio p-value
## lme2a     1  8 3514.235 3557.410 -1749.117
```

```
## lme3a     2  9 3506.107 3554.679 -1744.053 1 vs 2 10.127946  0.0015
## lme4a     3 10 3499.409 3553.379 -1739.705 2 vs 3  8.697321  0.0032
## lme5a     4 11 3499.023 3558.389 -1738.512 3 vs 4  2.386294  0.1224
## lme6a     5 12 3498.077 3562.840 -1737.038 4 vs 5  2.946309  0.0861
## lme7a     6 13 3500.016 3570.176 -1737.008 5 vs 6  0.061116  0.8047
## lme8a     7 14 3500.780 3576.337 -1736.390 6 vs 7  1.235923  0.2663
```

```
AIC(lme2a, lme3a,lme4a,lme5a,lme6a,lme7a, lme8a)
```

```
##       df      AIC
## lme2a  8 3514.235
## lme3a  9 3506.107
## lme4a 10 3499.409
## lme5a 11 3499.023
## lme6a 12 3498.077
## lme7a 13 3500.016
## lme8a 14 3500.780
```

Clearly stop at 3

refit order 3 polynomial with an REML fit

```
lme3.final = lme(log(Erosion) ~  Slope + poly(Vegetation,2) + poly(Precipitation, 3),
            method="ML", random = ~1|Citation, data = glob.all.complete)
summary(lme3.final)
```

```
## Linear mixed-effects model fit by maximum likelihood
##  Data: glob.all.complete
##        AIC      BIC    logLik
##   3506.107 3554.679 -1744.053
##
## Random effects:
##  Formula: ~1 | Citation
##         (Intercept) Residual
## StdDev:    1.277552 0.638853
##
## Fixed effects: log(Erosion) ~ Slope + poly(Vegetation, 2) + poly(Precipitation,      3)
##                             Value Std.Error   DF   t-value p-value
## (Intercept)              2.973089 0.1543598 1544 19.260776  0.0000
## Slope                    0.064336 0.0033311 1544 19.313476  0.0000
## poly(Vegetation, 2)1    -3.288882 1.4159441 1544 -2.322748  0.0203
## poly(Vegetation, 2)2    -3.214717 0.9997377 1544 -3.215560  0.0013
## poly(Precipitation, 3)1  8.955289 2.1634181 1544  4.139417  0.0000
## poly(Precipitation, 3)2 -5.866888 2.2214915 1544 -2.640968  0.0084
## poly(Precipitation, 3)3  5.569072 1.7394381 1544  3.201650  0.0014
##  Correlation:
##                         (Intr) Slope  p(V,2)1 p(V,2)2 p(P,3)1 p(P,3)2
## Slope                   -0.321
## poly(Vegetation, 2)1     0.075 -0.194
## poly(Vegetation, 2)2    -0.036  0.109 -0.265
## poly(Precipitation, 3)1  0.032 -0.103 -0.262   0.104
## poly(Precipitation, 3)2 -0.120 -0.028  0.259  -0.183  -0.031
## poly(Precipitation, 3)3 -0.017  0.058 -0.230   0.184   0.388  -0.005
```

```
##
## Standardized Within-Group Residuals:
##          Min           Q1          Med           Q3          Max
## -5.206163840 -0.552850519  0.008293124  0.549472635  4.170585799
##
## Number of Observations: 1631
## Number of Groups: 81
```

Check r-squared values

```r
library(MuMIn)
r.squaredLR(lme3a)
```

```
## [1] 0.8124054
## attr(,"adj.r.squared")
## [1] 0.8307667
```

```r
r.squaredLR(lme3.final)
```

```
## [1] 0.8124054
## attr(,"adj.r.squared")
## [1] 0.8307667
```

```r
r.squaredGLMM(lme3.final)
```

```
##       R2m       R2c
## 0.1939938 0.8387677
```

Good overall model! but note that the fixed factors account for less of the variance than the random effect

How much of the variance does the precipitation polynomial account for?

Run model without it and compare

```r
lmeNoPrecip = lme(log(Erosion) ~  Slope + poly(Vegetation,2),
                  method="ML", random = ~1|Citation, data = glob.all.complete)
r.squaredLR(lmeNoPrecip)
```

```
## [1] 0.8093964
## attr(,"adj.r.squared")
## [1] 0.8276897
```

```r
r.squaredGLMM(lmeNoPrecip)
```

```
##       R2m       R2c
## 0.1389556 0.8426419
```

Not much overall, its taken up by the random effect – but improves the fixed effects by

about 40% (from about 0.13 to 0.19)

generate predictions for precipitation range and median values of slope and tree cover,

using a single citation with an erosion level close to average

Find the range of annual precipitation values

```
range(glob.all$Precipitation)
```

```
## [1]    3 3265
```

find the overal mean and citation means for log.erosion

```
glob.all$Erosion_log= log(glob.all$Erosion)
mean(glob.all$Erosion_log)  ## about 3.90
```

```
## [1] 3.835456
```

```
tapply(glob.all$Erosion_log, glob.all$Citation, mean) # Several have means very  close to the overall m
```

```
##         Abbuhl et al. (2010)        Acosta et al. (2015)
##                    3.9573536                   2.6411474
##          Belmont et al. 2007     Bierman and Caffee 2001
##                    5.2402029                   1.5937278
##          Bierman et al  2005          Bierman et al  2007
##                    4.4173538                   1.9655060
##         Bierman et al (2005)          Bierman et al 1998
##                    2.5122677                   1.0788654
##           Bierman et al 2001          Bierman et al 2009
##                    4.2329245                   2.8720743
##            Binnie et al 2006           Binnie et al 2008
##                    5.3051857                   5.6945818
##             Brown et al 1995            Brown et al 1998
##                    3.3196257                   3.8427232
##          Buechi et al.(2014)   Carretier, et al. (2013)
##                    4.6680739                   2.9118491
##          Chappell et al 2006            Clapp et al 2000
##                    4.1283158                   3.2496205
##             Clapp et al 2001            Clapp et al 2002
##                    4.2998318                   3.2112690
##                Codilean-2008               Codilean-2012
##                    2.1957997                   2.0718090
##               Cox et al 2009   Croke, J., et al. (2015)
##                    2.4313498                   2.5954528
##         Cyr and Granger 2008              Cyr et al 2010
##                    6.0113343                   7.1779379
##                 Delunel-2010          Delunel et al 2010
```

```
##                 6.5135880                 6.5299152
##             Densmore-2009        DiBiase et al 2009
##                 6.8095365                 5.5764461
##         Dirks et al. 2016              Duxbury 2008
##                 0.9611333                 2.2133581
##    Duxbury et. al. (2014)        Ferrier et al 2005
##                 2.1443398                 5.2597126
##      Finnegan et al 2008  Glotzbach et al.,(2014)
##                 6.9985906                 4.2827084
##         Godard et al 2010        Granger et al 1996
##                 6.4963007                 3.7885362
##       Guralnik et al 2010        Harkins et al 2010
##                 3.2630126                 4.1682283
##       Heimsath et al 1999       Heimsath et al 2001
##                 4.1722217                 3.5457116
##       Heimsath et al 2006       Heimsath et al 2009
##                 3.6682352                 2.6500852
##       Heimsath et al 2010          Henck et al 2011
##                 1.0148766                 4.2031732
##      Hewawasam et al 2003       Hippe et al. (2012)
##                 2.9475616                 1.9897589
##          Insel et al 2010       Kirchner et al 2001
##                 5.1471373                 4.1599293
##          Kober et al 2009      Larsen et al. (2014)
##                 3.0301949                 5.6336211
##         Matmon et al 2003                Meyer-2010
##                 3.1452479                 3.8959038
##           Meyer-2010 (a)      Miller et al. (2013)
##                 3.9321666                 2.5803732
##          Morel et al 2003        Nichols et al 2002
##                 3.4637000                 3.7245979
##    Nichols et al 2005 (a)    Nichols et al 2005 (b)
##                 3.4175567                 5.0201630
##        Nichols et al 2007    Nichols et al., (2014)
##                 2.8088001                 3.1617442
##               Norton-2010               Norton-2011
##                 6.0643034                 4.0174907
##         Norton et al 2007         Ouimet et al 2009
##                 5.8367961                 4.9674828
##              Palumbo-2011        Palumbo et al 2009
##                 4.9673191                 5.0456836
##           Perg et al 2003        Placzek et al 2010
##                 4.9920080                -0.3906133
##        Placzek et al 2014       Pumin et al. (2015)
##                -0.4229516                 1.5556485
##      Quigley et al 2007a      Quigley et al 2007b
##                 3.3297014                 2.4895713
##      Reinhardt et al 2007               Reuter 2005
##                 5.6493420                 2.7043103
##          Riebe et al 2000          Riebe et al 2003
##                 3.5980253                 4.0551525
##         Safran et al 2005       Schaller et al 2001
##                 5.5087866                 3.5633208
##               Scharf-2012          Stock et al 2009
```

```
##                  1.4881506                  4.7364289
##               Sullivan 2007          Tomkins et al 2007
##                  2.6863033                  3.1069095
##         Vanacker et al 2007 von Blanckenburg et al 2004
##                  2.2380392                  2.3520408
##               Wittmann-2011         Wittmann et al 2007
##                  2.5872572                  6.3180681
##         Wittmann et al 2009
##                  5.4021486
```

```
## Granger et al 1996, Brown et al 1998, Meyer-2010
```

Specify a regularly-spaced vector of precipitation levels covering the observed range

Put it into a new dataframe

The new dataframe will be used to predict erosion levels with constant values for Citation, slope & tree cover

```
Precipitation=seq(0, 3265, 5)  # has 654 values
newdata=data.frame(Precipitation)
```

use selected citation, making sure it has the right level number

```
newdata$Citation = "Granger et al 1996"
newdata$Citation = factor(newdata$Citation, levels=levels(glob.all.complete$Citation))
```

use median basin slope

```
newdata$Slope = median(glob.all.complete$Slope)
```

use median tree cover

```
newdata$Vegetation = median(glob.all.complete$Vegetation)
```

generate and plot predicted values

```
newdata$pred.erosion = predict(lme3.final, newdata=newdata)
with(newdata, plot(pred.erosion ~ Precipitation, type="l", ylim=c(-3,9)))
with(glob.all.complete, points(Precipitation, Erosion))
```

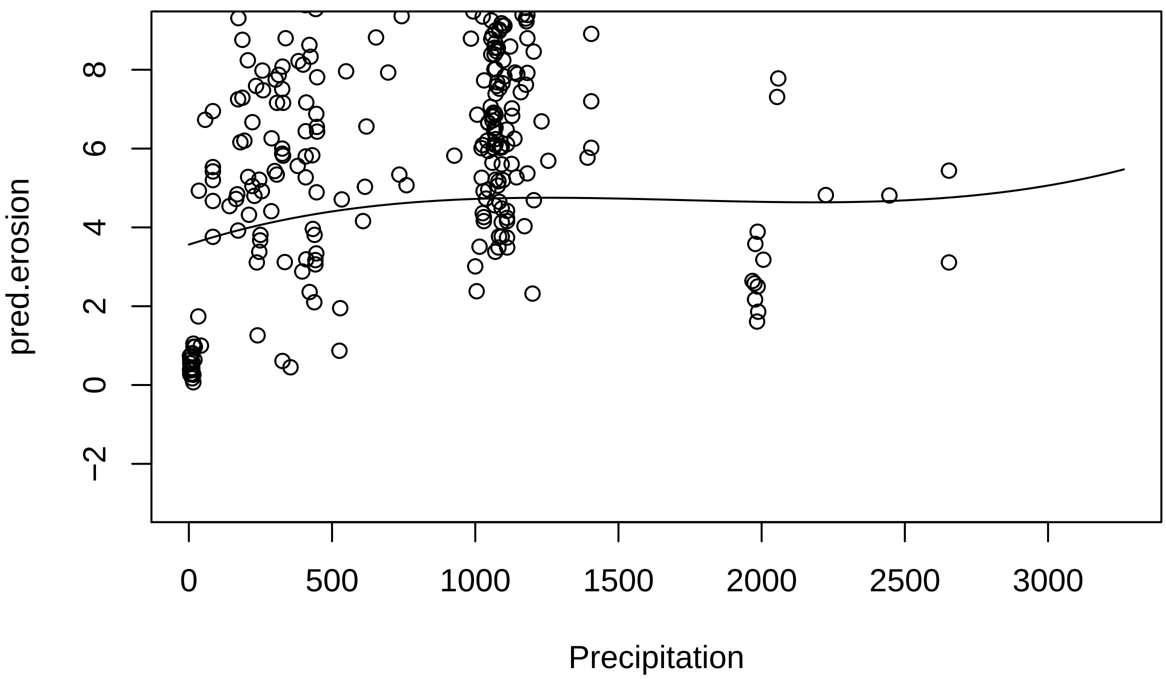

find inflexion points in the predicted sequence (to the nearest 5mm precipitation) (NB could do this more precisely by differentiating the

predictive equation)

NB because all the terms in the fitted model are additive, with no interaction terms, the inflection points will be the same regardless of the values of

the other covariates.

```
for (i in 2:653 )
{
  if (newdata$pred.erosion[i-1] <= newdata$pred.erosion[i]  && newdata$pred.erosion[i+1] <= newdata$pred
    cat("Maximum at ",newdata$Precipitation[i],"\n" )
  if (newdata$pred.erosion[i-1] >= newdata$pred.erosion[i]  && newdata$pred.erosion[i+1] >= newdata$pred
    cat("Minimum at ",newdata$Precipitation[i],"\n" )
}
```

```
## Maximum at  1250
## Minimum at  2180
```

finding inflection point of Precipitation

```
model.prec = lm(log(Erosion) ~  poly(Precipitation, 3),
             data = glob.all.complete)
model.veg = lm(log(Erosion)~  poly(Vegetation, 2),
             data = glob.all.complete)
```

trying New model (Only precip and erosion)

```
newdata$pred.erosion = predict(model.prec, newdata=newdata)
with(newdata, plot(pred.erosion ~ Precipitation, type="l", ylim=c(-3,9)))
with(glob.all.complete, points(Precipitation, Erosion))
```

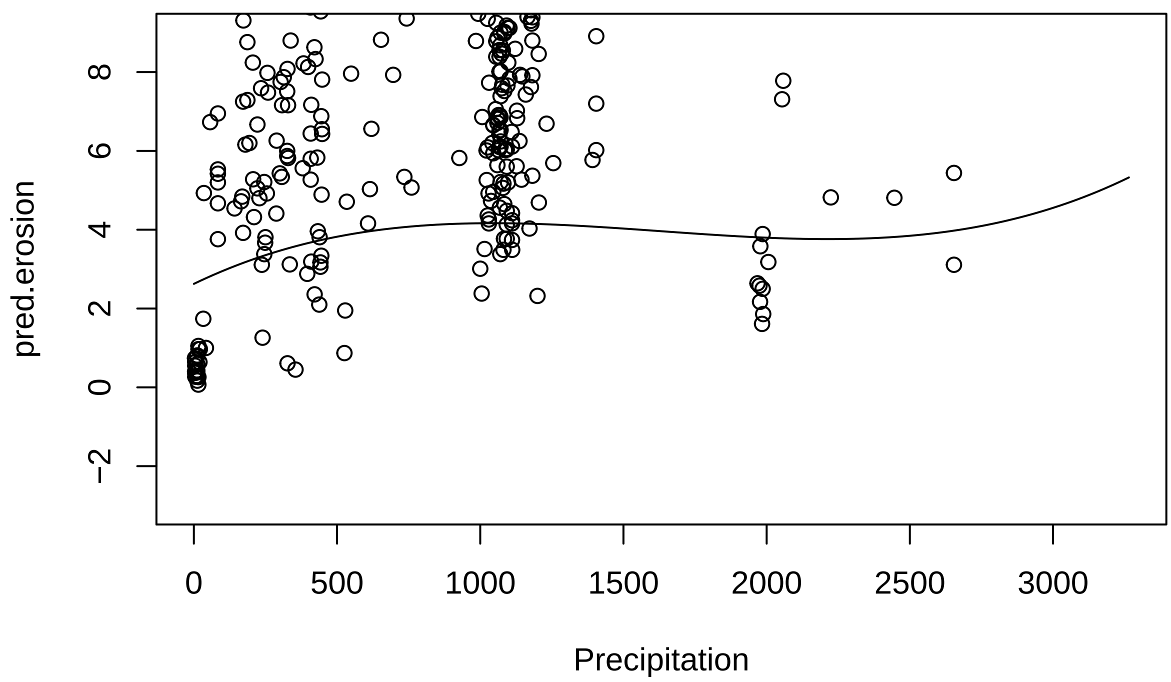

#Inflection point

```
for (i in 2:653 )
{
  if (newdata$pred.erosion[i-1] <= newdata$pred.erosion[i]  && newdata$pred.erosion[i+1] <= newdata$pre
    cat("Maximum at ",newdata$Precipitation[i],"\n" )
  if (newdata$pred.erosion[i-1] >= newdata$pred.erosion[i]  && newdata$pred.erosion[i+1] >= newdata$pre
    cat("Minimum at ",newdata$Precipitation[i],"\n" )
}
```

```
## Maximum at  1045
## Minimum at  2215
```

now for vegetation

```r
range(glob.all.complete$Vegetation)
```

```
## [1]   0 100
```

```r
Vegetation=seq(0, 100, 5)  # has 654 values
newdata=data.frame(Vegetation)
```

```r
newdata$pred.erosion = predict(model.veg, newdata=newdata)
with(newdata, plot(pred.erosion ~ Vegetation, type="l", ylim=c(-3,9)))
with(glob.all.complete, points(Vegetation, Erosion))
```

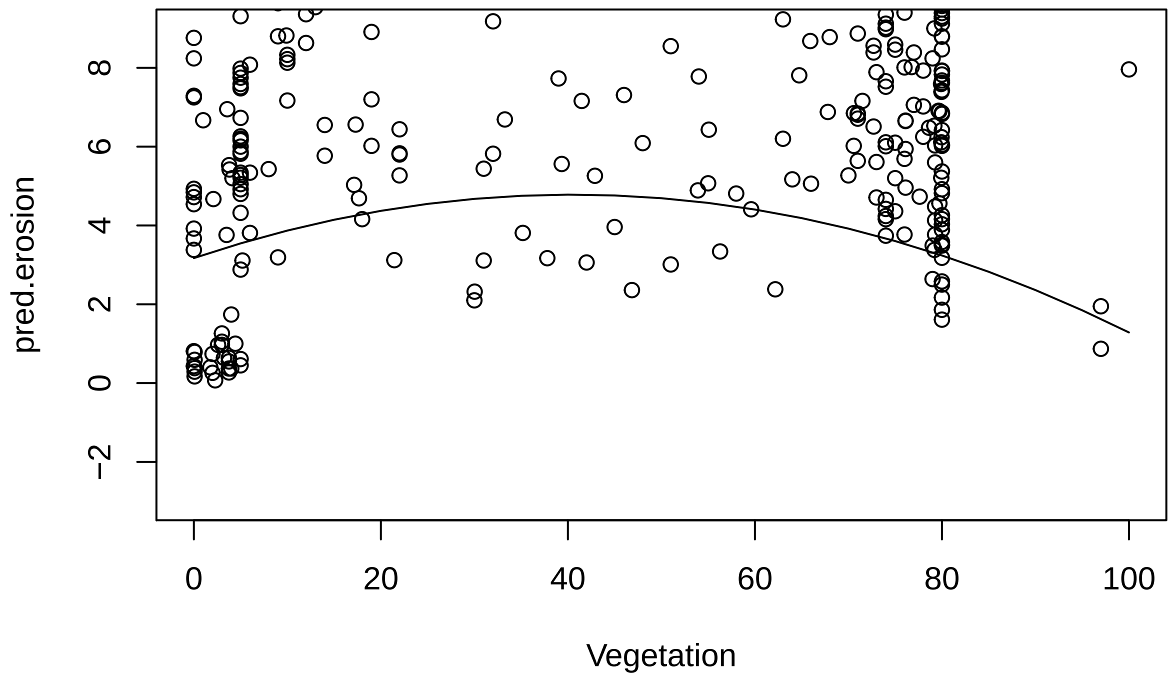


```r
for (i in 2:20 )
{
  if (newdata$pred.erosion[i-1] <= newdata$pred.erosion[i]  && newdata$pred.erosion[i+1] <= newdata$pred
    cat("Maximum at ",newdata$Vegetation[i],"\n" )
  if (newdata$pred.erosion[i-1] >= newdata$pred.erosion[i]  && newdata$pred.erosion[i+1] >= newdata$pred
    cat("Minimum at ",newdata$Vegetation[i],"\n" )
}
```

```
## Maximum at  40
```

Now for areas less than 11 degree in slope

```r
slopedata<-read.csv(file.choose())
```

erosion rate vs slope at areas <11 degree

```r
regression.A1<-lm(log(Erosion) ~ Slope, data=slopedata)
summary(regression.A1)
```

```
##
## Call:
## lm(formula = log(Erosion) ~ Slope, data = slopedata)
##
## Residuals:
##     Min      1Q  Median      3Q     Max
## -5.1259 -0.6805 -0.0088  0.7860  4.7266
##
## Coefficients:
##             Estimate Std. Error t value Pr(>|t|)
## (Intercept)  2.10900    0.12153  17.354  < 2e-16 ***
## Slope        0.15025    0.01856   8.098 2.48e-15 ***
## ---
## Signif. codes:  0 '***' 0.001 '**' 0.01 '*' 0.05 '.' 0.1 ' ' 1
##
## Residual standard error: 1.242 on 702 degrees of freedom
## Multiple R-squared:  0.08542,    Adjusted R-squared:  0.08412
## F-statistic: 65.57 on 1 and 702 DF,  p-value: 2.48e-15
```

erosion rate vs precipitation at areas <11 degree

```r
regression.A2<-lm(log(Erosion) ~ poly(Precipitation, 3), data=slopedata)
summary (regression.A2)
```

```
##
## Call:
## lm(formula = log(Erosion) ~ poly(Precipitation, 3), data = slopedata)
##
## Residuals:
##     Min      1Q  Median      3Q     Max
## -4.8787 -0.7841  0.0186  0.8131  4.2645
##
## Coefficients:
##                         Estimate Std. Error t value Pr(>|t|)
## (Intercept)              3.01717    0.04691  64.321  < 2e-16 ***
## poly(Precipitation, 3)1  3.35257    1.24462   2.694  0.00724 **
## poly(Precipitation, 3)2  0.25944    1.24462   0.208  0.83494
## poly(Precipitation, 3)3  9.38049    1.24462   7.537 1.49e-13 ***
## ---
## Signif. codes:  0 '***' 0.001 '**' 0.01 '*' 0.05 '.' 0.1 ' ' 1
##
## Residual standard error: 1.245 on 700 degrees of freedom
## Multiple R-squared:  0.08389,    Adjusted R-squared:  0.07997
## F-statistic: 21.37 on 3 and 700 DF,  p-value: 2.982e-13
```

```r
slopedata$Erosion_log<-log(slopedata$Erosion)
regression.A1<-lm(log(Erosion) ~ Slope, data=slopedata)
regression.A2<-lm(log(Erosion) ~ poly(Precipitation, 3), data=slopedata)
```

```r
summary(regression.A1)
```

```
##
## Call:
```

```
## lm(formula = log(Erosion) ~ Slope, data = slopedata)
##
## Residuals:
##     Min      1Q  Median      3Q     Max
## -5.1259 -0.6805 -0.0088  0.7860  4.7266
##
## Coefficients:
##             Estimate Std. Error t value Pr(>|t|)
## (Intercept)  2.10900    0.12153  17.354  < 2e-16 ***
## Slope        0.15025    0.01856   8.098 2.48e-15 ***
## ---
## Signif. codes:  0 '***' 0.001 '**' 0.01 '*' 0.05 '.' 0.1 ' ' 1
##
## Residual standard error: 1.242 on 702 degrees of freedom
## Multiple R-squared:  0.08542,    Adjusted R-squared:  0.08412
## F-statistic: 65.57 on 1 and 702 DF,  p-value: 2.48e-15
```

```
summary(regression.A2)
```

```
##
## Call:
## lm(formula = log(Erosion) ~ poly(Precipitation, 3), data = slopedata)
##
## Residuals:
##     Min      1Q  Median      3Q     Max
## -4.8787 -0.7841  0.0186  0.8131  4.2645
##
## Coefficients:
##                         Estimate Std. Error t value Pr(>|t|)
## (Intercept)              3.01717    0.04691  64.321  < 2e-16 ***
## poly(Precipitation, 3)1  3.35257    1.24462   2.694  0.00724 **
## poly(Precipitation, 3)2  0.25944    1.24462   0.208  0.83494
## poly(Precipitation, 3)3  9.38049    1.24462   7.537 1.49e-13 ***
## ---
## Signif. codes:  0 '***' 0.001 '**' 0.01 '*' 0.05 '.' 0.1 ' ' 1
##
## Residual standard error: 1.245 on 700 degrees of freedom
## Multiple R-squared:  0.08389,    Adjusted R-squared:  0.07997
## F-statistic: 21.37 on 3 and 700 DF,  p-value: 2.982e-13
```

```
ggplot(slopedata, aes(x=Precipitation, y=Erosion)) + geom_point() +
  stat_smooth(method="lm", formula=y ~ poly(x, 3, raw=TRUE)) + b + scale_y_log10()
```

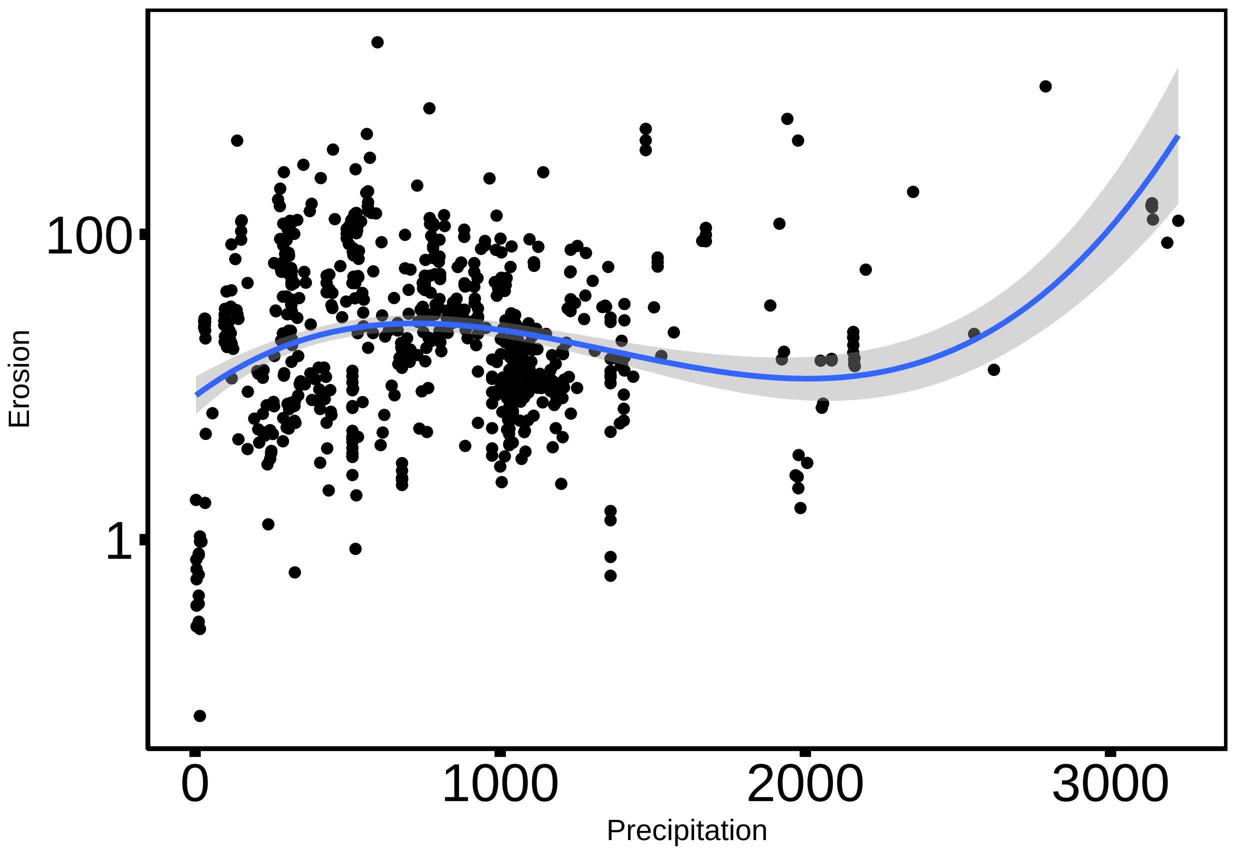


```
ggplot(slopedata, aes(x=Slope, y=Erosion)) + geom_point() +
  geom_smooth(method="lm") + b+ scale_y_log10()
```

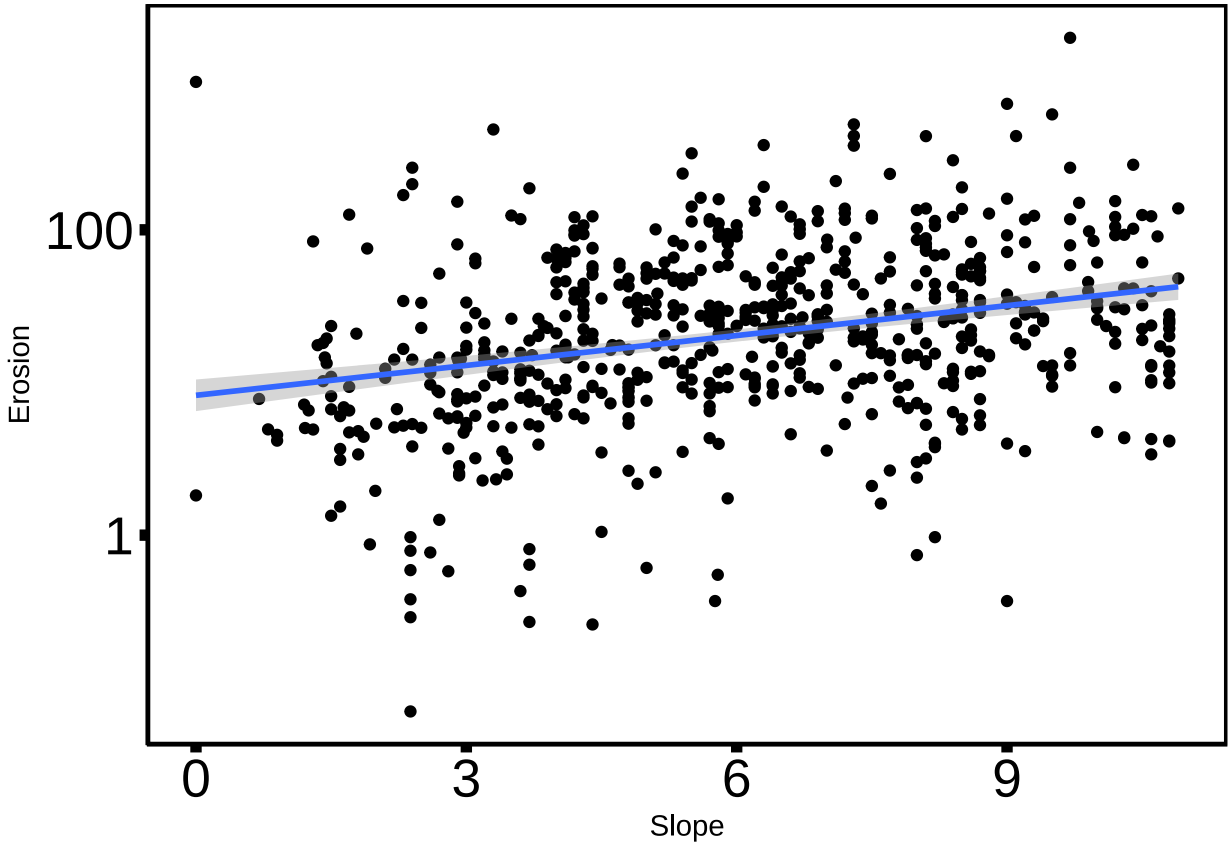

#Normalising the Data

```
normaldata<-read.csv(file.choose())
library(reshape2)
library(plotly)
```

```
##
## Attaching package: 'plotly'

## The following object is masked from 'package:ggplot2':
##
##     last_plot

## The following object is masked from 'package:stats':
##
##     filter

## The following object is masked from 'package:graphics':
##
##     layout
```

```
head(normaldata)
```

```
##   Study.number           Citation   Sample.ID
## 1           64 Placzek et al 2010    ADBA-5SD
## 2           64 Placzek et al 2010 ADBA-12SDsm
## 3           52 Nichols et al 2002       GMV-1
## 4           52 Nichols et al 2002       IMV-3
## 5           52 Nichols et al 2002       IMV-1
## 6           52 Nichols et al 2002       IMV-2
```

```
##                                       Location CronusID Latitude Longitude
## 1                        Atacama Desert, Chile      939    -23.4   -69.463
## 2                        Atacama Desert, Chile      940  -23.398   -69.462
## 3 Granite Mountain, Mojave Desert, CA, USA      759   34.026  -115.138
## 4   Iron Mountain, Mojavee Desert, CA, USA      762   34.109  -115.195
## 5   Iron Mountain, Mojavee Desert, CA, USA      764   34.117  -115.193
## 6   Iron Mountain, Mojavee Desert, CA, USA      760   34.116  -115.189
##   Elevation Slope Lithology Precipitation Temperature Vegetation Erosion
## 1      2040   3.7   Igneous            12          14          0    0.81
## 2      2043   3.6   Igneous            12          14          0    0.43
## 3       555   9.1   Igneous           117          21          0   33.56
## 4       695  22.6   Igneous           127          21          0   43.46
## 5       700  22.6   Igneous           131          20          0   44.58
## 6       703  23.4   Igneous           135          20          0   38.27
##    timescale  X
## 1  630417.65 NA
## 2 1161946.26 NA
## 3   17886.89 NA
## 4   13813.35 NA
## 5   13464.95 NA
## 6   15689.50 NA
```

PLotting Precipitation for diffrent lithology

```
normaldata.complete<-read.csv(file.choose())
p<- ggplot(normaldata.complete, aes(x=Precipitation, y=Erosion)) + geom_point() +
  stat_smooth(method="lm", formula=y ~ poly(x, 3, raw=TRUE)) + b + scale_y_log10()
p <- p + facet_wrap( ~Lithology, ncol=2)
```

```
p
```

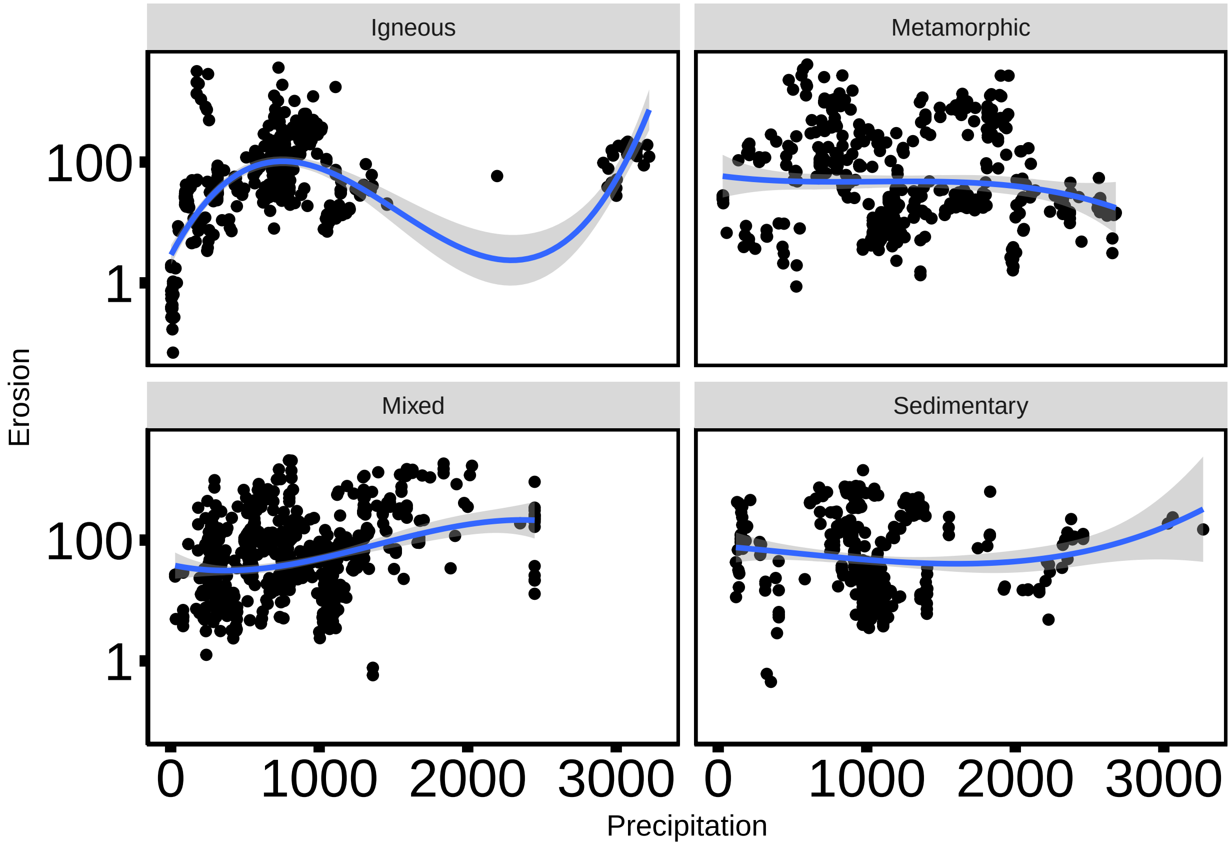

#Subsetting data based on Lithology

```
Lith1<-subset(normaldata,  Lithology=="Igneous")
Lith2<-subset(normaldata,  Lithology=="Metamorphic")
Lith3<-subset(normaldata,  Lithology=="Mixed")
Lith4<-subset(normaldata,  Lithology=="Sedimentary")
```

Statistic of erosion vs presicpitation in each Lithology

```
regression.Lith1<-lm(Erosion ~ poly(Precipitation, 3), data=Lith1)
regression.Lith2<-lm(Erosion ~ poly(Precipitation, 3), data=Lith2)
regression.Lith3<-lm(Erosion ~ poly(Precipitation, 3), data=Lith3)
regression.Lith4<-lm(Erosion ~ poly(Precipitation, 3), data=Lith4)
summary(regression.Lith1)
```

```
##
## Call:
## lm(formula = Erosion ~ poly(Precipitation, 3), data = Lith1)
##
## Residuals:
##    Min     1Q Median     3Q    Max
## -187.9 -138.3  -93.8  -40.4 3460.3
##
## Coefficients:
##                         Estimate Std. Error t value Pr(>|t|)
```

```
## (Intercept)               160.81      20.88   7.702 1.38e-13 ***
## poly(Precipitation, 3)1  -100.17     392.82  -0.255   0.7989
## poly(Precipitation, 3)2  -336.26     392.82  -0.856   0.3926
## poly(Precipitation, 3)3   667.45     392.82   1.699   0.0902 .
## ---
## Signif. codes:  0 '***' 0.001 '**' 0.01 '*' 0.05 '.' 0.1 ' ' 1
##
## Residual standard error: 392.8 on 350 degrees of freedom
## Multiple R-squared:  0.01042,    Adjusted R-squared:  0.001936
## F-statistic: 1.228 on 3 and 350 DF,  p-value: 0.2993
```

```r
summary(regression.Lith2)
```

```
##
## Call:
## lm(formula = Erosion ~ poly(Precipitation, 3), data = Lith2)
##
## Residuals:
##    Min     1Q Median     3Q    Max
## -271.5 -233.9 -176.0  -62.5 3846.7
##
## Coefficients:
##                         Estimate Std. Error t value Pr(>|t|)
## (Intercept)               229.92      24.51   9.381   <2e-16 ***
## poly(Precipitation, 3)1  -781.17     499.91  -1.563    0.119
## poly(Precipitation, 3)2  -327.06     499.91  -0.654    0.513
## poly(Precipitation, 3)3   152.21     499.91   0.304    0.761
## ---
## Signif. codes:  0 '***' 0.001 '**' 0.01 '*' 0.05 '.' 0.1 ' ' 1
##
## Residual standard error: 499.9 on 412 degrees of freedom
## Multiple R-squared:  0.007139,   Adjusted R-squared:  -9.032e-05
## F-statistic: 0.9875 on 3 and 412 DF,  p-value: 0.3985
```

```r
summary(regression.Lith3)
```

```
##
## Call:
## lm(formula = Erosion ~ poly(Precipitation, 3), data = Lith3)
##
## Residuals:
##     Min      1Q  Median      3Q     Max
## -402.54 -143.38  -58.24   16.74 1999.40
##
## Coefficients:
##                         Estimate Std. Error t value Pr(>|t|)
## (Intercept)               148.31      11.05  13.426  < 2e-16 ***
## poly(Precipitation, 3)1  2000.57     272.17   7.350 6.46e-13 ***
## poly(Precipitation, 3)2   344.89     272.17   1.267    0.206
## poly(Precipitation, 3)3 -1199.17     272.17  -4.406 1.25e-05 ***
## ---
## Signif. codes:  0 '***' 0.001 '**' 0.01 '*' 0.05 '.' 0.1 ' ' 1
##
## Residual standard error: 272.2 on 603 degrees of freedom
## Multiple R-squared:  0.1107, Adjusted R-squared:  0.1063
```

```
## F-statistic: 25.02 on 3 and 603 DF,  p-value: 2.893e-15
```

```r
summary(regression.Lith4)
```

```
##
## Call:
## lm(formula = Erosion ~ poly(Precipitation, 3), data = Lith4)
##
## Residuals:
##     Min      1Q  Median      3Q     Max
## -159.09 -125.38  -91.58   32.15 1288.40
##
## Coefficients:
##                         Estimate Std. Error t value Pr(>|t|)
## (Intercept)               137.09      10.56  12.976   <2e-16 ***
## poly(Precipitation, 3)1  -306.74     195.95  -1.565    0.118
## poly(Precipitation, 3)2    34.55     195.95   0.176    0.860
## poly(Precipitation, 3)3   299.88     195.95   1.530    0.127
## ---
## Signif. codes:  0 '***' 0.001 '**' 0.01 '*' 0.05 '.' 0.1 ' ' 1
##
## Residual standard error: 196 on 340 degrees of freedom
## Multiple R-squared:  0.01399,    Adjusted R-squared:  0.005288
## F-statistic: 1.608 on 3 and 340 DF,  p-value: 0.1873
```

now subsetting Lithology and Elevation (>1000M)

```r
subL1<-subset(normaldata, Elevation > 1000 & Lithology=="Igneous")
subL2<-subset(normaldata, Elevation > 1000 & Lithology=="Metamorphic")
subL3<-subset(normaldata, Elevation > 1000 & Lithology=="Mixed")
subL4<-subset(normaldata, Elevation > 1000 & Lithology=="Sedimentary")
```

statiscis opf erosion vs precipitation for all sites with >1000m elevation and each Lithology

```r
regression.subL1<-lm(Erosion ~ poly(Precipitation, 3), data=subL1)
regression.subL2<-lm(Erosion ~ poly(Precipitation, 3), data=subL2)
regression.subL3<-lm(Erosion ~ poly(Precipitation, 3), data=subL3)
regression.subL4<-lm(Erosion ~ poly(Precipitation, 3), data=subL4)
summary(regression.subL1)
```

```
##
## Call:
## lm(formula = Erosion ~ poly(Precipitation, 3), data = subL1)
##
## Residuals:
##    Min     1Q Median     3Q    Max
## -207.2 -163.1 -141.2  -63.1 3444.2
##
## Coefficients:
```

```
##                           Estimate Std. Error t value Pr(>|t|)
## (Intercept)                 198.67      28.05   7.083 1.35e-11 ***
## poly(Precipitation, 3)1   316.63     453.15   0.699    0.485
## poly(Precipitation, 3)2  -101.89     453.15  -0.225    0.822
## poly(Precipitation, 3)3    40.99     453.15   0.090    0.928
## ---
## Signif. codes:  0 '***' 0.001 '**' 0.01 '*' 0.05 '.' 0.1 ' ' 1
##
## Residual standard error: 453.1 on 257 degrees of freedom
## Multiple R-squared:  0.002124,   Adjusted R-squared:  -0.009525
## F-statistic: 0.1823 on 3 and 257 DF,  p-value: 0.9084
```

```r
summary(regression.subL2)
```

```
##
## Call:
## lm(formula = Erosion ~ poly(Precipitation, 3), data = subL2)
##
## Residuals:
##    Min     1Q Median     3Q    Max
## -453.5 -305.8 -178.8    9.7 3674.7
##
## Coefficients:
##                           Estimate Std. Error t value Pr(>|t|)
## (Intercept)                 334.85      34.09   9.823  < 2e-16 ***
## poly(Precipitation, 3)1 -1605.31     570.41  -2.814  0.00524 **
## poly(Precipitation, 3)2 -1199.17     570.41  -2.102  0.03643 *
## poly(Precipitation, 3)3   658.18     570.41   1.154  0.24955
## ---
## Signif. codes:  0 '***' 0.001 '**' 0.01 '*' 0.05 '.' 0.1 ' ' 1
##
## Residual standard error: 570.4 on 276 degrees of freedom
## Multiple R-squared:  0.0472, Adjusted R-squared:  0.03684
## F-statistic: 4.557 on 3 and 276 DF,  p-value: 0.003906
```

```r
summary(regression.subL3)
```

```
##
## Call:
## lm(formula = Erosion ~ poly(Precipitation, 3), data = subL3)
##
## Residuals:
##     Min      1Q  Median      3Q     Max
## -619.91 -114.18  -55.26   42.33 1139.57
##
## Coefficients:
##                           Estimate Std. Error t value Pr(>|t|)
## (Intercept)                 201.06      13.66  14.720  < 2e-16 ***
## poly(Precipitation, 3)1  2932.68     252.61  11.610  < 2e-16 ***
## poly(Precipitation, 3)2   776.41     252.61   3.074  0.00229 **
## poly(Precipitation, 3)3 -1006.06     252.61  -3.983 8.35e-05 ***
## ---
## Signif. codes:  0 '***' 0.001 '**' 0.01 '*' 0.05 '.' 0.1 ' ' 1
##
## Residual standard error: 252.6 on 338 degrees of freedom
```

```
## Multiple R-squared:  0.3214, Adjusted R-squared:  0.3154
## F-statistic: 53.36 on 3 and 338 DF,  p-value: < 2.2e-16
```

```r
summary(regression.subL4)
```

```
##
## Call:
## lm(formula = Erosion ~ poly(Precipitation, 3), data = subL4)
##
## Residuals:
##     Min      1Q  Median      3Q     Max
## -362.41 -135.82  -45.48  155.06  409.22
##
## Coefficients:
##                         Estimate Std. Error t value Pr(>|t|)
## (Intercept)               303.59      19.59  15.494  < 2e-16 ***
## poly(Precipitation, 3)1   316.75     199.82   1.585    0.116
## poly(Precipitation, 3)2  -856.00     199.82  -4.284 4.23e-05 ***
## poly(Precipitation, 3)3  -145.40     199.82  -0.728    0.469
## ---
## Signif. codes:  0 '***' 0.001 '**' 0.01 '*' 0.05 '.' 0.1 ' ' 1
##
## Residual standard error: 199.8 on 100 degrees of freedom
## Multiple R-squared:  0.1762, Adjusted R-squared:  0.1515
## F-statistic: 7.131 on 3 and 100 DF,  p-value: 0.0002173
```

now subsetting Lithology and Elevation (<1000M)

```r
subL1<-subset(normaldata, Elevation < 1000 & Lithology=="Igneous")
subL2<-subset(normaldata, Elevation < 1000 & Lithology=="Metamorphic")
subL3<-subset(normaldata, Elevation < 1000 & Lithology=="Mixed")
subL4<-subset(normaldata, Elevation < 1000 & Lithology=="Sedimentary")
```

statiscis of erosion vs precipitation for all sites with >1000m elevation and each Lithology

```r
regression.subL1<-lm(Erosion ~ poly(Precipitation, 3), data=subL1)
regression.subL2<-lm(Erosion ~ poly(Precipitation, 3), data=subL2)
regression.subL3<-lm(Erosion ~ poly(Precipitation, 3), data=subL3)
regression.subL4<-lm(Erosion ~ poly(Precipitation, 3), data=subL4)
summary(regression.subL1)
```

```
##
## Call:
## lm(formula = Erosion ~ poly(Precipitation, 3), data = subL1)
##
## Residuals:
##     Min      1Q  Median      3Q     Max
## -83.721 -13.670  -0.384  13.010  89.091
##
```

```
## Coefficients:
##                         Estimate Std. Error t value Pr(>|t|)
## (Intercept)               54.574      3.216  16.969  < 2e-16 ***
## poly(Precipitation, 3)1  378.232     31.015  12.195  < 2e-16 ***
## poly(Precipitation, 3)2  171.113     31.015   5.517 3.35e-07 ***
## poly(Precipitation, 3)3   93.036     31.015   3.000   0.0035 **
## ---
## Signif. codes:  0 '***' 0.001 '**' 0.01 '*' 0.05 '.' 0.1 ' ' 1
##
## Residual standard error: 31.01 on 89 degrees of freedom
## Multiple R-squared:  0.6789, Adjusted R-squared:  0.6681
## F-statistic: 62.72 on 3 and 89 DF,  p-value: < 2.2e-16
```

```r
summary(regression.subL2)
```

```
##
## Call:
## lm(formula = Erosion ~ poly(Precipitation, 3), data = subL2)
##
## Residuals:
##     Min      1Q  Median      3Q     Max
## -14.461  -4.598  -2.394   0.968 113.896
##
## Coefficients:
##                         Estimate Std. Error t value Pr(>|t|)
## (Intercept)               13.878      1.036  13.393   <2e-16 ***
## poly(Precipitation, 3)1  -22.738     12.085  -1.882   0.0621 .
## poly(Precipitation, 3)2   26.921     12.085   2.228   0.0276 *
## poly(Precipitation, 3)3  -29.149     12.085  -2.412   0.0172 *
## ---
## Signif. codes:  0 '***' 0.001 '**' 0.01 '*' 0.05 '.' 0.1 ' ' 1
##
## Residual standard error: 12.08 on 132 degrees of freedom
## Multiple R-squared:  0.09787,    Adjusted R-squared:  0.07737
## F-statistic: 4.774 on 3 and 132 DF,  p-value: 0.003427
```

```r
summary(regression.subL3)
```

```
##
## Call:
## lm(formula = Erosion ~ poly(Precipitation, 3), data = subL3)
##
## Residuals:
##     Min      1Q  Median      3Q     Max
## -215.03  -65.08  -54.97  -24.29 2004.60
##
## Coefficients:
##                         Estimate Std. Error t value Pr(>|t|)
## (Intercept)                80.23      15.18   5.287 2.63e-07 ***
## poly(Precipitation, 3)1   386.63     247.04   1.565    0.119
## poly(Precipitation, 3)2   253.20     247.04   1.025    0.306
## poly(Precipitation, 3)3   350.30     247.04   1.418    0.157
## ---
## Signif. codes:  0 '***' 0.001 '**' 0.01 '*' 0.05 '.' 0.1 ' ' 1
##
```

```
## Residual standard error: 247 on 261 degrees of freedom
## Multiple R-squared:  0.02068,    Adjusted R-squared:  0.009421
## F-statistic: 1.837 on 3 and 261 DF,  p-value: 0.1408
```

```
summary(regression.subL4)
```

```
##
## Call:
## lm(formula = Erosion ~ poly(Precipitation, 3), data = subL4)
##
## Residuals:
##     Min      1Q  Median      3Q     Max
## -101.00  -49.75  -41.44   -3.12 1371.04
##
## Coefficients:
##                         Estimate Std. Error t value Pr(>|t|)
## (Intercept)               64.949      8.596   7.556 9.14e-13 ***
## poly(Precipitation, 3)1  161.273    133.170   1.211    0.227
## poly(Precipitation, 3)2  106.943    133.170   0.803    0.423
## poly(Precipitation, 3)3  195.801    133.170   1.470    0.143
## ---
## Signif. codes:  0 '***' 0.001 '**' 0.01 '*' 0.05 '.' 0.1 ' ' 1
##
## Residual standard error: 133.2 on 236 degrees of freedom
## Multiple R-squared:  0.01779,    Adjusted R-squared:  0.005299
## F-statistic: 1.424 on 3 and 236 DF,  p-value: 0.2363
```

Now For sites with same slope and Elevation

```
sub2<-subset(normaldata, Slope <11 & Elevation < 1000)
sub3<-subset(normaldata, Slope >11 & Elevation < 1000)
sub4<-subset(normaldata, Slope <11 & Elevation > 1000)
sub5<-subset(normaldata, Slope >11 & Elevation > 1000)
head (sub3)
```

```
##    Study.number                 Citation Sample.ID
## 4            52       Nichols et al 2002     IMV-3
## 5            52       Nichols et al 2002     IMV-1
## 6            52       Nichols et al 2002     IMV-2
## 7            52       Nichols et al 2002     GMV-3
## 66           13 Carretier, et al. (2013) HUA7
## 67           13 Carretier, et al. (2013) HUA1
##                                    Location CronusID Latitude Longitude
## 4    Iron Mountain, Mojavee Desert, CA, USA      762   34.109  -115.195
## 5    Iron Mountain, Mojavee Desert, CA, USA      764   34.117  -115.193
## 6    Iron Mountain, Mojavee Desert, CA, USA      760   34.116  -115.189
## 7  Granite Mountain, Mojave Desert, CA, USA      761   34.012  -115.134
## 66                                Huasco        1627    -28.8    -70.46
## 67                                Huasco        1628   -28.99    -70.28
##    Elevation    Slope Lithology Precipitation Temperature Vegetation
## 4        695 22.60000   Igneous     127.00000          21       0.00
## 5        700 22.60000   Igneous     131.00000          20       0.00
## 6        703 23.40000   Igneous     135.00000          20       0.00
```

```
## 7        653 15.20000   Igneous     136.00000          21       0.00
## 66       600 24.22775     Mixed      83.96829          NA       3.50
## 67       600 24.22775     Mixed      83.96829          NA       3.57
##    Erosion timescale  X
## 4    43.46  13813.35 NA
## 5    44.58  13464.95 NA
## 6    38.27  15689.50 NA
## 7    40.79  14715.49 NA
## 66    3.76 126893.49 NA
## 67    6.95  67187.37 NA
```

```
regression.sub2<-lm(Erosion ~ poly(Precipitation, 3), data=sub2)
regression.sub3<-lm(Erosion ~ poly(Precipitation, 3), data=sub3)
regression.sub4<-lm(Erosion ~ poly(Precipitation, 3), data=sub4)
regression.sub5<-lm(Erosion ~ poly(Precipitation, 3), data=sub5)
summary(regression.sub2)
```

```
##
## Call:
## lm(formula = Erosion ~ poly(Precipitation, 3), data = sub2)
##
## Residuals:
##     Min      1Q  Median      3Q     Max
## -117.59  -16.53   -8.38    3.90  789.86
##
## Coefficients:
##                         Estimate Std. Error t value Pr(>|t|)
## (Intercept)               30.797      2.597  11.857  < 2e-16 ***
## poly(Precipitation, 3)1  329.128     51.687   6.368 5.38e-10 ***
## poly(Precipitation, 3)2  339.051     51.687   6.560 1.71e-10 ***
## poly(Precipitation, 3)3   58.967     51.687   1.141    0.255
## ---
## Signif. codes:  0 '***' 0.001 '**' 0.01 '*' 0.05 '.' 0.1 ' ' 1
##
## Residual standard error: 51.69 on 392 degrees of freedom
## Multiple R-squared:  0.178,  Adjusted R-squared:  0.1717
## F-statistic: 28.29 on 3 and 392 DF,  p-value: < 2.2e-16
```

```
summary(regression.sub3)
```

```
##
## Call:
## lm(formula = Erosion ~ poly(Precipitation, 3), data = sub3)
##
## Residuals:
##     Min      1Q  Median      3Q     Max
## -223.18  -80.59  -70.37  -41.49 2297.19
##
## Coefficients:
##                         Estimate Std. Error t value Pr(>|t|)
## (Intercept)               111.39      13.98   7.970 2.02e-14 ***
## poly(Precipitation, 3)1   818.08     269.58   3.035  0.00258 **
## poly(Precipitation, 3)2   440.65     269.58   1.635  0.10299
## poly(Precipitation, 3)3   135.64     269.58   0.503  0.61516
## ---
```

```
## Signif. codes:  0 '***' 0.001 '**' 0.01 '*' 0.05 '.' 0.1 ' ' 1
##
## Residual standard error: 269.6 on 368 degrees of freedom
## Multiple R-squared:  0.03192,    Adjusted R-squared:  0.02403
## F-statistic: 4.045 on 3 and 368 DF,  p-value: 0.007541
```

```r
summary(regression.sub4)
```

```
##
## Call:
## lm(formula = Erosion ~ poly(Precipitation, 3), data = sub4)
##
## Residuals:
##     Min      1Q  Median      3Q     Max
##  -95.20  -53.19  -29.68    6.59 1733.17
##
## Coefficients:
##                         Estimate Std. Error t value Pr(>|t|)
## (Intercept)                64.59       7.52   8.590 4.63e-16 ***
## poly(Precipitation, 3)1   224.40     131.97   1.700   0.0901 .
## poly(Precipitation, 3)2  -281.08     131.97  -2.130   0.0340 *
## poly(Precipitation, 3)3    21.88     131.97   0.166   0.8685
## ---
## Signif. codes:  0 '***' 0.001 '**' 0.01 '*' 0.05 '.' 0.1 ' ' 1
##
## Residual standard error: 132 on 304 degrees of freedom
## Multiple R-squared:  0.02394,    Adjusted R-squared:  0.01431
## F-statistic: 2.485 on 3 and 304 DF,  p-value: 0.06079
```

```r
summary(regression.sub5)
```

```
##
## Call:
## lm(formula = Erosion ~ poly(Precipitation, 3), data = sub5)
##
## Residuals:
##    Min     1Q Median     3Q    Max
## -368.6 -258.6 -172.0   66.4 3825.1
##
## Coefficients:
##                         Estimate Std. Error t value Pr(>|t|)
## (Intercept)               320.22      18.33  17.472   <2e-16 ***
## poly(Precipitation, 3)1   182.06     487.66   0.373   0.7090
## poly(Precipitation, 3)2 -1228.28     487.66  -2.519   0.0120 *
## poly(Precipitation, 3)3  -827.14     487.66  -1.696   0.0903 .
## ---
## Signif. codes:  0 '***' 0.001 '**' 0.01 '*' 0.05 '.' 0.1 ' ' 1
##
## Residual standard error: 487.7 on 704 degrees of freedom
## Multiple R-squared:  0.01312,    Adjusted R-squared:  0.008916
## F-statistic:  3.12 on 3 and 704 DF,  p-value: 0.0255
```

```r
sub6<-subset(normaldata, Vegetation <40)
sub7<-subset(normaldata, Vegetation >40)
sub8<-subset(normaldata, Vegetation <40 & Elevation < 1000)
sub9<-subset(normaldata, Vegetation >40 & Elevation < 1000)
```

```r
regression.sub6<-lm(Erosion ~ poly(Precipitation, 3), data=sub6)
regression.sub7<-lm(Erosion ~ poly(Precipitation, 3), data=sub7)
regression.sub8<-lm(Erosion ~ poly(Precipitation, 3), data=sub8)
regression.sub9<-lm(Erosion ~ poly(Precipitation, 3), data=sub9)
summary(regression.sub6)
```

```
##
## Call:
## lm(formula = Erosion ~ poly(Precipitation, 3), data = sub6)
##
## Residuals:
##    Min     1Q Median     3Q    Max
## -496.3 -153.3 -102.8  -24.9 3936.1
##
## Coefficients:
##                         Estimate Std. Error t value Pr(>|t|)
## (Intercept)               225.62      16.18  13.949  < 2e-16 ***
## poly(Precipitation, 3)1  3401.66     445.93   7.628 7.16e-14 ***
## poly(Precipitation, 3)2  -242.49     445.93  -0.544  0.58675
## poly(Precipitation, 3)3 -1216.74     445.93  -2.729  0.00651 **
## ---
## Signif. codes:  0 '***' 0.001 '**' 0.01 '*' 0.05 '.' 0.1 ' ' 1
##
## Residual standard error: 445.9 on 756 degrees of freedom
## Multiple R-squared:  0.08022,    Adjusted R-squared:  0.07657
## F-statistic: 21.98 on 3 and 756 DF,  p-value: 1.184e-13
```

```r
summary(regression.sub7)
```

```
##
## Call:
## lm(formula = Erosion ~ poly(Precipitation, 3), data = sub7)
##
## Residuals:
##     Min      1Q  Median      3Q     Max
## -159.42 -117.45  -78.67    8.70 2565.31
##
## Coefficients:
##                         Estimate Std. Error t value Pr(>|t|)
## (Intercept)              127.836      8.347  15.315   <2e-16 ***
## poly(Precipitation, 3)1 -557.989    245.496  -2.273   0.0233 *
## poly(Precipitation, 3)2  362.276    245.496   1.476   0.1404
## poly(Precipitation, 3)3  250.950    245.496   1.022   0.3070
## ---
## Signif. codes:  0 '***' 0.001 '**' 0.01 '*' 0.05 '.' 0.1 ' ' 1
##
## Residual standard error: 245.5 on 861 degrees of freedom
## Multiple R-squared:  0.009649,   Adjusted R-squared:  0.006198
## F-statistic: 2.796 on 3 and 861 DF,  p-value: 0.03925
```

```r
summary(regression.sub8)
```

```
##
## Call:
## lm(formula = Erosion ~ poly(Precipitation, 3), data = sub8)
```

```
##
## Residuals:
##     Min      1Q  Median      3Q     Max
##  -92.25  -70.58  -36.06    8.23 1991.15
##
## Coefficients:
##                         Estimate Std. Error t value Pr(>|t|)
## (Intercept)                63.71      16.95   3.759 0.000226 ***
## poly(Precipitation, 3)1   248.67     238.50   1.043 0.298404
## poly(Precipitation, 3)2  -371.12     238.50  -1.556 0.121319
## poly(Precipitation, 3)3   243.83     238.50   1.022 0.307881
## ---
## Signif. codes:  0 '***' 0.001 '**' 0.01 '*' 0.05 '.' 0.1 ' ' 1
##
## Residual standard error: 238.5 on 194 degrees of freedom
## Multiple R-squared:  0.02293,    Adjusted R-squared:  0.007825
## F-statistic: 1.518 on 3 and 194 DF,  p-value: 0.2112
```

```r
summary(regression.sub9)
```

```
##
## Call:
## lm(formula = Erosion ~ poly(Precipitation, 3), data = sub9)
##
## Residuals:
##     Min      1Q  Median      3Q     Max
## -179.83  -40.68  -31.10   -8.67 1380.84
##
## Coefficients:
##                         Estimate Std. Error t value Pr(>|t|)
## (Intercept)               61.205      6.452   9.486  < 2e-16 ***
## poly(Precipitation, 3)1  360.065    137.329   2.622  0.00904 **
## poly(Precipitation, 3)2  393.940    137.329   2.869  0.00432 **
## poly(Precipitation, 3)3 -319.255    137.329  -2.325  0.02053 *
## ---
## Signif. codes:  0 '***' 0.001 '**' 0.01 '*' 0.05 '.' 0.1 ' ' 1
##
## Residual standard error: 137.3 on 449 degrees of freedom
## Multiple R-squared:  0.04368,    Adjusted R-squared:  0.03729
## F-statistic: 6.836 on 3 and 449 DF,  p-value: 0.0001637
```
