# Supplementary material for: Coupled influence of precipitation and vegetation on millennial-scale erosion rates derived from 10Be
Source: PLoS One. 2019 Jan 25;14(1):e0211325. doi: 10.1371/journal.pone.0211325 (PMC6347257; doi:10.1371/journal.pone.0211325)
Supplement: S3 File — (PDF) [file pone.0211325.s005.pdf]

Supplementary material for: Coupled influence of precipitation and vegetation on millennial-scale erosion rates derived from ^{10}Be

Ashish Kumar Mishra[1*], Christa Placzek[1], and Rhondda Jones[2]

[1]Geosciences, College of Science and Engineering and Centre for Tropical Environmental and Sustainability Science (TESS), James Cook University, Townsville, Queensland, Australia, 4810

[2]Emeritus Professor, Coordinator, StatsHelp Service, Graduate Research School, James Cook University, Townsville, Queensland, Australia, 4810

DATA COMPILATION:

Cosmogenic ^{10}Be-derived basin average erosion rates were compiled from various published studies conducted all over the world. Most of the studies of this compilation were included in Portenga & Bierman (2011), and Willenbring (2013); however, we have added more studies conducted after these publications. We also recalculated erosion rate for all studies, based on the latest update of CRONUS (Version 2.3) (Balco, Stone, Lifton, & Dunai, 2008)(hess.ess.washington.edu). The information required for CRONUS erosion rate calculations was retrieved from published studies. Most studies did not have any information on sample thickness and crustal density. Therefore, sample thickness was assumed 1 cm and crustal density as 2.7g/cm^3. The geographic coordinates of the samples in the dataset are expressed in Decimal Degrees and the spatial reference system is WGS84.

REFERENCES FOR DATA:

1. Abbühl, L. M., Norton, K. P., Schlunegger, F., Kracht, O., Aldahan, A., and Possnert, G., 2010, El Niño forcing on ^{10}Be-based surface denudation rates in the north-western Peruvian Andes: Geomorphology, v. 123, p. 257-268.
2. Acosta, V. T., Schildgen, T. F., Clarke, B. A., Scherler, D., Bookhagen, B., Wittmann, H., Strecker, M. R. (2015). Effect of vegetation cover on millennial-scale landscape denudation rates in East Africa. Lithosphere, L402. 401.
3. Balco, G., Stone, J. O., Lifton, N. A., & Dunai, T. J. (2008). A complete and easily accessible means of calculating surface exposure ages or erosion rates from 10 Be and 26 Al measurements. Quaternary Geochronology, 3(3), 174-195.

4. Belmont, P., Pazzaglia, F. J., and Gosse, J. C., 2007, Cosmogenic ^{10}Be as a tracer for hillslope and channel sediment dynamics in the Clearwater River, western Washington State: Earth and Planetary Science Letters.
5. Bierman, P. R., Albrecht, A., Bothner, M., Brown, E., Bullen, T., Gray, L., and Turpin, L., 1998, Weathering, erosion and sedimentation, *in* Kendall, C., and McDonnell, J. J., eds., Isotope Tracers in Catchment Hydrology, Elsevier, p. 647-678.
6. Bierman, P. R., and Caffee, M. W., 2001, Slow rates of rock surface erosion and sediment production across the Namib Desert and escarpment, Southern Africa: American Journal of Science, v. 301, no. 4-5, p. 326-358.
7. Bierman, P. R., and Steig, E., 1996, Estimating rates of denudation and sediment transport using cosmogenic isotope abundances in sediment: Earth Surface Processes and Landforms, v. 21, p. 125-139.
8. Bierman, P. R., Clapp, E. M., Nichols, K. K., Gillespie, A. R., and Caffee, M., 2001, Using cosmogenic nuclide measurements in sediments to understand background rates of erosion and sediment transport,, *in* Harmon, R. S., and Doe, W. M., eds., Landscape Erosion and Evolution Modelling: New York, Kluwer, p. 89-116.
9. Bierman, P. R., Nichols, K. K., Matmon, A., Enzel, Y., Larsen, J., and Finkel, R., 2007, 10-Be shows that Namibian drainage basins are slowly, steadily and uniformly eroding: Quaternary International v. 167–168 no. 3, p. 33.
10. Bierman, P. R., Reusser, L. J., Nichols, K. K., Matmon, A., and Rood, D., 2009, Where is the sediment coming from and where is it going - A 10Be examination of the northern Queensland escarpment, Australia, 2009 Portland GSA Annual Meeting: Portland, Or.
11. Bierman, P. R., Reuter, J. M., Pavich, M., Gellis, A. C., Caffee, M. W., and Larsen, J., 2005, Using cosmogenic nuclides to contrast rates of erosion and sediment yield in a semi-arid, arroyo-dominated landscape, Rio Puerco Basin, New Mexico: Earth Surface Processes and Landforms, v. 30, no. 8, p. 935-953.
12. Binnie, S. A., Phillips, W. M., Summerfield, M. A., and Fifield, L. K., 2006, Sediment mixing and basin-wide cosmogenic nuclide analysis in rapidly eroding mountainous environments: Quaternary Geochronology v. 1, p. 4-14.
13. Binnie, S. A., Phillips, W. M., Summerfield, M. A., Fifield, L. K., and Spotila, J. A., 2008, Patterns of denudation through time in the San Bernardino Mountains, California: Implications for early-stage orogenesis Earth and Planetary Science Letters, v. In Press.
14. Brown, E. T., Stallard, R. F., Larsen, M. C., Bourles, D. L., Raisbeck, G. M., and Yiou, F., 1998, Determination of predevelopment denudation rates of an agricultural watershed (Cayaguas River, Puerto Rico) using in-situ-produced ^{10}Be in river-borne quartz: Earth and Planetary Science Letters, v. 160, no. 3-4, p. 723-728.
15. Buechi, M., Kober, F., Ivy-Ochs, S., Salcher, B., Kubik, P., & Christl, M. 2014. Denudation rates of small transient catchments controlled by former glaciation: The Hörnli nunatak in the northeastern Swiss Alpine Foreland. Quaternary Geochronology, 19, 135-147.

16. Carretier, S., Regard, V., Vassallo, R., Aguilar, G., Martinod, J., Riquelme, R., Farías, M. (2013). Slope and climate variability control of erosion in the Andes of central Chile. Geology, 41(2), 195-198.
17. Chappell, J., Zheng, H., and Fifield, K., 2006, Yangtse River sediments and erosion rates from source to sink traced with cosmogenic ^{10}Be: Sediments from major rivers: Palaeogeography, Palaeoclimatology, Palaeoecology, v. 241, no. 1, p. 79-94.
18. Clapp, E. M., Bierman, P. R., Nichols, K. K., Pavich, M., and Caffee, M., 2001, Rates of sediment supply to arroyos from upland erosion determined using in situ produced cosmogenic ^{10}Be and ^{26}Al: Quaternary Research (New York), v. 55, no. 2, p. 235-245.
19. Clapp, E. M., Bierman, P. R., Schick, A. P., Lekach, J., Enzel, Y., and Caffee, M., 2000, Sediment yield exceeds sediment production in arid region drainage basins: Geology, v. 28, no. 11, p. 995-998.
20. Clapp, E., Bierman, P. R., and Caffee, M., 2002, Using ^{10}Be and ^{26}Al to determine sediment generation rates and identify sediment source areas in an arid region drainage basin: Geomorphology, v. 45, no. 1,2, p. 89-104.
21. Codilean, A. T., Bishop, P., Stuart, F. M., Hoey, T. B., Fabel, D., & Freeman, S. P. 2008. Single-grain cosmogenic 21Ne concentrations in fluvial sediments reveal spatially variable erosion rates. Geology, 36(2), 159-162.
22. Codilean, A. T., Fenton, C. R., Fabel, D., Bishop, P., & Xu, S. (2014). Discordance between cosmogenic nuclide concentrations in amalgamated sands and individual fluvial pebbles in an arid zone catchment. Quaternary Geochronology, 19, 173-180.
23. Cox, R., Bierman, P. R., Jungers, M., Rakotondrazafy, A. F. M., and Finkel, R. C., 2006, Just how fast does Madagascar erode? Evidence from ^{10}Be analysis of lavaka, slope, and river sediment: Geological Society of America *Abstracts with Programs*, v. 38, no. 7, p. 278.
24. Croke, J., Bartley, R., Chappell, J., Austin, J. M., Fifield, K., Tims, S. G., Furuichi, T. (2015). 10Be-derived denudation rates from the Burdekin catchment: the largest contributor of sediment to the Great Barrier Reef. Geomorphology.
25. Cyr, A. J., and Granger, D. E., 2008, Dynamic equilibrium among erosion, river incision, and coastal uplift in the northern and central Apennines, Italy: Geology, v. 36, no. 2, p. 103- 106.
26. Cyr, A. J., Granger, D. E., Olivetti, V., and Molin, P., 2010, Quantifying rock uplift rates using channel steepness and cosmogenic nuclide-determined erosion rates: examples from northern and southern Italy: Lithosphere, v. 2, no. 3, p. 188-198.
27. Delunel, R., Beek, P. A. v. d., Carcaillet, J., Bourlès, D. L., and Valla, P. G., 2010, Frost- cracking control on catchment denudation rates: Insights from in situ produced ^{10}Be concentrations in stream sediments (Ecrins–Pelvoux massif, French Western Alps): Earth and Planetary Science Letters, v. 293, p. 72-83.
28. Delunel, R., van der Beek, P. A., Carcaillet, J., Bourlès, D. L., & Valla, P. G. (2010). Frost-cracking control on catchment denudation rates: Insights from in situ produced 10Be concentrations in stream sediments (Ecrins–Pelvoux massif, French Western

Alps). Earth and Planetary Science Letters, 293(1–2), 72-83. doi:http://dx.doi.org/10.1016/j.epsl.2010.02.020

29. Densmore, A. L., Hetzel, R., Ivy-Ochs, S., Krugh, W. C., Dawers, N., & Kubik, P. (2009). Spatial variations in catchment-averaged denudation rates from normal fault footwalls. Geology, 37(12), 1139-1142. doi:10.1130/g30164a.1
30. DiBiase, R. A., Whipple, K. X., Heimsath, A. M., and Ouimet, W. B., 2009, Landscape form and millennial erosion rates in the San Gabriel Mountains, CA: Earth and Planetary Science Letters, v. 289, p. 134-144.
31. Dirks, P. H. G. M., Placzek, C. J., Fink, D., Dosseto, A., & Roberts, E. (2016). Using 10Be cosmogenic isotopes to estimate erosion rates and landscape changes during the Plio-Pleistocene in the Cradle of Humankind, South Africa. *Journal of Human Evolution, 96*, 19-34. doi:http://dx.doi.org/10.1016/j.jhevol.2016.03.002
32. Duxbury, J., 2009, Erosion rates in and around Shenandoah National Park, VA, determined using analysis of cosmogenic 10Be: University of Vermont, 134 p.
33. Duxbury, J., Bierman, P. R., Portenga, E. W., Pavich, M. J., Southworth, S., & Freeman, S. P. (2015). Erosion rates in and around Shenandoah National Park, Virginia, determined using analysis of cosmogenic 10Be. American Journal of Science, 315(1), 46-76.
34. Ferrier, K. L., Kirchner, J. W., and Finkel, R. C., 2005, Erosion rates over millennial and decadal timescales at Caspar Creek and Redwood Creek, Northern California Coast Ranges: Earth Surface Processes and Landforms, v. 30, no. 8, p. 1025-1038.
35. Finnegan, N. J., Hallet, B., Montgomery, D. R., Zeitler, P., Stone, J., Anders, A. M., and Yuping, L., 2008, coupling of rock uplift and river incision in the Namche Barwa-Gyala peri massif, Tibet: 2007 GSA Denver Annual Meeting.
36. Glotzbach, C., Röttjer, M., Hampel, A., Hetzel, R., & Kubik, P. W. (2014). Quantifying the impact of former glaciation on catchment-wide denudation rates derived from cosmogenic 10Be. *Terra Nova, 26*(3), 186-194. doi:10.1111/ter.12085
37. Godard, V., Lavé, J., Carcaillet, J., Cattin, R., Bourlès, D., and Zhu, J., 2010, Spatial distribution of denudation in Eastern Tibet and regressive erosion of plateau margins: Tectonophysics, v. 491, p.253-274.
38. Granger, D. E., Kirchner, J. W., and Finkel, R., 1996, Spatially averaged long-term erosion rates measured from in situ-produced cosmogenic nuclides in alluvial sediments: Journal of Geology, v. 104, no. 3, p. 249-257.
39. Guralnik, B., Matmon, A., Avni, Y., and Fink, D., 2010, ^{10}Be exposure ages of ancient desert pavements reveal Quaternary evolution of the Dead Sea drainage basin and rift margin tilting: Earth and Planetary Science Letters, v. 290, p. 132-141.
40. Harkins, N., Kirby, E., Heimsath, A., Robinson, R., and Reiser, U., 2007, Transient fluvial incision in the headwaters of the Yellow River, northeastern Tibet, China: Journal of Geophysical Research, v. 112., no. F03S04, p. 21.
41. Heimsath, A. M., Chappell, J., Fifield, K., 1999, Cosmogenic nuclides, topography, and the spatial variation of soil depth: Geomorphology, v. 27, no. 1-2, p. 151-172.

42. Heimsath, A. M., Chappell, J., Fifield, K., 2001a, Late Quaternary erosion in southeastern Australia: a field example using cosmogenic nuclides: Quaternary International, v. 83-85, p. 169-185.
43. Heimsath, A. M., Chappell, J., Fifield, K., 2001b, Stochastic processes of soil production and transport; erosion rates, topographic variation and cosmogenic nuclides in the Oregon Coast Range: Earth Surface Processes and Landforms, v. 26, no. 5, p. 531-552.
44. Heimsath, A. M., Chappell, J., Fifield, K., 2010, Eroding Australia: rates and processes from Bega Valley to Arnhem Land: Geological Society, London, Special Publications, v. 346, p. 225-241.
45. Heimsath, A. M., Fink, D., and Hancock, G. R., 2009, The 'humped' soil production function: eroding Arnhem Land, Australia: Earth Surface Processes and Landforms, vol. 34, p. 1674-1684.
46. Heimsath, A., Chappel, J., Finkel, R. C., Fifield, K., and Alimanovic, A., 2006, Escarpment Erosion and Landscape Evolution in Southeastern Australia: Special Papers-Geological Society of America, v. 398, p. 173.
47. Henck, A. C., Huntington, K. W., Stone, J. O., Montgomery, D. R., and Hallet, B., 2011, Spatial controls on erosion in the Three Rivers Region, southeastern Tibet and southwestern China: Earth and Planetary Science Letters, v. 303, p. 71-83.
48. Hewawasam, T., von Blackenburg, F., Schaller, M., and Kubik, P., 2003, Increase of human over natural erosion rates in tropical highlands constrained by cosmogenic nuclides: Geology, v. 31, no. 7, p. 597-600.
49. Hippe, K., Kober, F., Zeilinger, G., Ivy-Ochs, S., Maden, C., Wacker, L., Wieler, R. (2012). Quantifying denudation rates and sediment storage on the eastern Altiplano, Bolivia, using cosmogenic 10 Be, 26 Al, and in situ 14 C. Geomorphology, 179, 58-70.
50. Insel, N., Ehlers, T. A., Schaller, M., Barnes, J. B., Tawackoli, S., and Poulsen, C. J., 2010, Spatial and temporal variability in denudation across the Bolivian Andes from multiple geochronometers: 2010, v. 122, p. 65-77.
51. Kirchner, J. W., Finkel, R. C., Riebe, C. S., Granger, D. E., Clayton, J. L., King, J. G., and Megahan, W. F., 2001, Mountain erosion over 10 yr, 10 k.y., and 10 m.y. time scales: Geology, v. 29, no. 7, p. 591-594.
52. Kober, F., Ivy-Ochs, S., Zeilinger, G., Schlunegger, F., Kubik, P. W., Baur, H., and Wieler, R., 2009, Complex multiple cosmogenic nuclide concentration and histories in the arid Rio Lluta catchment, northern Chile: Earth Surface Processes and Landforms, v. 34, p. 398- 412.
53. Larsen, I. J., Almond, P. C., Eger, A., Stone, J. O., Montgomery, D. R., & Malcolm, B. (2014). Rapid soil production and weathering in the Southern Alps, New Zealand. Science, 343(6171), 637-640.
54. Matmon, A. S., Bierman, P., Larsen, J., Southworth, S., Pavich, M., Finkel, R., and Caffee, M., 2003, Erosion of an ancient mountain range, the Great Smoky Mountains, North Carolina and Tennessee: American Journal of Science, v. 303, p. 817-855.
55. Meyer, H., Hetzel, R., & Strauss, H. (2010). Erosion rates on different timescales derived from cosmogenic 10Be and river loads: implications for landscape evolution

in the Rhenish Massif, Germany. International Journal of Earth Sciences, 99(2), 395-412.

56. Miller, S. R., Sak, P. B., Kirby, E., & Bierman, P. R. (2013). Neogene rejuvenation of central Appalachian topography: Evidence for differential rock uplift from stream profiles and erosion rates. Earth and Planetary Science Letters, 369, 1-12.
57. Morel, P., von Blackenburg, F., Schaller, M., Kubik, P. W., and Hinderer, M., 2003, Lithology, landscape dissection and glaciation controls on catchment erosion as determined by cosmogenic nuclides in river sediment (the Wutach Gorge, Black Forest): Terra Nova, v. 15, no. 6, p. 398-404.
58. Nichols, K. K., Bierman, P. R., & Rood, D. H. (2014). 10Be constrains the sediment sources and sediment yields to the Great Barrier Reef from the tropical Barron River catchment, Queensland, Australia. Geomorphology, 224(0), 102-110. doi:http://dx.doi.org/10.1016/j.geomorph.2014.07.019
59. Nichols, K. K., Bierman, P. R., Eppes, M. C., Caffee, M., Finkel, R., and Larsen, J., 2005b, Late Quaternary history of the Chemehuevi Mountain piedmont, Mojave Desert, deciphered using ^{10}Be and ^{26}Al: American Journal of Science, v. 305, no. 5, p. 345-368.
60. Nichols, K. K., Bierman, P. R., Eppes, M. C., Caffee, M., Finkel, R., and Larsen, J., 2007, Timing of surficial process changes down a Mojave Desert piedmont: Quaternary Research, v. 68, no. 1, p. 151-161.
61. Nichols, K. K., Bierman, P. R., Hooke, R. L., Clapp, E., and Caffee, M., 2002, Quantifying sediment transport on desert piedmonts using ^{10}Be and ^{26}Al: Geomorphology, v. 45, no. 1,2, p. 89-104.
62. Nichols, K., Bierman, P., Finkel, R., and Larsen, J., 2005a, Long-Term (10 to 20 kyr) Sediment Generation Rates for the Upper Rio Chagres Basin Based on Cosmogenic ^{10}Be, *in* Harmon, R. S., ed., The Rio Chagres: A Multidisciplinary Profile of a Tropical Watershed, Kluwer Academic Publishers.
63. Norton, K. P., Blanckenburg, F. v., and Kubik, P. W., 2010, Cosmogenic nuclide-derived rates of diffusive and episodic erosion in the glacially sculpted upper Rhone Valley, Swiss Alps: Earth Surface Processes and Landforms, v. 35, p. 651-662.
64. Norton, K. P., Blanckenburg, F. v., Schlunegger, F., Schwab, M., and Kubik, P. W., 2008, Cosmogenic nuclide-based investigation of spatial erosion and hillslope channel coupling in the transient foreland of the Swiss Alps: Geomorphology, v. 95, p. 474-486.
65. Norton, K. P., von Blanckenburg, F., & Kubik, P. W. (2010). Cosmogenic nuclide-derived rates of diffusive and episodic erosion in the glacially sculpted upper Rhone Valley, Swiss Alps. Earth surface processes and landforms, 35(6), 651-662.
66. Norton, K. P., von Blanckenburg, F., DiBiase, R., Schlunegger, F., & Kubik, P. W. (2011). Cosmogenic 10Be-derived denudation rates of the Eastern and Southern European Alps. International Journal of Earth Sciences, 100(5), 1163-1179.

67. Ouimet, W. B., Whipple, K. X., and Granger, D. E., 2009, Beyond threshold hillslopes: Channel adjustment to base-level fall in tectonically active mountain ranges: Geology, v. 37, no. 7, p. 579-582.
68. Palumbo, L., Hetzel, R., Tao, M., & Li, X. (2011). Catchment-wide denudation rates at the margin of NE Tibet from in situ-produced cosmogenic 10Be. Terra Nova, 23(1), 42-48.
69. Palumbo, L., Hetzel, R., Tao, M., and Li, X., 2009, Topographic and lithologic control on catchment-wide denudation rates derived from cosmogenic ^{10}Be in two mountain ranges at the margin of NE Tibet: Geomorphology, v. 117, p. 130-142.
70. Perg, L., Anderson, R., and Finkel, R., 2003, Use of cosmogenic radionuclides as a sediment tracer in the Santa Cruz littoral cell, California, USA: Geology, v. 31, p. 299-302.
71. Placzek, C. J., Matmon, A., Granger, D. E., Quade, J., and Niedermann, S., 2010, Evidence for active landscape evolution in the hyperarid Atacama from multiple terrestrial cosmogenic nuclides: Earth and Planetary Science Letters, v. 295, p. 12-20.
72. Placzek, C., Granger, D. E., Matmon, A., Quade, J., & Ryb, U. (2014). Geomorphic process rates in the central Atacama Desert, Chile: Insights from cosmogenic nuclides and implications for the onset of hyperaridity. *American Journal of Science, 314*(10), 1462-1512.
73. Portenga, E. W., & Bierman, P. R. (2011). Understanding Earth’s eroding surface with 10 Be. *GSA Today, 21*(8), 4-10.
74. Pupim, F. d. N., Bierman, P. R., Assine, M. L., Rood, D. H., Silva, A., & Merino, E. R. (2015). Erosion rates and landscape evolution of the lowlands of the Upper Paraguay river basin (Brazil) from cosmogenic 10Be. *Geomorphology, 234*(0), 151-160. doi:http://dx.doi.org/10.1016/j.geomorph.2015.01.016
75. Quigley, M., Sandiford, M., Fifield, K., and Alimanovic, A., 2007a, Bedrock erosion and relief production in the northern Flinders Ranges, Australia: Earth Surface Processes and Landforms, v. 32, no. 6, p. 929.
76. Quigley, M., Sandiford, M., Fifield, L. K., and Alimanovic, A., 2007b, Landscape responses to intraplate tectonism: Quantitative constraints from ^{10}Be nuclide abundances: Earth and Planetary Science Letters, v. 261, no. 1-2, p. 120-133.
77. Reinhardt, L. J., Bishop, P., Hoey, T. B., Dempster, T. J., and Sanderson, D. C. W., 2007, Quantification of the transient response to base-level fall in a small mountain catchment: Sierra Nevada, southern Spain Journal of Geophysical Research, v. 112, no. F03S05, p. 20.
78. Reuter, J. M., 2005, Erosion rates and patterns inferred from cosmogenic ^{10}Be in the Susquehanna River basin: University of Vermont, 172 p.
79. Riebe, C. S., Kirchner, J. W., and Finkel, R. C., 2003, Long-term rates of chemical weathering and physical erosion from cosmogenic nuclides and geochemical mass balance: Geochimica et Cosmochimica Acta, v. 67, no. 22, p. 4411-4427.

80. Riebe, C. S., Kirchner, J. W., Granger, D. E., and Finkel, R. C., 2000, Erosional equilibrium and disequilibrium in the Sierra Nevada, inferred from cosmogenic ^{26}Al and ^{10}Be in alluvial sediment: Geology, v. 28, no. 9, p. 803-806.
81. Riebe, C. S., Kirchner, J. W., Granger, D. E., and Finkel, R. C., 2001a, Minimal climatic control on erosion rates in the Sierra Nevada, California: Geology, v.29, no. 5, p. 447-450.
82. Riebe, C. S., Kirchner, J. W., Granger, D. E., and Finkel, R. C., 2001b, Strong tectonic and weak climatic control of long-term chemical weathering rates: Geology, v. 29, no. 6, p. 511–514.
83. Safran, E. B., Bierman, P. R., Aalto, R., Dunne, T., Whipple, K. X., and Caffee, M., 2005, Erosion rates driven by channel network incision in the Bolivian Andes: Earth Surface Processes and Landforms, v. 30, no. 8, p. 1007-1024.
84. Schaller, M., von Blanckenburg, F., Hovius, N., and Kubik, P. W., 2001, Large-scale erosion rates from In situ-produced cosmogenic nuclides in European river sediments: Earth and Planetary Science Letters, v. v. 188, p. 441-458.
85. Scharf, T. E. (2012). Denudation rates and geomorphic evolution of the Cape Mountains, determined by the analysis of the in situ-produced cosmogenic 10BE. University of Cape Town.
86. Stock, G. M., Frankel, K. L., Ehlers, T. A., Schaller, M., Briggs, S. M., and Finkel, R. C., 2009, Spatial and temporal variations in denudation of the Wasatch Mountains, Utah, USA: Lithosphere, v. 1, no. 1, p. 34-40.
87. Sullivan, C. L., 2007, ^{10}Be erosion rates and landscape evolution of the Blue Ridge Escarpment, southern Appalachian Mountains: University of Vermont, 76 p.
88. Tomkins, K. M., Humphreys, G. S., Wilkinson, M. T., Fink, D., Hesse, P. P., Doerr, S. H., Shakesby, R. A., Wallbrink, P. J., and Blake, W. H., 2007, Contemporary versus long- term denudation along a passive plate margin: the role of extreme events: Earth Surface Processes and Landforms, v. 32, no. 7, p. 1013.
89. Vanacker, V., von Blanckenburg, F., Hewawasam, T., and Kubik, P. W., 2007, Constraining landscape development of the Sri Lankan escarpment with cosmogenic nuclides in river sediment: Earth and Planetary Science Letters, v. 253, no. 3-4, p. 402-414.
90. Vance, D., Bickle, M., Ivy-Ochs, S., and Kubik, P. W., 2003, Erosion and exhumation in the Himalaya from cosmogenic isotope inventories of river sediments: Earth and Planetary Science Letters, v. 206, p. 273-288.
91. von Blanckenburg, F., Hewawasam, T., and Kubik, P. W., 2004, Cosmogenic nuclide evidence for low weathering and denudation in the wet, tropical highlands of Sri Lanka: Journal of Geophysical Research, v. 109, no. F3.
92. Willenbring, J. K., Codilean, A. T., & McElroy, B. (2013). Earth is (mostly) flat: Apportionment of the flux of continental sediment over millennial time scales. Geology, 41(3), 343-346.

93. Willenbring, J. K., Codilean, A. T., & McElroy, B. (2013). Earth is (mostly) flat: Apportionment of the flux of continental sediment over millennial time scales. *Geology, 41*(3), 343-346.
94. Wittmann, H., Blanckenburg, F. v., Guyot, J. L., Maurice, L., and Kubik, P. W., 2009, from source to sink: Preserving the cosmogenic ^{10}Be-derived denudation rate signal of the Bolivian Andes in sediment of the Beni and Mamoré foreland basins: Earth and Planetary Science Letters, v. 288, p. 463-474.
95. Wittmann, H., Blanckenburg, F. v., Kruesmann, T., Norton, K. P., and Kubik, P. W., 2007, Relation between rock uplift and denudation from cosmogenic nuclides in river sediment in the Central Alps of Switzerland: Journal of Geophysical Research, v. 112, no. F04010.
96. Wittmann, H., von Blanckenburg, F., Maurice, L., Guyot, J.-L., Filizola, N., & Kubik, P. W. (2010). Sediment production and delivery in the Amazon River basin quantified by in situ-produced cosmogenic nuclides and recent river loads. Geological Society of America Bulletin. doi:10.1130/b30317.1
